# Functionalization of Polyethyleneimine with Hollow Cyclotriveratrylene and Its Subsequent Supramolecular Interaction with Doxorubicin

**DOI:** 10.3390/molecules25225455

**Published:** 2020-11-20

**Authors:** Carmine Coluccini, Yoke Mooi Ng, Yves Ira A. Reyes, Hsin-Yi Tiffany Chen, Yit Lung Khung

**Affiliations:** 1 Institute of New Drug Development, China Medical University, No. 91 Hsueh-Shih Road, Taichung 40402, Taiwan; yumei.wu91@gmail.com; 2 Department of Engineering and System Science, National Tsing Hua University, Hsinchu 30013, Taiwan; yvesirareyes@gmail.com (Y.I.A.R.); hsinyi.tiffany.chen@gapp.nthu.edu.tw (H.-Y.T.C.); 3 Department of Biological Science and Technology, No. 100, Jingmao 1st Rd, Beitun District, Taichung City 406, Taiwan

**Keywords:** armed Cyclotriveratrylene, supramolecular interaction, Doxorubicin loading

## Abstract

In this paper, a modified Cyclotriveratrylene was synthesized and linked to a branched Polyethylenimine, and this unique polymeric material was subsequently examined as a potential supramolecular carrier for Doxorubicin. Spectroscopic analysis in different solvents had shown that Doxorubicin was coordinated within the hollow-shaped unit of the armed Cyclotriveratrylene, and the nature of the host–guest complex revealed intrinsic Van der Waals interactions and hydrogen bonding between the host and guest. The strongest interaction was detected in water because of the hydrophobic effect shared between the aromatic groups of the Doxorubicin and Cyclotriveratrylene unit. Density functional theory calculations had also confirmed that in the most stable coordination of Doxorubicin with the cross-linked polymer, the aromatic rings of the Doxorubicin were localized toward the Cyclotriveratrylene core, while its aliphatic chains aligned closer with amino groups, thus forming a compact supramolecular assembly that may confer a shielding effect on Doxorubicin. These observations had emphasized the importance of supramolecular considerations when designing a novel drug delivery platform.

## 1. Introduction

**Figure 1:**
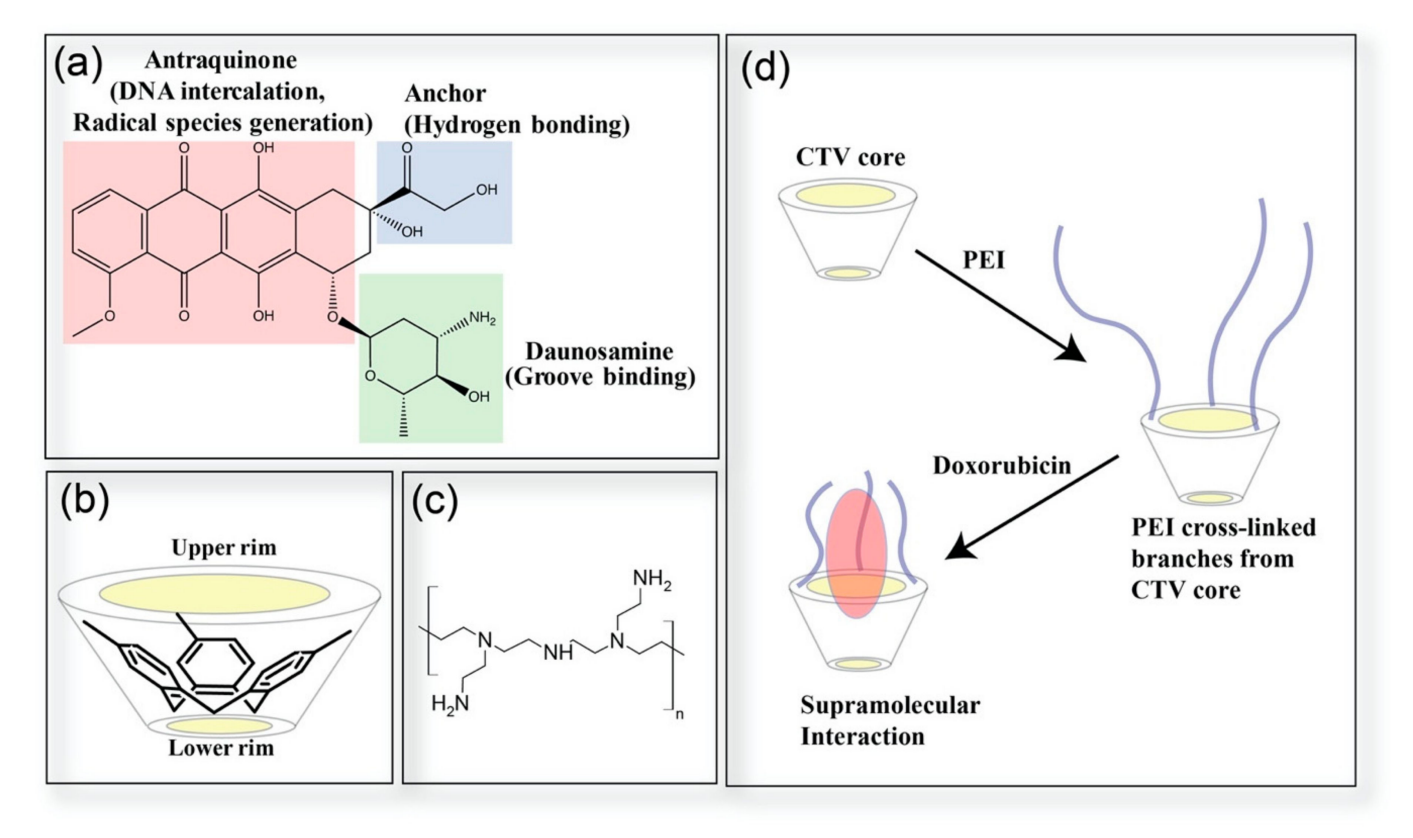
Molecular structures involved in this work: (**a**) Doxorubicin and biochemical function of each single molecular unit; (**b**) Cyclotriveratrylen (CTV) in the stable cup-shaped conformation; (**c**) Polyethylenimine; (**d**) Adduct Polyethylenimine (PEI)-CTV and Doxorubicin coordination.

One of the important concerns for many drug systems may be the non-discriminating toxicity that may sometimes result in collateral damage to healthy tissues and organs [1]. This has subsequently provided impetus for research into newer drug-delivery platforms with the aims of limiting the cytotoxic side effect of the drugs [2,3] as well as improving on delivery efficacy. Of the many important clinical drugs that should sometimes be given due considerations during designing advanced drug delivery systems, Doxorubicin is an important anti-cancer drug of complex molecular structure (Figure 1a), and it possesses different coordinating and reactive groups that would work in tandem to intercalate and damage DNA double-strands, thus inducing cellular apoptosis [4,5,6]. While the drug is highly potent, it also carries the risk of cardiotoxicity, as it had been shown to damage healthy cardiomyocytes (cardiac muscle cells) [5,7,8,9,10]. So far, the main strategies for the coordination and delivery of Doxorubicin involves the supramolecular encapsulation [11,12,13,14] as well as the coordination by a labile covalent bond that is possible through the exploitation of the amino group within the Daunosamine unit [15,16,17,18,19]. In many drug delivery systems, supramolecular coordination is often more preferred over other interactions type due to its better response in the presence of external stimuli that could be further finetuned through the modulation of the coordination/releasing processes [20].

Doxorubicin is a structurally complex molecule capable forming a range of multifaceted supramolecular interactions, rendering it as an excellent guest target for novel drug delivery designs. The preparation of a hosting system for the encapsulation of Doxorubicin may prove to be both interesting and useful for developing smart and high-performance synthetic drug delivery systems while reducing its cytotoxicity, and the very nature of the supramolecular interaction also forms the basis of our work here.

**Figure 2:**
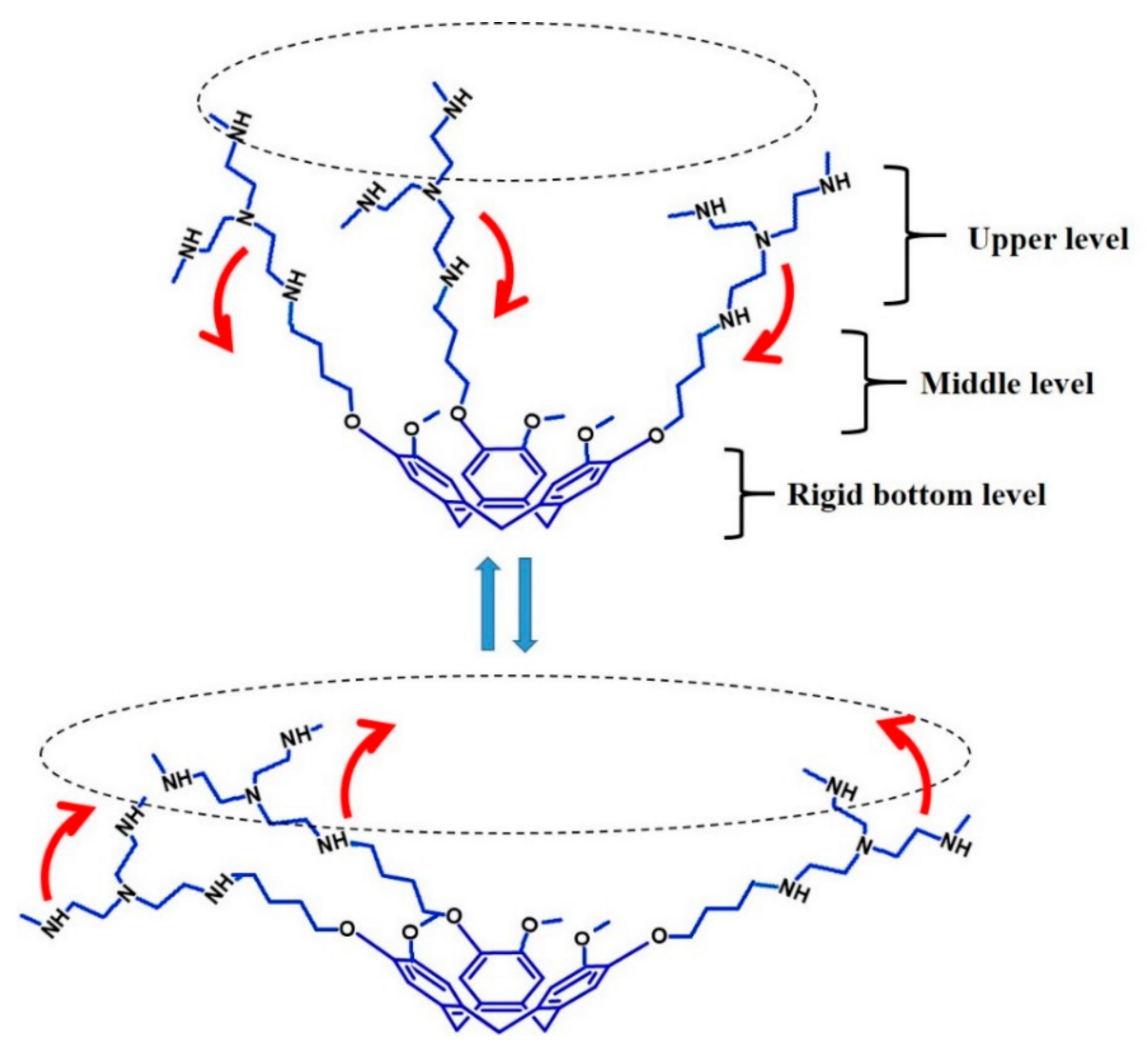
A pictorial description of the hollow structure inside the polymeric network. The flexibility of the alkyl chains that arm the CTV unit induces the variability of the diameter of the hollow unit and the hosting space shape.

In this paper, we present an assembly of two molecular modules, Cyclotriveratrylene (CTV) and branched Polyethylenimine (PEI), with completely different coordinating properties that would interact with Doxorubicin to form a host–guest complex, and the illustration of their individual structures are as shown in Figure 1b,c. In brief, we proposed a joint molecular architecture comprising of a molecular CTV core that was modified with PEI (branch) to form a pseudo “cup-like” arrangement (Figure 1d) that would eventually form supramolecular interaction with Doxorubicin. Firstly, the branched PEI polymer consisted of a mixture of primary, secondary, and tertiary amines that can be protonated under a range of pH and can be in turn modified by exploiting the amino group’s reactivity either in its original form or through chemical modifications. PEI has already been widely investigated as the carrier for gene delivery purposes due to the high DNA loading capacity as well as its flexible structural design [21,22,23]. At times, PEI is often conjugated to aromatic molecules to help improve the transfection efficiency as well as for the preparation of fluorescent polymeric nanoparticles [24,25]. More importantly, it has also been reported previously to have interacted with Doxorubicin through covalent connection or via supramolecular coordination after cross-linking with Chenodeoxycholic acid [26,27]. At the core of our assembly is CTV, which is a cyclic trimer with a rigid conformation, and Lindsday et al. had previously described and examined its hollow structural form [28]. Interestingly, CTV often adopts a cup-shape configuration under its most energetically favorable configuration, as shown in Figure 1b with the distance between the centers of the rings estimated at 4.77 Å, while the dimension of the cavity may vary depending on the type of appending side arms to the upper rim [29]. So far, there had been a wide range of supramolecular interactions examined for the CTV host as well as an interesting assortment of guest molecules such as ions and metals [30,31,32,33,34,35,36,37,38,39,40,41,42]. Differently armed Cyclotriveratrylenes had exhibited the capability to coordinate fullerenes, and this was widely reported in the literature [43,44,45,46,47,48,49,50,51]. In terms of interfacing with bioactive molecules, the ability to incorporate flavonoids had also been reported, and these attributes help to advocate for the role of CTV serving as a good supramolecular carrier [52]. Cyclical methylphenylarene holigomers have been reported as likers for drug molecules displaying heteroaromatic rings connected to heteroatom chains [53]. In our design as shown in Figure 2, a molecular assembly comprising of PEI branch-like arms cross-linking with a modified CTV at the core was assembled. The molecular system can be envisaged as a polymeric network where ramified Polyethylenimine chains are chemically connected through hollow molecular structures to form a cup-shaped configuration. It comprised of three different levels: (1) rigid bottom from the CTV aromatic core, (2) a middle domain of alkyl chains, and (3) an upper distal region of rich ethylamine groups. The flexibility from the aliphatic nature of the middle and the upper domain help confer a certain degree of variability in terms of the molecular dimension, and this enables a certain amount of shape adjustment during the optimization process with the incoming guest molecule. It also implied that the coordination of Doxorubicin within the hollow core can be described with an induced fit model that is similar to that of the substrate interacting with enzymatic units in many biological settings. Figure 2 also helps illustrate the conformational flexibility of the proposed cross-linked polymeric structure from the aromatic core. It is hypothesized that all three levels of our molecular assembly will interact differently with the guest Doxorubicin. The aromatic core should in principle favor for a hydrophobic interaction with the aromatic component of the Doxorubicin, while a flexible PEI chain will present for the opportunity for Van der Waals interaction due to the amino groups’ coordination between heteroatoms via hydrogen bonding. The overall system has been specially designed to help coordinate/intercalate the guest Doxorubicin within the molecular assembly. Hence, in this report, our main focus was to examine upon the various aspects of the molecular assembly and subsequently describe the overall guest–host interaction of our system. The analysis of the interaction was determined via UV-vis and ^1^H-NMR spectroscopy, while the nature of the interaction was examined under different solvents as well as the correlations of their individual contribution toward the overall interaction process. Our data were also complemented by Density functional theory (DFT) calculations to further substantiate our claims of the modality of drug absorption inside the hollow structure of CTV.

## 2. Methods and Results

### 2.1. Synthesis of the Cross-Linker

**Scheme 1:**
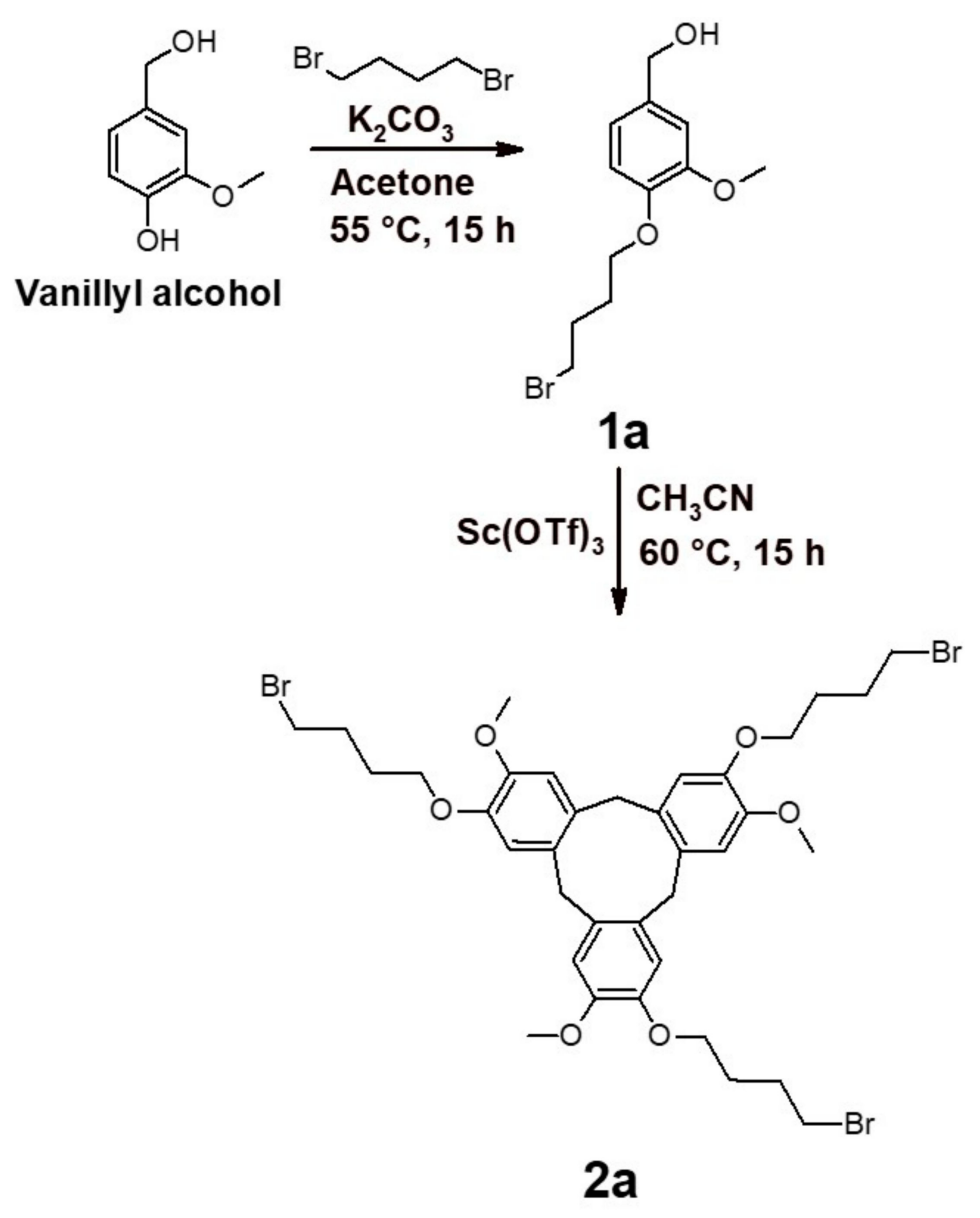
Synthesis of the cyclic cross-linker **2a**.

The synthesis of the monomer **1a** (Scheme 1) has been carried out by the reaction of commercially available vanillyl alcohol with 1,4-dibromobutane in the alkaline solution. From previous literature, the reaction of vanillyl alcohol with dibromoalkanes had been reported under a range of different conditions [54,55,56,57]. However, we were able to obtain good yields in acetone and with potassium carbonate (Figures S1–S3 showed both the ^1^H-NMR and ^13^C-NMR spectra as well as the details for the characterization). The attainment of the cyclic compound **2a** (Scheme 1) was achieved via the reaction of **1a** with Scandium Triflate in acetonitrile at 60 °C (see Figures S4–S6 for the ^1^H-NMR, ^13^C-NMR spectra as well as the mass spectrometry analysis in S6). The molecule **2a** was a cyclic trimer with a cup-like three-dimensional shape, and this was as evidenced by the ^1^H-NMR spectrum and mass spectrometry analysis. The cup shape of CTV was usually indicated by the ^1^H-NMR signal of the –CH_2_- between the aromatic rings that is a doublet. It is important to note that under the chair conformation usually adopted by the tetramer, the signal is typically a broad singlet. The number of detectable monomeric fragment by mass spectrometry also corresponds well with the molecular weight of the deduced trimeric product. We had also verified the formation of the isomer **2a** as shown in the Scheme 1 through Nuclear Overhauser effect (NOE) NMR analysis (Figure S7).

### 2.2. Preparation of the Cross-Linked Polymer

**Scheme 2:**
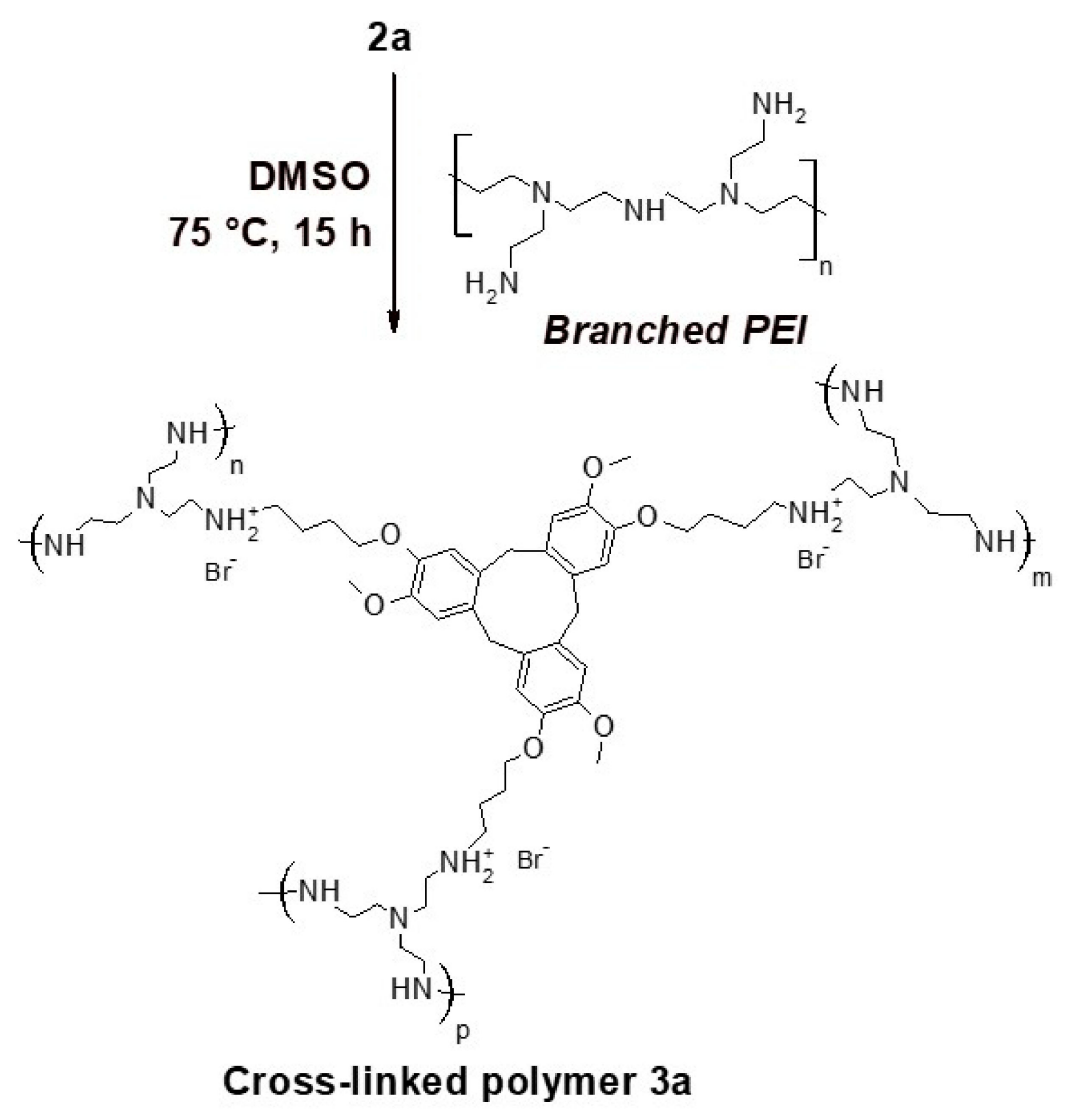
Synthesis of cross-linked polymer **3a**.

The cross-linking was performed by mixing compound **2a** with PEI in dry DMSO at 75 °C, as depicted in Scheme 2. The conditions that we utilized had been previously reported in the literature for the specific reaction of PEI with alkyl tosylates [58]. We had noticed that upon formation of the product, it was only partially soluble in many of our solvent systems used. The ^1^H-NMR spectrum in DMSO-*d_6_*, MeOD, D_2_O of product **3a** had exhibited broadened signals within the chemical shift ranges with **2a**, while PEI displayed sharp and well-defined peaks, in particular the broadened aromatic signal at 6.7–7.4 ppm as well as the series of aliphatic signals at 3.5–5.0 ppm, 2.0–3.0 ppm, and 1.0–2.0 ppm (see Figures S8–S12 for the ^1^H-NMR spectra of **2a**, **3a** as well as for PEI). All ^1^H-NMR spectra also exhibited a broad signal at 8.1 ppm because the cross-linking process generates HBr, which converts some of the amino groups of PEI in ammonium ion. Interestingly, the cross-linked polymer **3a** had exhibited a UV absorption maximum at 375 nm in methanol, DMSO, and water, while the precursors **2a** and PEI had shown no absorption maxima in the range 300–700 nm (see Figure S13 for the UV-vis spectra of **2a**, **3a** and PEI in MeOH, DMSO, and water). In the Fournier-Transform Infrared spectroscopy (FT-IR) spectra of **3a** shown Figure S14, the absorption peak at 555 cm^−1^, which is indicative of the C-Br bonding, was not detectable, while this peak was prevalent in the FT-IR for **2a**. Further evidence could be derived from the ratio between the intensity of amine vibration band at 3400 cm^−1^ and aliphatic –CH– at 2930 cm^−1^, which was higher in the FT-IR spectrum for **3a** than compared to that of PEI.

### 2.3. Coordination of Doxorubicin

**Figure 3:**
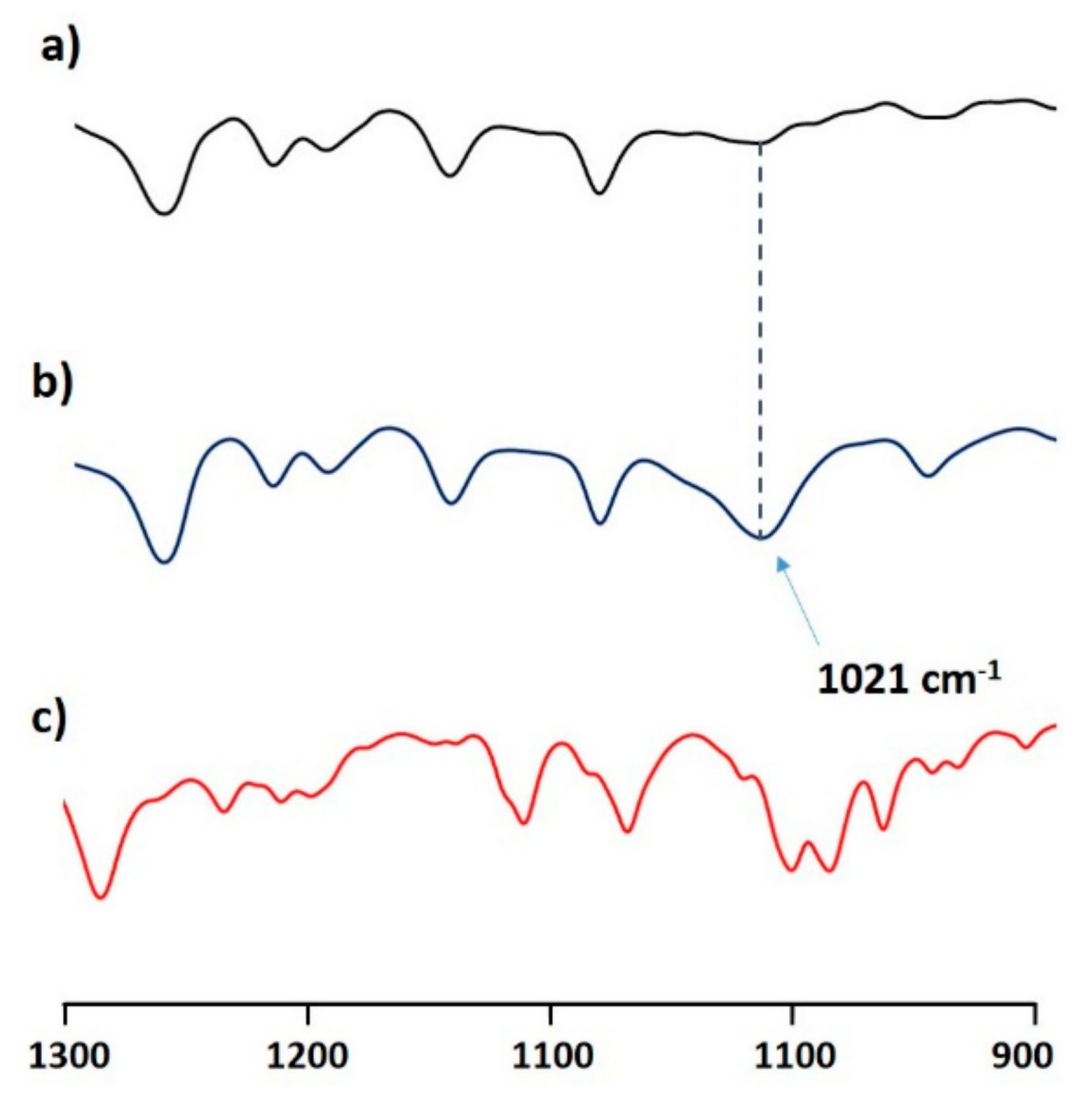
Fourier-Transformed Infrared spectra of (**a**) **3a** mixed with Doxorubicin, (**b**) **3a** not mixed with Doxorubicin, (**c**) Doxorubicin.

**FT-IR analysis.** The cross-linked polymer was first mixed with Doxorubicin in Methanol, and after the evaporation of the solvent, the FT-IR spectra was subsequently obtained. The spectrum was found to be similar to that of cross-linked polymer in the absence of Doxorubicin, and the only notable difference being the reduction of a peak at 1021 cm^−1^ (Figure 3 displayed the spectral range of 900–1300 cm^−1^ while Figure S15a–c presented the spectral range of 400–4000 cm^−1^). This was assigned to that of the saturated C-O stretching from the phenol-ethers [59]. The subsequent mixing of Doxorubicin with PEI had produced a spectrum that collectively exhibited the signals of the two respective compounds (Figure S15c–e).

**Figure 4:**
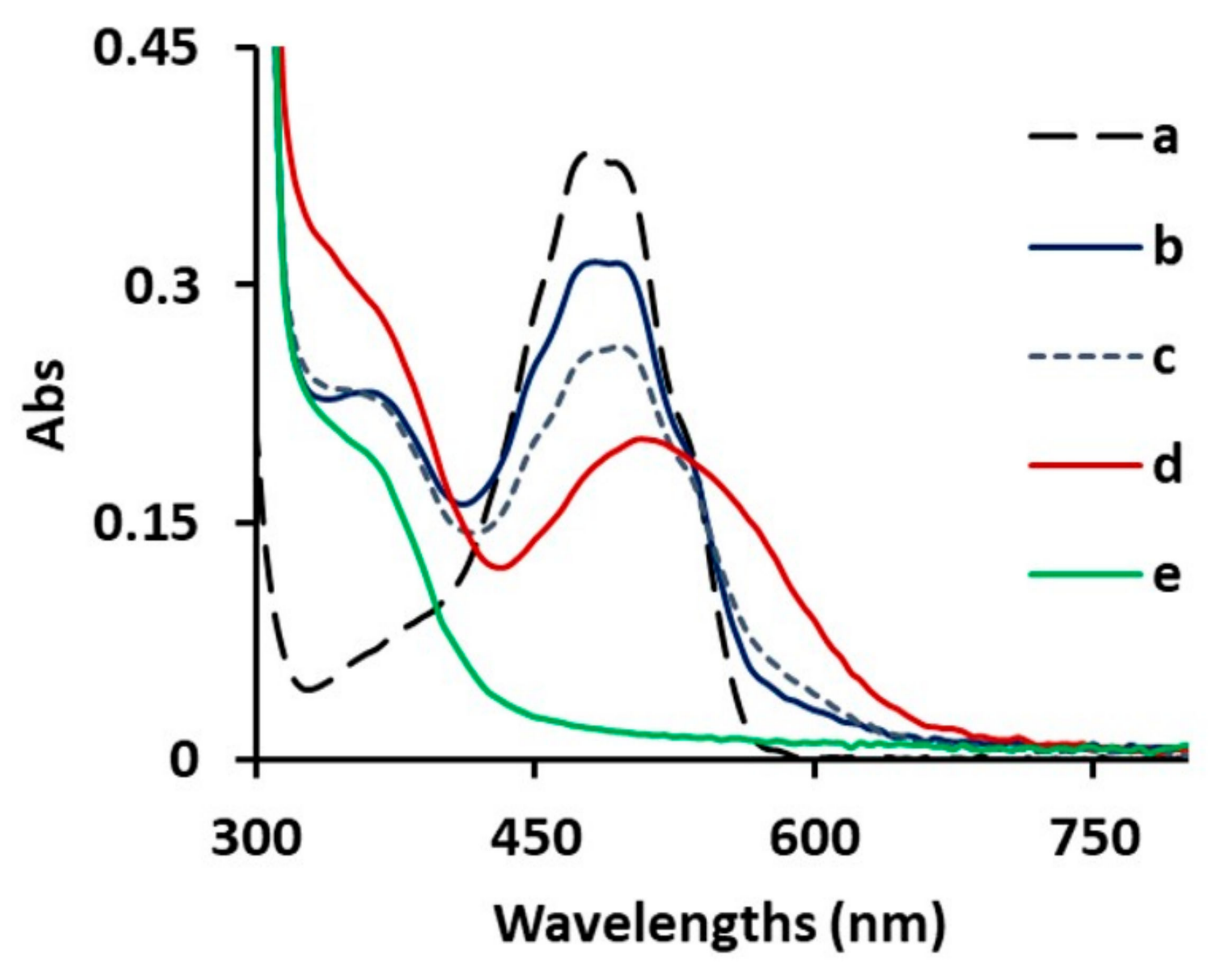
UV-vis spectra of Doxorubicin 2.9 × 10^−5^ M in H_2_O: (**a**) solution not titrated. (**b**) One mL of solution titrated with 3 mg of **3a**. (**c**) One mL of solution titrated with 3 mg of **3a** and aged for 5 days. (**d**) One mL of solution titrated with 3 mg of **3a** and aged for 25 days. (**e**) UV spectra of 3a in water, 3 mg/mL.

**UV-vis spectra.** From a series of UV-Vis measurements, the presence of Doxorubicin was observed to exhibit an absorption maxima at around 475–495 nm in methanol, DMSO, and water, and this was as shown in Figure S16 and Figure 4. On the other hand, Compound **3a** registered an absorption at 340–420 nm in all three solvents, without displaying a clear maximum, while PEI and compound **2a** did not show any notable absorbance (Figure S13). Similarly, in Figure S16 and Figure 4, the UV-vis spectra of Doxorubicin 2.9 × 10^−5^ M mixed with **3a** were also recorded.

In MeOH, the addition of 3 mg of **3a** to a 2.9 × 10^−5^ M solution of Doxorubicin (Figure S16a) had resulted in the appearance of a maximum peak at 380 nm, and a little reduction of Doxorubicin absorbance was observed. The aging of the solution produced a diminution of the Doxorubicin absorption maximum as well as a change of the curve shape, as well as the disappearing of the maximum at 380 nm. In DMSO, no reduction of Doxorubicin has been observed, and the spectrum displays the two curves of the polymer and drug (Figure S16b), the aging of solution produces an alteration in the curve of the functionalized polymer, and there are no changes in the spectrum of Doxorubicin. The aging of the solution of pure polymer **3a** had produced no changes in the UV spectrum (Figure S17). A titration experiment was performed on the methanol solution and the variation of the absorption maximum at 380 nm vs the concentration of the CTV units incorporated in the polymer **3a** was plotted. As a result, we obtained that only 1/1000 of the CTV unit will coordinate the Doxorubicin, and the constant of coordination is 100 (the details of the experiment are reported in Figure S18). The addition of 5 mg of **3a** to 1 mL of the solution had resulted in a suppression of the Doxorubicin absorption that was not significantly incremented by the addition of 6 and 7 mg (Figure S19). The aging for 25 days of the mixture had resulted in a redshift that is typically reported when Doxorubicin interacts with a hosting system through non-covalent forces, and the UV data suggest a weak Van der Waals-like interaction of Doxorubicin with compound **3a** [60]. In DMSO, the addition of 5 mg of **3a** to 1 mL of Doxorubicin (2.9 × 10^−5^ M) and the quenching of this solution did not significantly change the UV spectrum (Figure S20). Interestingly, as shown in Figure 4, the reduction of the Doxorubicin absorbance under similar concentration was observed in water, even when 3 mg of **3a** was added to 1 mL of solution, and also the appearance of a maximum at 367 nm was observed. After 5 days further quenching of the Doxorubicin, absorption is observed. Yet, with the subsequent aging of the solution, over 25 days, there was a gradual broadening of the Doxorubicin absorption, and a red-shift was registered. This change of the absorption curve in water was in fact a strong indicator for the strengthened interaction between aromatic rings of Doxorubicin and **3a**, as previously reported in literature [61,62,63], which was probably through hydrophobic processes. A plot of the variation of the absorption maximum at 367 nm vs concentration of CTV units incorporated in **3a** polymer has been performed, and as result, it was obtained that the coordination between CTV and Doxorubicin follows a stoichiometry 1:1 and the constant is 10^5^ (Figure S21). The pure water solutions of **3a** and Doxorubicin are not affected in the absorption profile by the aging (Figure S22). Under methanol, the addition of PEI to Doxorubicin had generated a reduction of absorbance of 110 M^−1^ cm^−1^ (Figure S23a). In DMSO, in addition to a stronger lowering of Doxorubicin absorbance relative to the methanol solution, a new absorption peak at 600 nm (Figure S23b) was also observed. Consequently, the addition of PEI in water had redshifted the Doxorubicin maximum absorption peak (Figure S23c). All UV-vis data based on the various conditions had been listed and tabulated accordingly in Table 1.

**^1^H-NMR analysis.** The ^1^H NMR spectra was taken for Doxorubicin in DMSO-*d*_6_, MeOD, and D_2_O (Figures S24–S26). The assignments of the Doxorubicin signature had been made in accordance to previous reports in both deuterated Dimethyl Sulfoxide and Water [64,65], while assignments in Methanol had been made on the basis of similarity based on other solvents. Firstly, the phenolic –OH signals from the hydroquinone unit and –NH_3_^+^ were only detectable in DMSO-*d*_6_, while the alcoholic –OH linked to the aliphatic carbons was undetectable only in D_2_O. More importantly, in DMSO-*d*_6_, the –OH signals of the hydroquinone unit and the –NH_3_^+^ group (13.16, 14.00, 7.98 ppm) of Doxorubicin were found to have disappeared with the addition of **3a,** while the signals for the alcoholic –OH (5.45–5.49 ppm) were notably broadened. The aromatic signals were shifted toward higher ppm values with respect to the pure doxorubicin, and the peak shapes were distinctly altered, while the aliphatic signals are shifted at the lowest ppm or not affected by the addition of **3a** (Figure S27). The aging of the solution for 25 days did not have an effect on the signature of Doxorubicin and produced newer signals due to the hypothesized decomposition of **3a** (Figure S28). In MeOD, all signals of Doxorubicin were also clearly broadened (Figure S29). The aging of the solution for 25 days had produced the disappearing of all drug signals, and only those from **3a** were detectable (Figure S30). The pure solution of Doxorubicin in MeOD, as of the pure solution of **3a** in MeOD, are not affected by aging (Figure S31). Figure S32 shows the variations of NMR signals of Doxorubicin in MeOD and DMSO-*d*_6_ when **3a** was added, focusing at the range of 5–9 ppm where all aromatic signals, the –OH linked to aliphatic carbons, and the aliphatic hydrogen were the most prominent.

**Figure 5:**
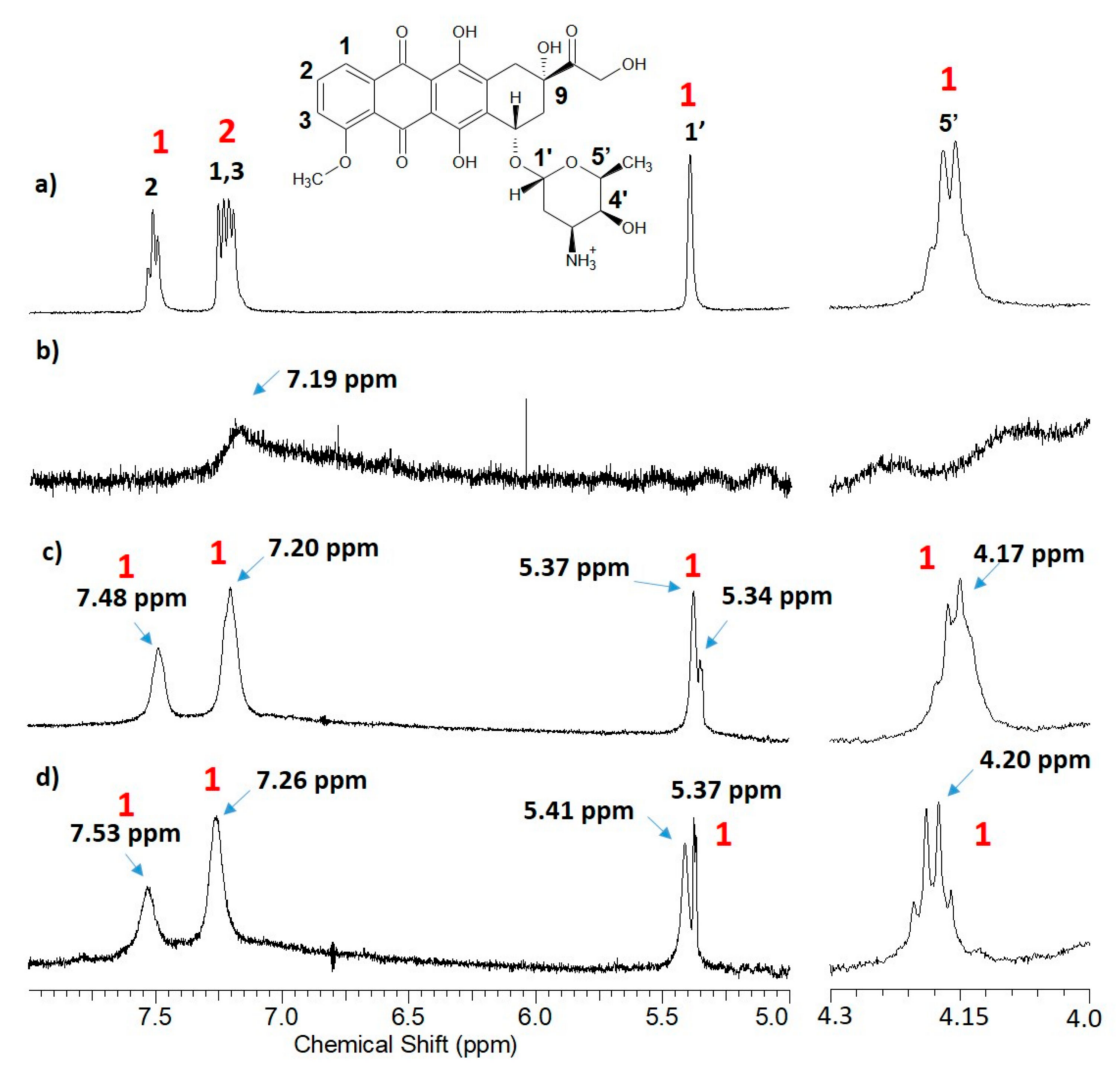
^1^H-NMR spectra in D_2_O, 4–8 ppm: (**a**) Doxorubicin (5 mg); (**b**) Compound **3a** in water; (**c**) Doxorubicin mixed with **3a** (5 mg + 5 mg); (**d**) Doxorubicin mixed with **3a** aged 25 days.

In D_2_O, the addition of **3a** had consequently induced a broadening of the Doxorubicin aromatic signals and with the subsequent aging for 25 days, a shift of the broadened signals at the highest ppm (Figures S33 and S34) was observed. The control analysis of compound **3a** in D_2_O showed that the polymer is not decomposed after aging in D_2_O (Figure S35). Figure 5 revealed how Doxorubicin signals had changed as a result of mixing with **3a** and subsequent changes induced from solution aging. It was clear that there was a gradual broadening of the aromatic signatures after mixing and a change of chemical shift after aging. After the addition of **3a**, a new signal at 5.34–5.37 ppm was registered and had avertedly intensified after aging, and it could be assigned to the alcoholic –OH due to its similarity with the spectra in DMSO-*d*_6_ and MeOD while not being notable in the spectrum of Doxorubicin in D_2_O. It was also notable that the aliphatic signal of the hydrogen at 4.17 ppm after the addition of **3a** and after aging observably changed chemical shift without broadening. After the addition of **3a**, the integration of the aromatic signals of the Doxorubicin is altered. It is clear that the broadening of aromatic peaks of both **3a** and Doxorubicin generates an overlap of signals. 

**Figure 6:**
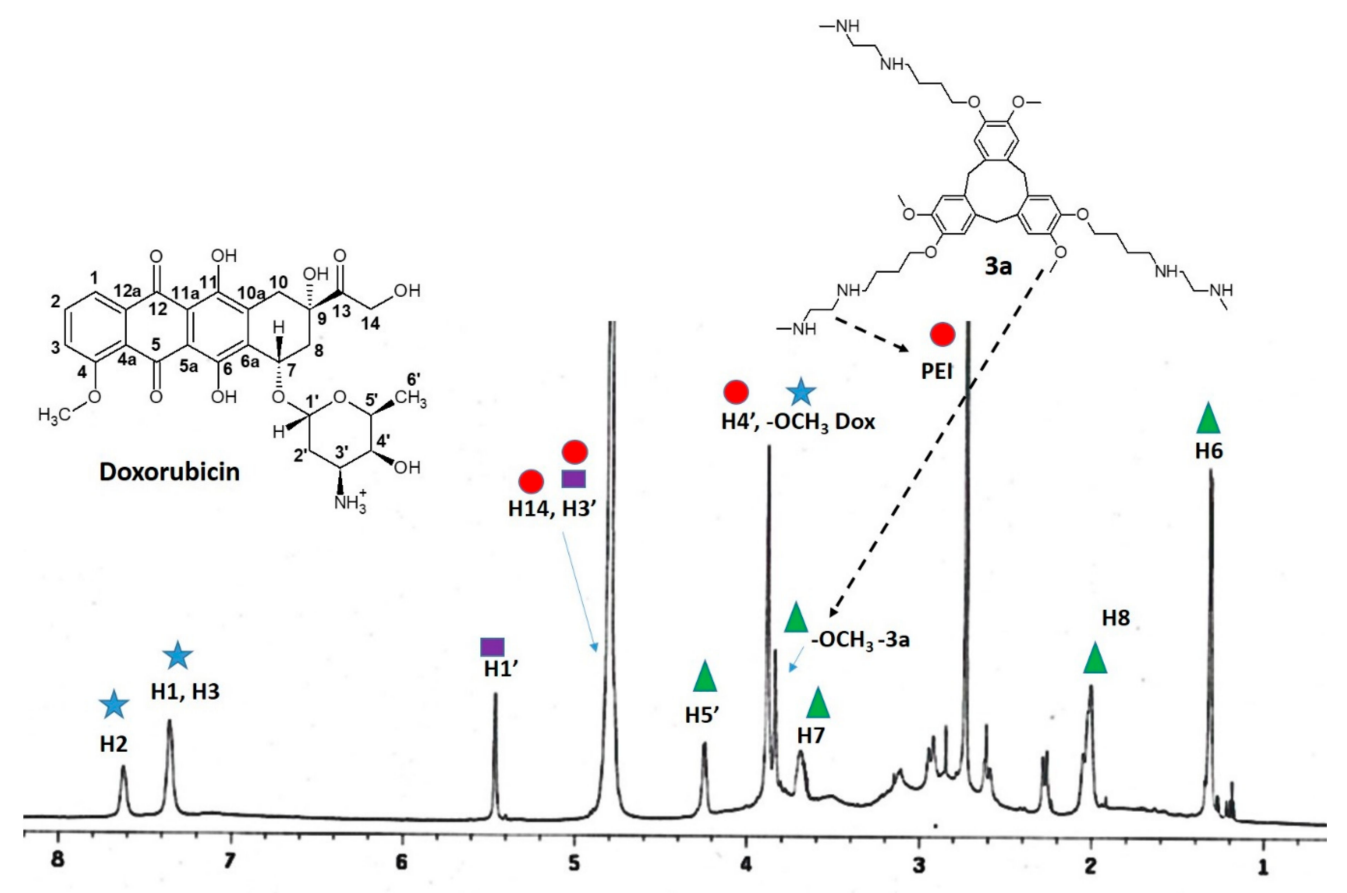
The groups marked with the same symbol are spatially close to each other according to NOE and NOESY NMR spectra.

NOESY and NOE NMR analysis of D_2_O solutions was performed to understand which groups from **3a** and Doxorubicin were involved in the interaction between the two molecules. Figures S35 and S36 show the spectra as well as the detailing of our attempt at mapping out the intermolecular interactions, as highlighted in Figure 6. The groups that were linked with alcoholic –OH would interact with the PEI units, while the –OMe groups of **3a** prevalently interact with the aliphatic hydrogens of Daunosamine and the Anthraquinone groups of Doxorubicin. The aromatic signals of **3a** were completely overlapped with the aromatic groups of Doxorubicin, and the NOE does not provide the information about the coordination of these groups. 

^1^H-NMR spectra have been obtained for Doxorubicin mixed with PEI in the three solvents (Figures S30–S32). In MeOD, the mixing with PEI had generated a non-homogeneous variation of chemical shifts at the lowest ppm as well as a change in the shape of the aromatic signals. In DMSO-*d*_6_, it was difficult to deconvolute individual signals of Doxorubicin, while in D_2_O, the aromatic signals were largely broadened, while the aliphatic region exhibited a complex pattern of signals.

In Table 2, the chemical shifts of aromatic and aliphatic signals of Doxorubicin in the solutions were as shown in situations where these signals were detectable.

In the table, it can be observed that under MeOD and DMSO-*d*_6_, the aliphatic proton H1′ is not affected by **3a**, H5′ is moved at lowest ppm, in D_2_O, both signals are moved at highest chemical shifts after aging. The aromatic signals are moved at the lowest ppm by **3a** in MeOD, and they are moved at the highest in DMSO-*d*_6_ and D_2_O. The addition of PEI in MeOD had consequently shifted all the signals at the lowest ppm.

As described earlier, the hosting system possessed a cavity at the center of the CTV core, and PEI extends out from this core to form a “cup-like” molecular configuration. From our observations, the aromatic groups of Doxorubicin are preferentially orientated at close proximity to the CTV core region and the heteroatoms linked to aliphatic groups would form interactions with the amino groups of **3a**. The aging of solutions subsequently contributed to the pull of the aromatic groups of Doxorubicin at close proximity to the CTV.

Yet in all the solutions used in this work, all molecular units of the hollow structure would coordinate with Doxorubicin, while all the functional groups from Doxorubucin reciprocated in similar fashion during the process of coordination. If the solvent molecules interact with the amino groups of **3a**, the interactions of –NH– with Doxorubicin would be obstructed, and the drug molecule would be pulled toward the CTV core. The detailed analysis of the NMR signals of the protons linked to heteroatoms had allowed us to understand the nature of the interplay between Doxorubicin and solvent molecules. In general, the –NH– and –OH protons are normally sharp and intense in an aprotic-polar solvent such as DMSO but tend to be broadened when the solvent is protic [66]. In DMSO-d_6_ solutions of mixed **3a** and Doxorubicin, the amino groups of the hosting systems were not protonated by the solvent and were free to interact with the protons of Doxorubicin, and this resulted in the disappearance of ammonium and quinol groups in the NMR spectrum as well as the broadening of the alcoholic –OH. The coordination inside the cup-shaped CTV had partially help shielded Doxorubicin from the amino groups, for this reason, there was no change of absorption frequencies observed, even after a period of aging. In the case of mixing with PEI, in the absence of protective CTV, the amino groups would interact strongly with Doxorubicin, and the UV spectrum had revealed the typical red-shifting when anthraquinone colorants were mixed with amines in DMSO [67]. Under methanol, Van der Waals interactions were mostly responsible in the setting of Doxorubicin because the aromatic ^1^H-NMR signals had broadened. The UV-vis analyses demonstrated that the interaction was weak because the addition of **3a** to the drug did not affect the drug absorbance in any significant manner. This was due to the fact that the solvent molecules also interacted with CTV and compete with the drug molecules, and for this reason, polymer **3a** was more soluble in methanol with respect to the other solvents. The interaction of the -NH– groups of **3a** was also weak because it was obstructed by the solvent molecules that interact with these groups; in fact, the addition of PEI not cross-linked had a similar effect on the UV spectra of Doxorubicin. Thus, only through NMR analysis was the interaction of the –NH3+ in Doxorubicin detectable. There was a red-shifting of the absorbance in the aged Methanol solutions of the mixture Doxorubicin-**3a**, as the disappearance of the drug ^1^HNMR signals in the same mixture in the aged MeOD was probably due to a degradative process that **3a** induces on the Doxorubicin (the pure solution of drug as the pure solution of **3a** in MeOD are stable for a long time, as shown in Figure S31). Our ^1^H-NMR spectra correlated with our UV absorbance studies and had in turn confirmed that the drug molecule was indeed slowly encapsulated in a micellar-like hosting structure [65]. The FT-IR analysis had also suggested that in methanol, soon after mixing the drug with **3a**, the doxorubicin was localized at close proximity to aliphatic chains of **3a**, considering that the sample for this analysis had been prepared by mixing **3a** and doxorubicin in methanol and that the reducing of the **3a** phenol-ether signal has been recorded. The interaction between the CTV and Doxorubicin in water was stronger than in the other solvents, as shown by the UV-vis analyses. The water molecules did not interact with the CTV, but only with the –NH– groups and with other water molecules of the solvent. This suggested that the interaction was reinforced by hydrophobic effects. The NOE and NOESY analyses demonstrated that the molecule was orientated with the part that contains the alcoholic groups toward the ethylene amine units and with the phenol-ether unit toward the aromatic rings of CTV. Broadening of the aromatic Doxorubicin signals in D_2_O as with the shift at highest ppm in the presence of **3a** indicated the presence of more than one species undergoing a slow exchange on the ^1^HNMR time scale inside and outside the CTV units [68]. The orientation of the drug molecule that interacted with **3a** had also shown hydrogen bonding interactions between the alcoholic groups of Doxorubicin and amino groups of **3a**. In the mixture of Doxorubicin with only PEI without CTV units, the UV spectrum of Doxorubicin was typical of aqueous solutions at pH ≥ 11 [69], and the presence in the ^1^H-NMR spectrum of many aliphatic peaks as the lowering of aromatic signals could be explained as a result of the formation of different ions of Doxorubicin (PEI is a cationic polymer in water and generates OH- ions). However, it is important to note that the solvent conditions involved in spectroscopic analysis were not compatible to those under biological conditions. Hence, a simulation that reproduces the host–guest supramolecular coordination, net of interaction with the solvent, would provide for a complete picture of the **3a** ability in drug incorporation in different bioenvironments.

### 2.4. Computational Analysis of Supramolecular Assembly

**Figure 7:**
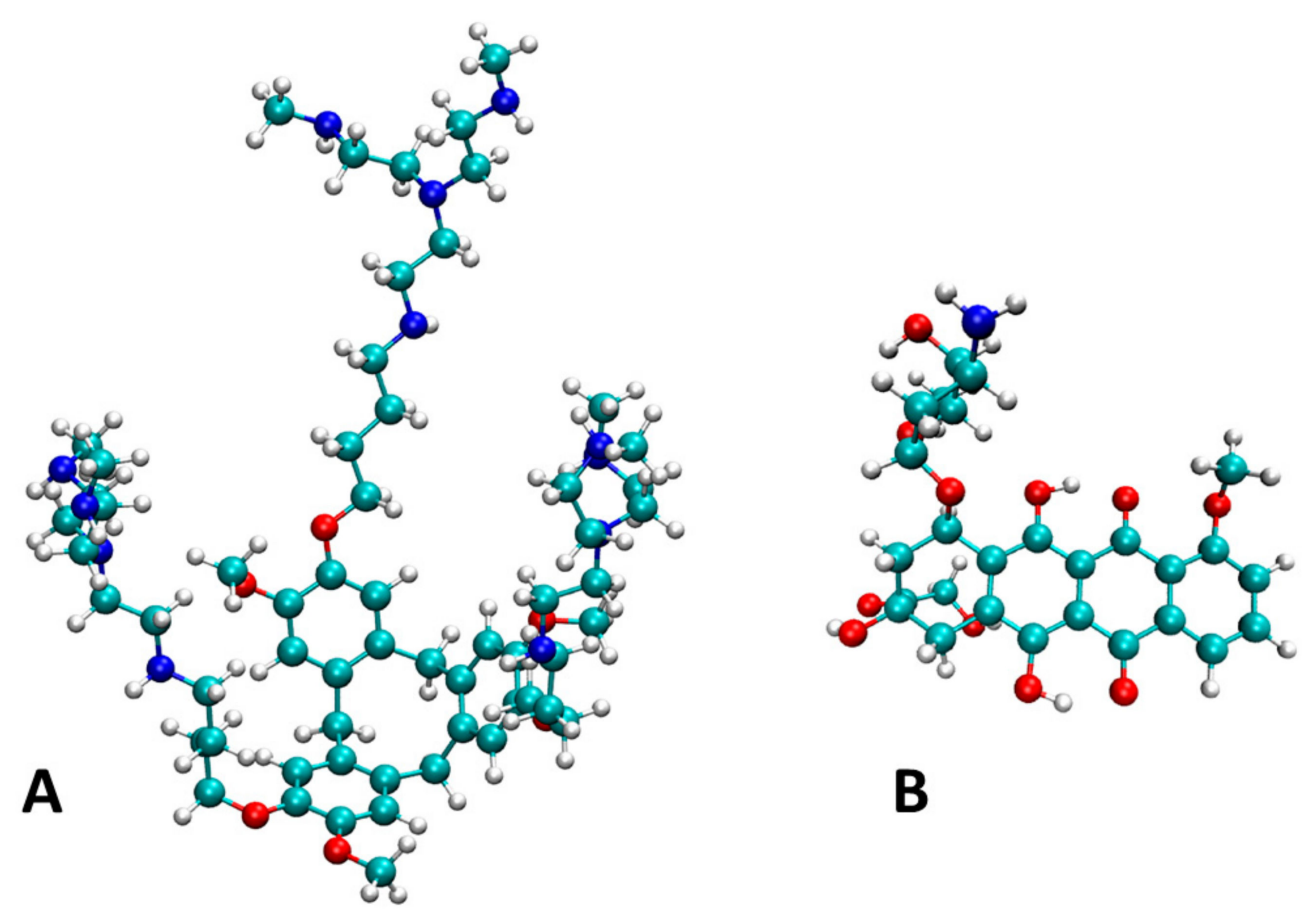
Density functional theory (DFT) optimized structure of (**A**) ethyleneimine–CTV copolymer unit and (**B**) doxyrubicin molecule in ball and stick representation (cyan is carbon, white is hydrogen, red is oxygen, and blue is nitrogen).

**Figure 8:**
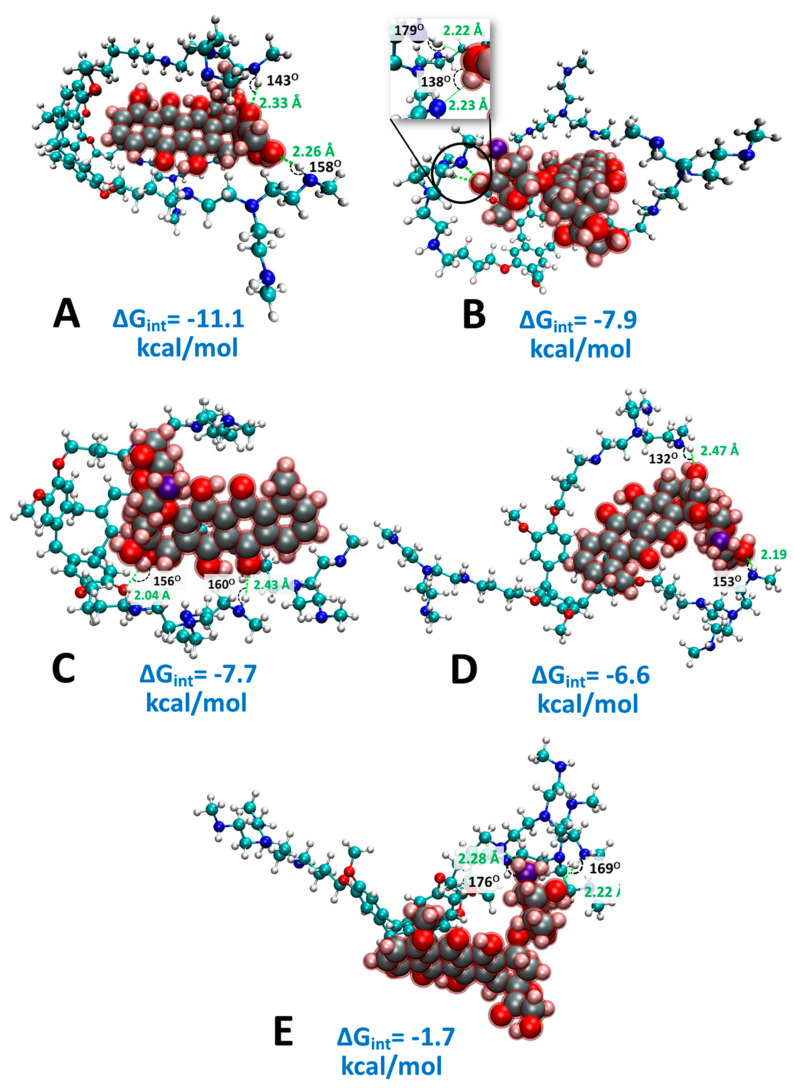
DFT optimized structure of the most stable configurations of doxorubicin (highlighted in light red) bound to the copolymer unit (cyan is carbon, white is hydrogen, red is oxygen, and blue is nitrogen). Hydrogen bonds between the polymer and doxorubicin are shown in green dashed lines, and the bond length in shown in green text, while the hydrogen bond angles are marked by black dashed arc and black text. (**A**–**E**) are some of the possible configurations considered in this paper with the corresponding polymer-doxorubicin free energy of interaction (ΔG_int_) written in blue.

After optimizing the isolated structure of the isolated copolymer unit and Doxorubicin (Figure 7), we designed several configurations of the polymer–Doxorubicin complexes based on experimental results and optimized them using DFT. Among the configurations we examined, the one shown in Figure 8A was the most stable based on its free energy of interaction ΔG_int_ (−11.1 kcal/mol). To understand the interactions that stabilize the polymer–Doxorubicin complex, we compared the configuration in Figure 8A with the other less stable configurations starting with Figure 8B. In both Figure 8A,B, two hydrogen bonds were found, and the configuration in Figure 8A produced two N-H…O type of hydrogen bonds that involve the carbonyl oxygen at the carbon 13 (d = 2.33 Å, 143°) and the hydroxyl oxygen at carbon 14 of doxorubicin (d = 2.26 Å, 158°) as labeled Figure 1a. On the other hand, the hydrogen bonds found in the configuration in Figure 8B were stronger (judging from their shorter bond distances and larger bond angles) and involve the alcoholic oxygen in position 4′ of Doxorubicin, as shown Figure 1a: N-H…O (d = 2.22 Å, 179°) and O-H…N (d = 2.23 Å, 138°). Compared to Figure 8A, the configuration in Figure 8B has less favorable ΔG_int_ (−7.9 kcal/mol) despite having more stable H-bonds, which shows that h-bond formation is not the dominant factor that stabilizes the polymer–Doxorubicin interaction. We had also observed that the calculated Grimme’s dispersion energy, which is associated with nonpolar interactions, was stronger for the configuration in Figure 8B (−365 kcal/mol) than the one in Figure 8A (−358 kcal/mol) by about 7 kcal/mol. Thus, the stronger ΔG_int_ for the configuration in Figure 8A could only be explained by the presence of stronger polar interactions between the aromatic rings of **3a** fragment and Doxorubicin. The configuration in Figure 8A allowed for partially positively charged aliphatic hydrogen atoms of the polymer to form interactions with the partially negatively charged oxygen atoms of the drug, as shown in Figure S33. Figure 8C is a configuration where the CTV rings and the aromatic rings of Doxorubicin were oriented away from each other to minimize the π–π interactions between them. This lowered the overall interaction energy (−7.7 kcal/mol), confirming that π–π interactions significantly contribute to the stable binding of the drug to the polymer. Likewise, the configuration shown in Figure 8D where the ethyleneimine groups were “spread open”, to lower the Van der Waals interactions between the drug and the polymer, lowered the ΔG_int_ to −6.6 kcal/mol, confirming the significance of Van der Waals interactions. Finally, Figure 8E presented a configuration whereby the drug was situated at the opposite side of the “cup” structure of CTV results in the weakest ΔG_int_ (−1.7) among the tested configurations. Thus, from the calculations, the most stable binding of the drug to the copolymer involved the interfacing of Doxorubicin to the CTV core through π–π transitional binding and nonpolar interactions. This is followed by the envelopment of the ethyleneimine branches mainly by polar interactions with the alkyl chains while the formation of supporting H-bonds with the amino groups further stabilize the bonding of the drug in the binding pocket. In turn, this had formed the basis of our current chemical assembly for the supramolecular interaction with Doxorubicin. We further calculated the effect of solvation on the overall ΔG_int_ using the implicit solvent models by computing our most stable configuration, as shown in Figure 8A. Table S1 shows that the inclusion of solvent leads to reduced interaction energies due to stabilization of the isolated Doxorubicin and polymer molecules. Furthermore, the binding of the drug to the polymer is less preferred in a less polar solvent such as methanol compared to in water. This implies that the nonpolar interactions (e.g., π–π and London dispersion forces) that are amplified in polar solvents are the major interactions that bind the drug to the polymer.

## 3. Materials and Methods

### 3.1. Materials

Vanillyl alcohol, 1,4-Dibromobutane, Scandium triflate Sc(OTf)_3_, branched Polyethyleneimine PEI from Sigma Aldrich (St. Louis, MO, USA) (average Mw 25,000 by LS, average Mn ~10,000 by GPC, branched), Acetone, and Acetonitrile CH_3_CN were acquired from commercial sources. ^1^H and ^13^C NMR spectra were recorded for solutions in CDCl_3_ and DMSO-*d*_6_ on a Bruker Avance III 400 MHz BBFO Probe with the solvent residual proton signal. Flash column chromatography was performed using Silicycle Silica gel 60 (7– 230 Mesh, pH 7). UV-vis absorption spectra of all samples in MeOH, DMSO, and H_2_O were acquired using Thermo Scientific (Waltham, MA, USA) Multiskan G0 microplate and cuvette spectrophotometer and DU 800 spectrophotometer in the range of 200–1000 nm, respectively. The IR spectra were recorded on a KBr pellets within the range 4000–400 cm^−1^ with a Perkin-Elmer (Waltham, MA, USA) Spectrum Two System FTIR spectrometer. 

### 3.2. Synthetic Procedures

**[4-(2-bromobutoxy)-3-methoxyphenyl]methanol (1a):** K_2_CO_3_ (8.97 g, 65 mmol, 5 equiv) was added to a solution of 3-methoxy-4-hydroxybenzylalcohol (2 g, 13 mmol, 1 equiv) in Acetone (24 mL) under argon atmosphere. After stirring at room temperature for 30 min, 1,4-Dibromobutane (15.5 mL, 130 mmol, 10 equiv) was added in one portion, and the resulting mixture was heated overnight at 55 °C under argon atmosphere. The mixture was poured into water (160 mL) and extracted with ethyl acetate (3 × 80 mL). After evaporation of the organic phase, the crude extract was obtained as a yellow oil. Silica gel chromatography, using ethyl acetate/hexane 4:6 as eluent afforded a white solid (2.42 g, 8.40 mmol, 64 %). ^1^H-NMR (CDCl_3_, 400 MHz, 25 °C): δ 6.92 (m, 1H), 6.86 (m, 1H), 4.62 (m, 2 H), 4.05 (t, *J* = 6.3 Hz, 2H), 3.88 (s, 3H), 3.50 (t, *J* = 6.35 Hz, 2H), 2.07 (m, 2H), 2.00 (m, 2H). ^13^C-NMR (CDCl_3_, 100.61 MHz, 25 °C): δ 149.54, 147.86, 133.97, 131.22, 119.89, 112.54, 110.46, 68.13, 65.24, 56.28, 33.46, 29.43, 27.81.

**2,7,12-Tris-(2-bromobutoxy)-3,8,13-trimethoxy-10,15-dihydro-2H-tribenzo[a,d,g]cyclononene (2a):** [4-(2-bromobutoxy)-3-methoxyphenyl]methanol (2.22 g, 7.70 mmol, 1.0 equiv) and Sc(OTf)_3_ (0.114 g, 0.231 mmol, 0.030 equiv) were dissolved in CH_3_CN dry (50 mL) under nitrogen atmosphere and heated at 92 °C for 2 days with stirring. The solvent was evaporated, and the reaction mixture was dissolved in ethyl acetate (100 mL) and extracted with water (2 × 200 mL). After evaporation of the organic phase, the crude was obtained as a yellow oil. Silica gel chromatography, using ethyl acetate/hexane 1:4 as eluent afforded the pure product as a white solid (983 mg, 1.21 mmol, 47%). ^1^H-NMR (CDCl_3_, 400 MHz, 25 °C): δ 6.85 (m, 6H), 4.76 (d, *J* = 14 Hz, 3H), 4.03 (m, 2H), 3.84 (s, 9H), 3.55 (d, *J* = 14 Hz, 3H), 3.49 (td, *J1* = 6.24 Hz, J2 = 2.36 Hz, 6H), 2.07 (q, *J* = 5.84, 6H), 1.96 (q, *J* = 5.84, 6H). ^13^C-NMR (CDCl_3_, 100.61 MHz, 25 °C): δ 148.37, 147.06, 132.40, 131.93, 115.53, 113.85, 68.37, 56.32, 36.50, 33.54, 29.40, 27.87. MALDI-TOF mass [M]^+^ calc. 810.0766, exp. 810.0761, [M + Na]^+^ calc. 833.0658, exp. 833.0658.

**Cross-linked polymer (3a).** Branched PEI (200 mg) was dissolved in 5 mL of dry DMSO. A solution of 2,7,12-Tris-(2-bromobuthoxy)-3,8,13-trimethoxy-10,15-dihydro-2H-tribenzo[a,d,g]cyclononene **2a** (200 mg) in 3 mL of dry DMSO was added by syringe. The reaction was performed at 75 °C for 3 days. The solution was transferred in a dialysis bag 3000 Da Mw cut off, which was immersed in methanol for three days under magnetic stirring. The solution in the bag was evaporated, and 414 mg of yellow gel was collected.

### 3.3. Computational Analysis

All density functional theory calculations (DFT) were performed using the Gaussian16 package [70]. For simplicity, the cross-linked polymer **3a** was reduced to a fragment composed of a CTV modified with three single ethyleneimine branches (Figure 8). The 3D models of the Doxyrubicin molecule, cross-linked polymer fragment, and various configurations of their complexes were built using Avogadro [71], where they were preoptimized using MMFF [72]. Then, MMFF preoptimized models were optimized in vacuo at the B3LYP/6-31g level of theory [73]. To properly account for weak noncovalent interactions, energies were corrected using Grimme’s dispersion correction, D3(BJ) [74,75,76], and basis set superposition errors were corrected using a geometric counterpoise scheme [77] using the gCP-D3 Webservice [78]. We confirmed that the dispersion energies calculated using Gaussian16 package and the gCP-D3 Webservice for our systems are consistent (Table S2). Frequency analysis was done to confirm that the optimized structures correspond to a true minimum in the potential energy surface and to calculate the Gibb’s free energy at 298 K. The respective free energy of interaction (ΔG_int_) for different polymer–doxyrubicin configurations was calculated as follows:

(1) $$\Delta G_{\mathrm{int}}=\mathrm{GDox}:P-\mathrm{GDox}-\mathrm{GP}$$

where GDox:P, GDox, and GP are the calculated Gibbs free energies of the polymer–doxorubicin complex, the isolated polymer unit, and the isolated Doxorubicin molecule, respectively. Visualization of the optimized structures was done using VMD program [79]. The implicit solvent effects were incorporated using the polarizable continuum model (PCM) by setting dielectric constants of ε = 78.3553 and ε = 32.613 for water and methanol, respectively.

## 4. Conclusions

Doxorubicin is a highly complex drug molecule that comprises a wide range of different functional moieties, such as aromatic rings, acidic protons, and aliphatic chains. Considering the fact that the supramolecular interaction of drugs is now an important aspect for modern drug delivery systems, it is therefore important to harness all of these groups so that they play a collective role during the coordination event. In this work, a heterogeneous and flexible hosting system for Doxorubicin has been prepared, and the different degrees of interactions had been systematically described. At different levels of the hosting system, it was possible to discern the various coordinative functionalities: for example, –NH– amino groups that can interact with the acidic protons; armed CTV that interacts with Van der Waals forces amplified by a hydrophobic effect; a flexible encapsulating cavity that can adapt its shape for optimizing the coordination. The incorporation of the CTV units in a polyamine had allowed for the coordination of the Doxorubicin while avoiding ionizing interactions with the –NH– groups that could potentially destabilize the drug or alter its biological activity. The DFT calculations proved the intrinsic stability of the coordination of Doxorubicin inside the CTV unit of **3a**, without and with considering the effect of medium where the compounds are mixed. The DFT calculation further provided many important details pertaining to how the spatial orientation of doxorubicin may exist within CTV core and how the overall coordination could exist between the various levels. The analysis in different solvents as well as the energy simulation in vacuum had revealed that all of the functionalities of **3a** had worked in tandem to help orientate and coordinate the drug molecule and functional groups of Doxorubicin were actively involved during the process. It has been demonstrated for the first time that functionalization of the CTV may help to coordinate Doxorubicin and protect it from the interactions with eventual destabilizing agents dispersed in the solvents. In turn, the design of **3a** could be a potential tool to create new biomimicry drug delivery systems, because **3a** exhibits different functionalities for coordination of guest molecules akin to those of the biological systems. It is certainly thought that such a molecular system may subsequently help carry the drug to close proximity of DNA strands due to the intrinsic gene coordination capability of the PEI chains. The work also shows for the first time the unique assembly and documentation of Doxorubicin with a CTV-derived supramolecular carrier, and future work will involve the strategy for the release of Doxorubicin from such system.

## Figures and Tables

**Table 1:** UV-vis absorption maxima of 2.9 × 10^−5^ M Doxorubicin pure solutions and titrated in MeOH, DMSO, and H_2_O.

mg of Titrant ^1^ (Solvent)	λ_1_, λ_2_ (nm)	ε_1_, ε_2_, (M^−1^ cm^−1^)
0 mg (MeOH)	475, 495	18,560, 17,460
5 mg ^2^ of **3a** (MeOH)	475, 495	10,860, 10,840
3 mg ^3^ (of **3a** (MeOH aged 20 days)	500, 535	13,060, 13,330
3 mg ^3^ of PEI (MeOH)	480, 495	17,400, 17,460
0 mg (DMSO)	480	9260
3 mg ^3^ of **3a** (DMSO)	485	10,200
3 mg ^3^ of **3a** (DMSO aged 20 days)	485	10,200
3 mg ^4^ of PEI (DMSO)	500, 595	7330, 3420
0 mg of (H_2_O)	480	13,200
3 mg ^3^ of **3a** (H_2_O)	485, 495	10,860, 10,850
3 mg ^3^ of **3a** (H_2_O aged 20 days)	510	7010
3 mg ^4^ of PEI (H_2_O)	550, 590	10,300, 9100

^1^ The titration has been performed on 1 mL of Doxorubicin solution 2.9 × 10^−5^ M. ^2^ 2.5 × 10^−4^ mmol. ^3^ 1.5 × 10^−4^ mmol. ^4^ 3.0 × 10^−4^ mmol.

**Table 2:** ^1^H-NMR ppm signals of Doxorubicin alone and mixed with **3a** or PEI (solvent).

Protons ^2^	(MeOD)	+3a (MeOD)	+PEI (MeOD)	(DMSO)	+3a (DMSO)	(D_2_O)	+3a (D_2_O)	+3a aged (D_2_O)
-H1	7.99	7.97	7.73	7.85	7.89	7.22	7.20	7.26
-H2	7.86	7.83	7.46	7.85	7.89	7.50	7.48	7.53
-H3	7.60	7.57	7.46	7.61	7.65	7.22	7.20	7.26
-OH 9,4	5.46	5.43	5.35	5.46	5.43	-	-	-
-H1′	5.12	5.12	4.98	5.27	5.27	5.39	5.37	5.41
-H5′	4.29	4.25	4.18	4.19	4.16	4.18	4.18	4.20

^1^ The solutions are prepared by mixing 5 mg of Doxorubicin with 5 mg of titrant where the solutions are titrated. ^2^ The numeration of the protons is referred as reported in Figure 1a.

## Supplementary Materials

The Supporting Information provides all files of the experimental part of the work. Figures S1–S14 concern the ^1^H and ^13^C-NMR, FT-IR, and UV-vis analysis for the characterization of the intermediate and final products of the synthesis **1a** and **2a, 3a**. Figures S15–S22 report the FT-IR and UV-vis spectra for the analysis of the interactions between **3a** and Doxorubicin. Figure S23 reports the UV-vis spectra of Doxorubicin mixed with PEI in H_2_O, Methanol, DMSO. Figures S24–S26 reports the ^1^H-NMR spectra of Doxorubicin in DMSO-*d*_6_, MeOD, D_2_O in the range 1–14 ppm. Figures S27–S28 displays the ^1^H-NMR spectra, in the range 1–14 ppm, of Doxorubicine mixed with **3a** in DMSO, before and after aging for 25 days. Figures S29–S30 report the ^1^H-NMR spectra Doxorubicine mixed with **3a** in MeOD before and after aging for 25 days, in the range 1–14 ppm. Figure S31 reports the ^1^H-NMR spectrum of 3a in MeOH after aging for 25 days. Figure S32 reports the comparison between the spectra of doxorubicin before and after mixing with **3a** in DMSO-d6 and MeOD. Figures S33–S34 report the ^1^H-NMR spectra of Doxorubicin mixed with **3a**, before and after aging for 25 days, in the range 1–14 ppm, in D_2_O. Figure S35 reports the ^1^H-NMR spectrum of **3a** in D_2_O after aging. Figures S36 and S37 report the NOESY and NOE NMR spectra of Doxorubicin mixed with **3a** in D_2_O. Figures S38-S40 reports the ^1^H-NMR spectra of Doxorubicine mixed with PEI in DMSO-*d*_6_, MeOH, D_2_O. Figure S41 illustrates the electrostatic potential map of the most stable Doxorubicin–polymer complex, referring to Figure 8A in the main text, based on Mulliken population analysis via DFT calculations. Table S1 displays the Gibb’s free energy of interaction (ΔGint) of the most stable Doxorubicin–polymer complex in vacuum (in Figure 8A, referring to the main text), water, and methanol.
