# Supplementary material for: Functionalization of Polyethyleneimine with Hollow Cyclotriveratrylene and Its Subsequent Supramolecular Interaction with Doxorubicin
Source: Molecules. 2020 Nov 20;25(22):5455. doi: 10.3390/molecules25225455 (PMC7699908; doi:10.3390/molecules25225455)
Supplement: Supplementary file 1 [file molecules-25-05455-s001.pdf]

Functionalization of Polyethyleneimine with hollow Cyclotriveratrylene and its subsequent Supramolecular interaction with Doxorubicin

Carmine Coluccini[1]*, Yoke Mooi Ng[1], Yves Ira A. Reyes[2], Hsin-Yi Tiffany Chen[2], Yit Lung Khung[3]*

[1] Institute of New Drug Development, China Medical University, No.91 Hsueh-Shih Road, Taichung, 40402, Taiwan

[2] Department of Engineering and System Science, National Tsing Hua University, Hsinchu 30013, Taiwan

[3] Department of Biological Science and Technology, China Medical University, No. 100, Jingmao 1st Rd, Beitun District, Taichung City 406, Taiwan

*Correspondence: carmine.coluccini@mail.cmu.edu.tw, yitlung.khung@mail.cmu.edu.tw

The Supporting Informations provide all files of the experimental part of the work.

Characterization of compound 2a

The figures S1-S3 concerns the ^1H and ^{13}C-NMR analysis in $CDCl_3$ for the characterization of the intermediate **1a**. The figures S4-S7 concerns the ^1H and ^{13}C-NMR , mass spectroscopy analysis in $CDCl_3$ of the compound **2a** included the NOE NMR for analizing the structural isomerism of the product.

Characterization of polymer 3a and precursors.

In figures S8-S10 are reported the ^1H-NMR spectra of the cross-linked polymer **3a** in DMSO-d_6, MeOD, D_2O. In figure S11 is reported the spectrum of PEI in DMSO-d_6. In figure S12 is reported the spectrum of **2a** in DMSO. In figure S13 are reported the UV-vis spectra of **3a**, **2a** and PEI in Methanol, DMSO and Water. In figure S14 are reported the FT-IR spectra of compounds **2a**,**3a** and PEI.

FT-IR and UV-vis characterization of interaction between 3a and Doxorubicin.

In the figure S15 are reported the FT-IR spectra of Doxorubicin, **3a**, PEI in the pure form and mixed each other. In figures S15 are reported the UV spectra of Doxorubicin pure and mixed with **3a**, in Methanol and DMSO, before and after aging. In figure S17 are reported the UV spectra of polymer **3a** after aging in Water and Methanol.. In figure S18 is reported the titration of Doxorubicin with **3a** in Methanol followed with UV-vis spectroscopy and in figure S19 the UV-vis spectra of the solutions after addition of more than 3 mg/mL of 3a and also after aging for 25 days. In figure 20 are reported the UV-vis spectra of the Doxorubicine solutions mixed with 5mg/mL of **3a** in DMSO, before and after aging. In figure S21 is reported the titration of Doxorubicin solution with polymer **3a** in water. In figure S22 is reported the UV-vis spectrum of Doxorubicin in H_2O after aging for 25 days. In figure S23 are reported the UV-vis spectra of Doxorubicin mixed with PEI in H_2O, Methanol, DMSO.

^1H-NMR characterization of interactions between 3a and Doxorubicin.

In figures S24-S26 are reported the ^1H-NMR spectra of Doxorubicin in DMSO-d_6, MeOD, D_2O in the range 1-14 ppm. In figures S27-28 are displayed the ^1H-NMR spectra, in the range 1-14 ppm, of Doxorubicine mixed with **3a** in DMSO, before and after aging for 25 days. In figure S29-S30 are reported the ^1H-NMR spectra Doxorubicine mixed with **3a** in MeOD before and after aging for 25

days, in the range 1-14 ppm. In figure S31 are reported the spectra of **3a** in MeOH after aging for 25 days. In figures S33-S34 are reported the ^{1}H-NMR spectra of Doxorubicin mixed with **3a**, before and after aging for 25 days, in the range 1-14 ppm, in D_2O. In figure S35 is reported the ^{1}H-NMR spectrum of 3a in D2O after aging. In figures S35 and S36 are reported the NOESY-NMR and NOE-NMR analyses respectively of the Doxorubicin mixed with 3a solution in D_2O. In figure S37-S40 are reported the ^{1}H-NMR spectra of Doxorubicine mixed with PEI in DMSO-d_6, MeOH, D_2O.

Computational analysis of the interaction between 3a and Doxorubicin

Figure S41 illustrates the electrostatic potential map of the most stable Doxorubicin-polymer complex, referring to Figure 8A in the main text, based on Mulliken population analysis via DFT calculations. Table S1 displays the Gibb's free energy of interaction (ΔGint) of the most stable Doxorubicin-polymer complex in vacuum (in Figure 8A, referring to the main text), water and methanol.

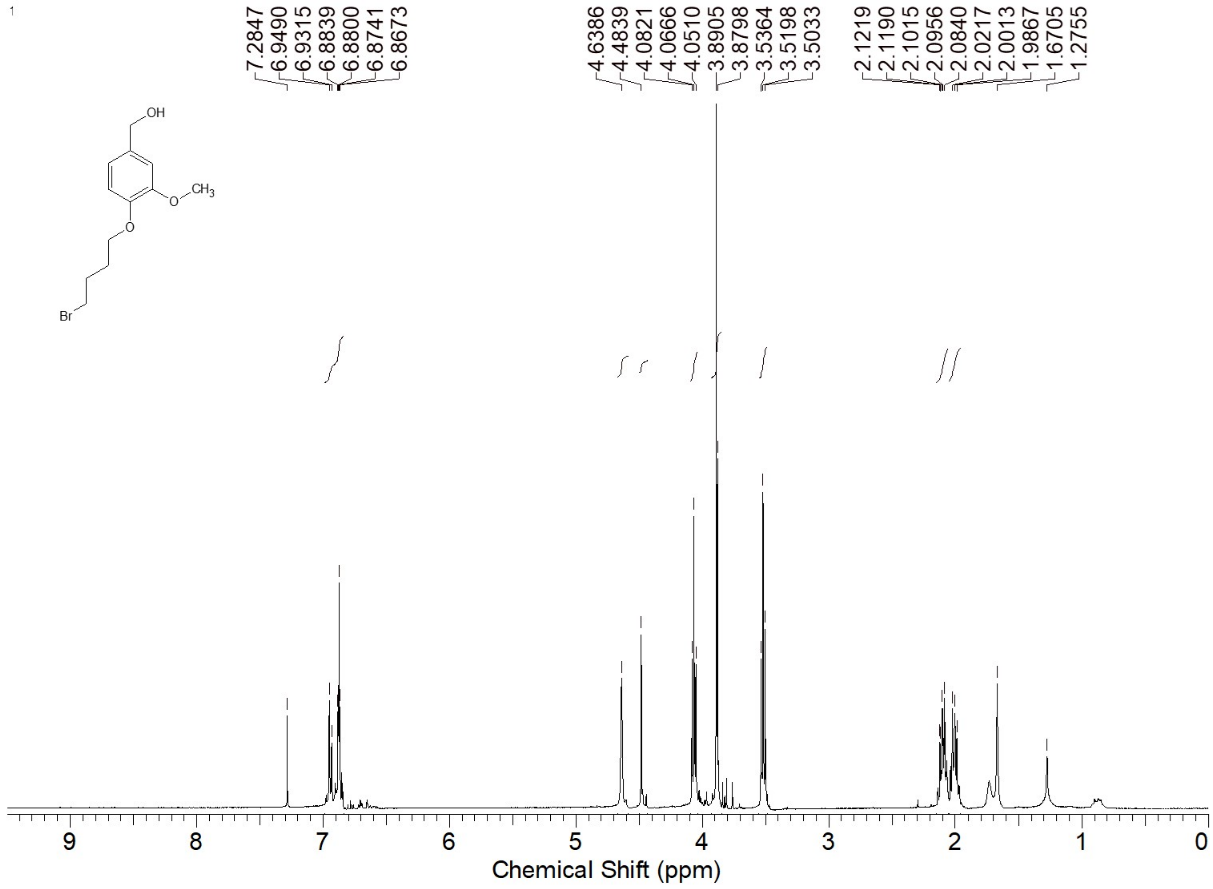

Figure S1. ^{1}H-NMR spectrum in $CDCl_3$ of compound **1a**.

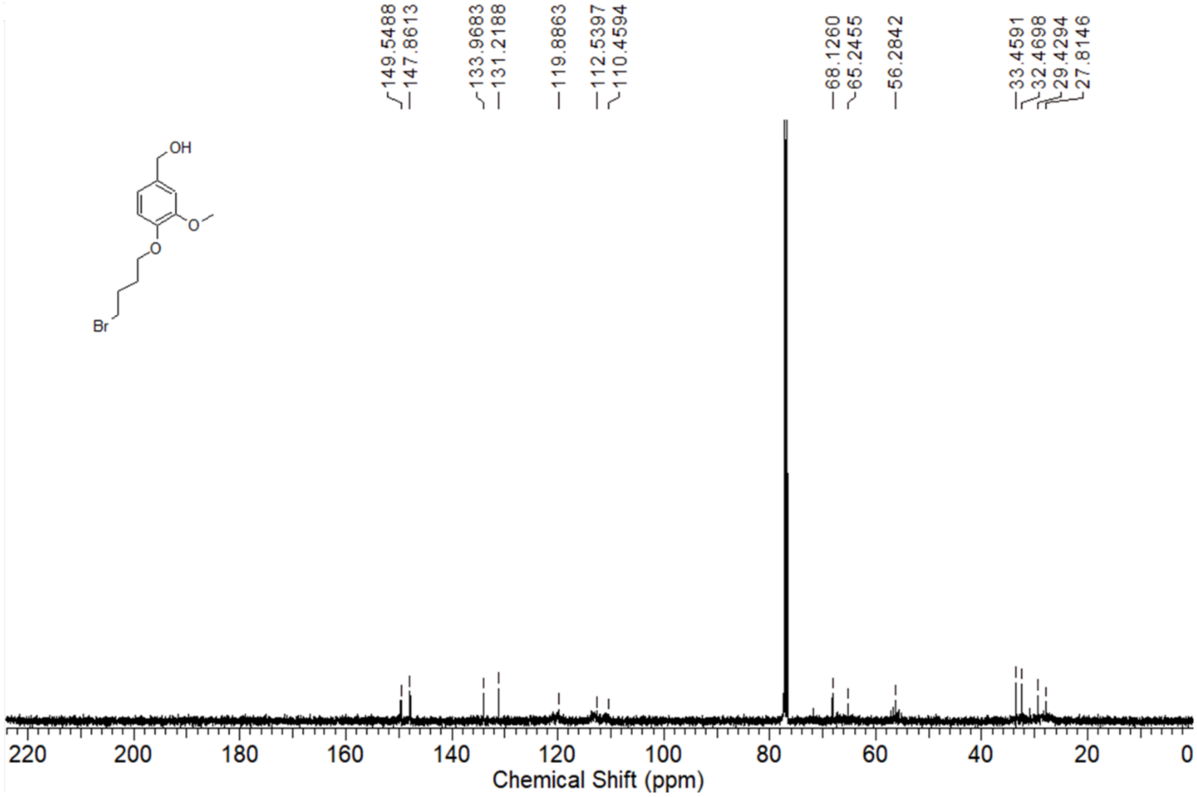

Figure S2. ^{13}C-NMR spectrum in $CDCl_3$ of compound **1a**.

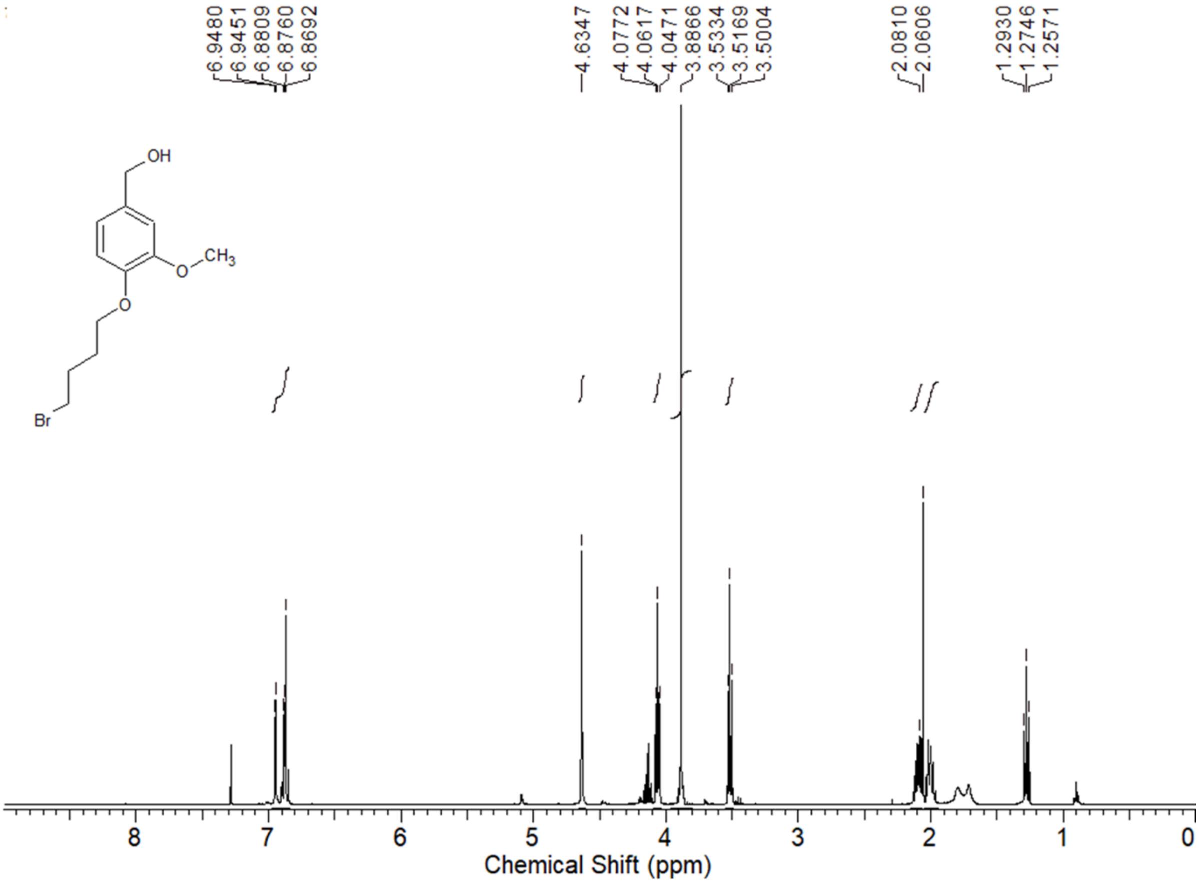

Figure S3. ^{1}H-NMR spectrum in $CDCl_3$ of compound **1a** as pure alcohol.

We retained that the obtained product **1a** is a mixture of alcohol/alkoxide 3:1 because of the presence of two ^{1}H-NMR signals of the –CH_2O- (4.63 and 4.48 ppm in figure S1, which integral ratio is 3:1) and –OMe (3.89 and 3.87 ppm in figure S1). To prove that the signals at 4.48 and 3.87 ppm of figure 1SI refer to the -CH_2O- and –OMe of the alkoxide, respectively, we solubilized 20 mg of the product in ethyl acetate, and we added an aqueous solution of HCl 30 %. After stirring the heterogeneous solution for 1h, and removed the aqueous phase, the organic phase was evaporated and controlled with ^{1}H-NMR. The procedure was repeated several times until the disappearing of the signals at 4.48 and 3.87 ppm, as shown in figure S3. The treatment of product 1a with acid produced the total conversion of alkoxide in alcohol. The time-consuming procedure for the complete conversion induced us to utilize the mixture alcohol/alkoxide 3:1 for the second step of synthesis because they exhibit the same reactivity. The 2nd reaction allowed us to obtain product **2a** with high purity, as shown in figures S4-5.

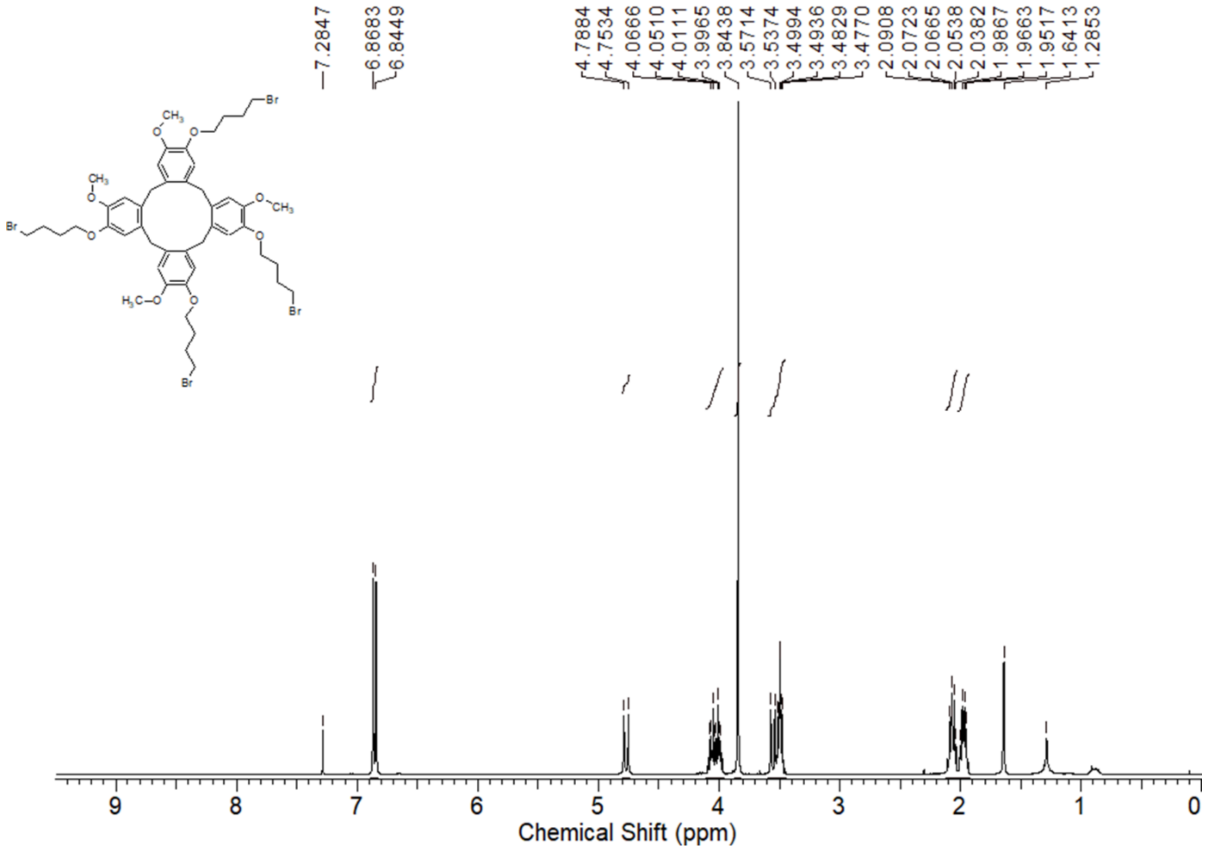

Figure S4. ^{1}H-NMR spectrum in $CDCl_3$ of compound **2a**.

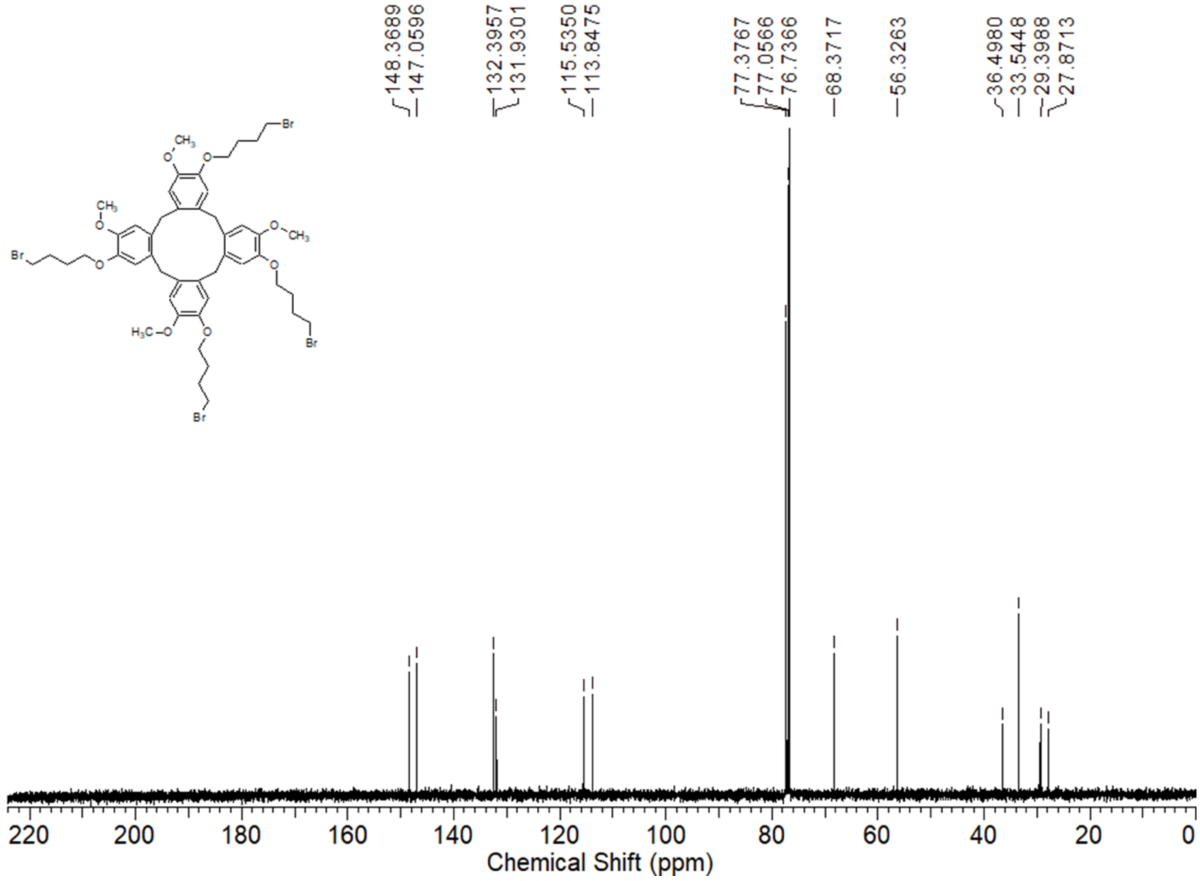

Figure S5. ^{13}C-NMR in $CDCl_3$ of compound **2a**.

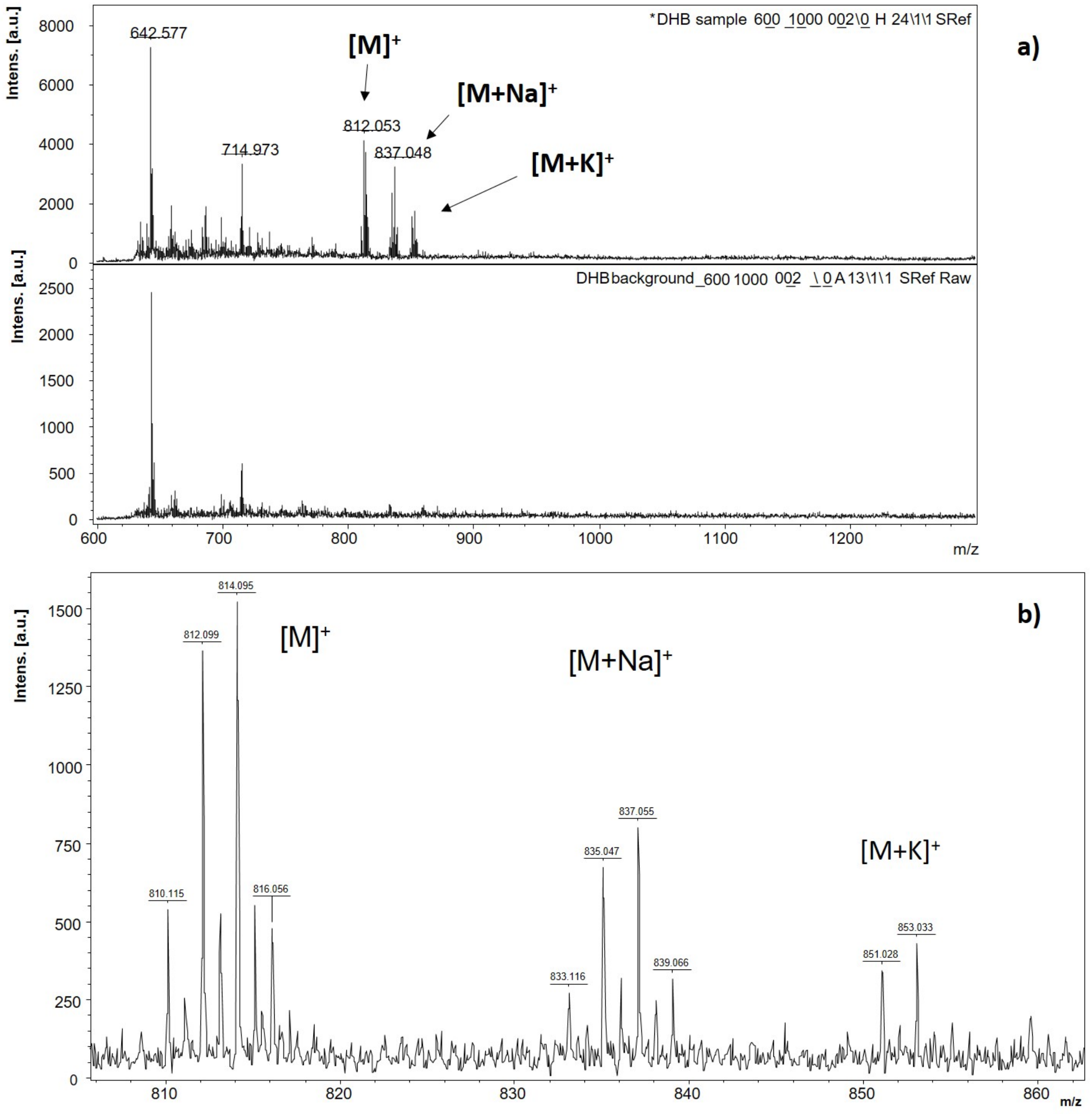

Figure S6. MALDI-TOF MS of compound **2a**. a) In the spectrum detected in the whole range m/z(0-1500), are only visible the peaks around $[M]^+$ (812.73 m/z), $[M+Na]^+$ (837.48 m/z), and $[M+K]^+$ (853.03 m/z). The other signals that are visible in the spectrum are due to the DHB background. b) In the spectrum detected in the range 800-870 m/z it is possible to have more accurate values of m/z for $[M]^+$ and $[M+Na]^+$ that are reported in the experimental section of the manuscript. $[M+K]^+$ is too low intense and it is not possible to detect the accurate value.

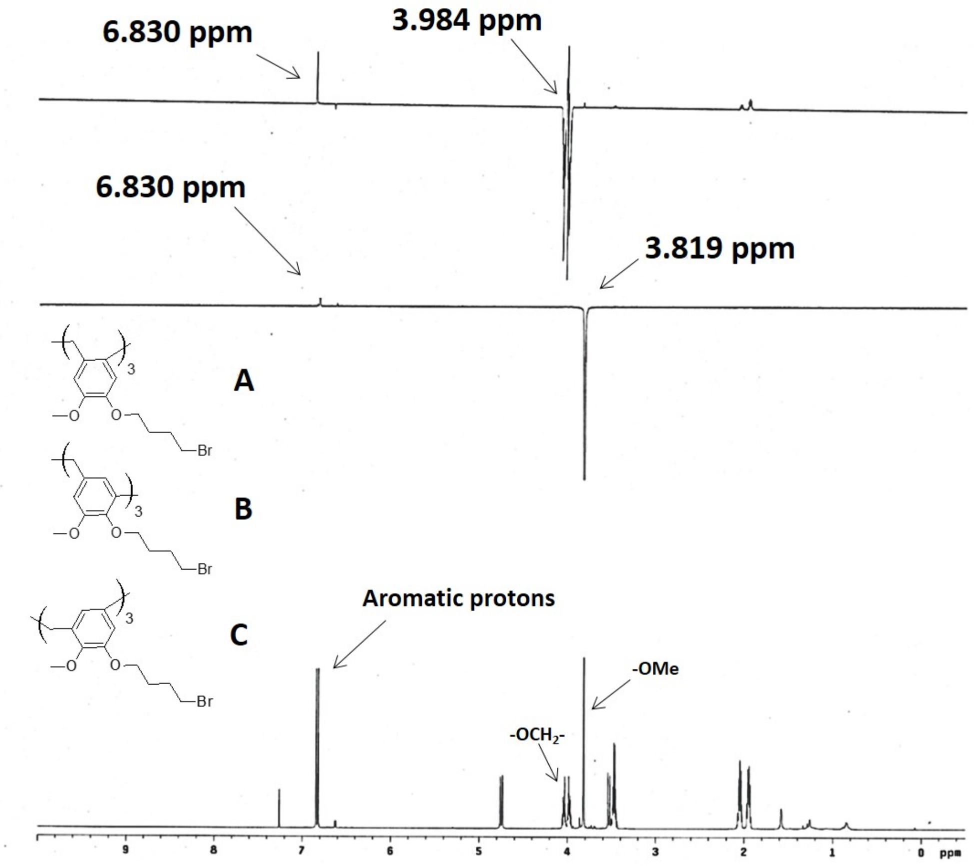

Figure S7. NOE NMR spectra in $CDCl_3$ of compound **2a**. The reaction of the scheme 2 of the manuscript could generate the isomers A, B and C of the figure. In the literature, similar reactions are described and only the isomers like A are generated. Because our compound is not reported in literature we decided to perform the NOE to prove that the A isomer is obtained. The irradiation on the –OCH_2- signal exclude the formation of the isomer B because in this NOE NMR spectrum the aromatic signal is visible (it means that –OCH_2- is close to an aromatic –H). The irradiation on the –OMe signal exclude the formation of the isomer C because in this NOE NMR spectrum the aromatic signal is visible (it means that –OMe is close to an aromatic –H).

.

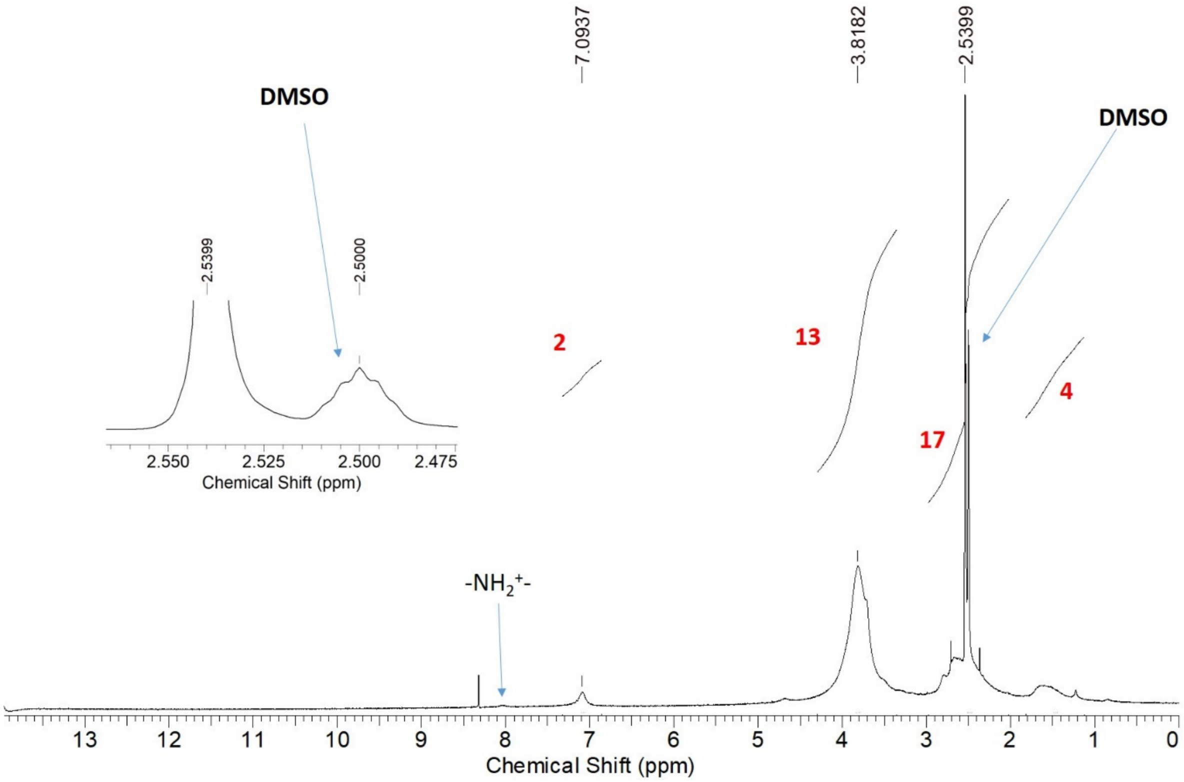

Figure S8. ^{1}H-NMR of **3a** in DMSO-d_6. The red numbers are the values of integrations. The signal around 3.8 ppm should include the signals of –OMe, -OCH_2-, and also the -CH_2Br that not reacted. The signal partially overlaps with H_2O that is always found in the DMSO-d_6. The signal around 2.5 ppm should be the –CH_2N- of PEI and of the CTV aliphatic chain that reacted. The signal is partially overlapped with the signal of DMSO. The signal around 1.5 ppm is the –CH_2CH_2- not linked with heteroatoms of the CTV aliphatic chain, and the integration is 4 as expected by considering that the integration of the two aromatic hydrogen has been fixed to 2.

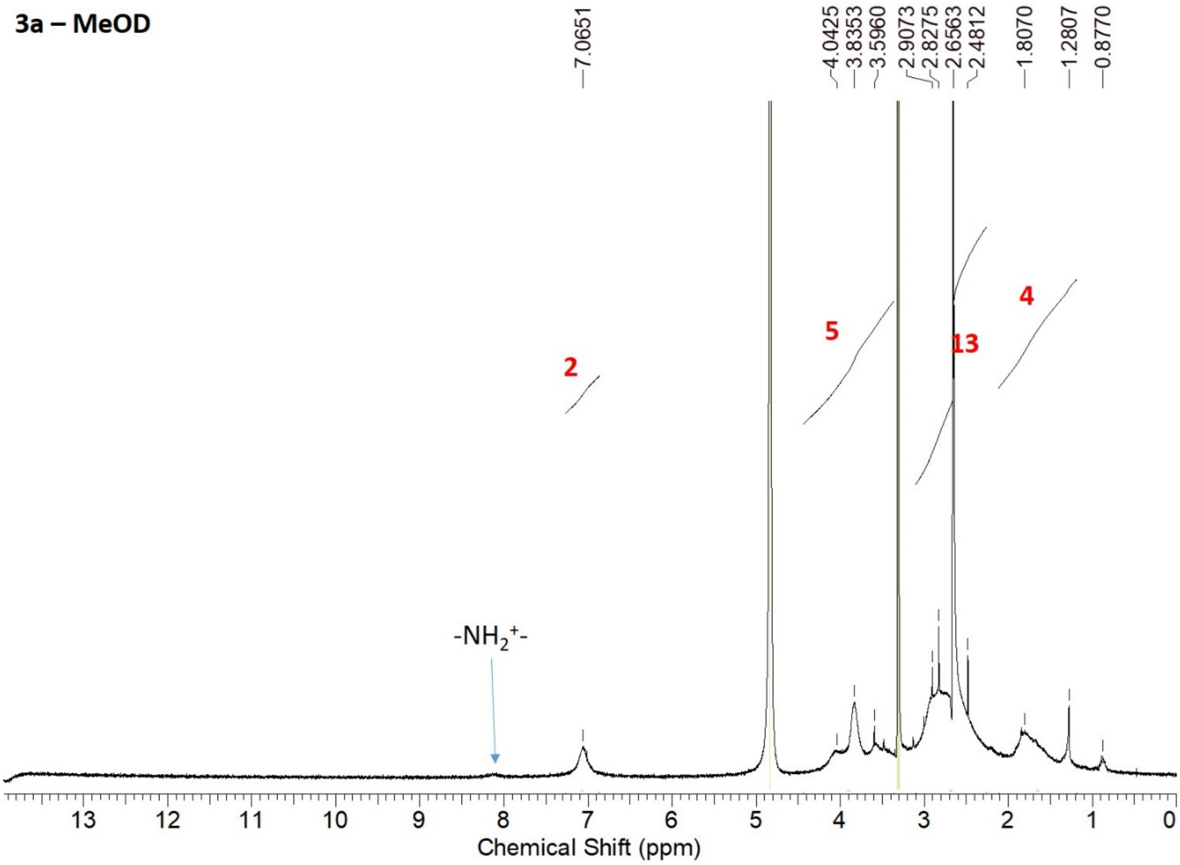

Figure S9. ^{1}H-NMR of **3a** in MeOD. The red numbers are the values of the integrations. The aromatic hydrogens are fixed at 2 and the –OMe, -OCH$_2$- display a value of 5, it means that there are few not reacted -CH$_2$Br. The signal around 2.5 ppm should be the –CH$_2$N- of PEI and of the CTV aliphatic chain that reacted. The signal around 1.5 ppm is the –CH$_2$CH$_2$- not linked with heteroatoms of the CTV aliphatic chain, and the integration is 4.

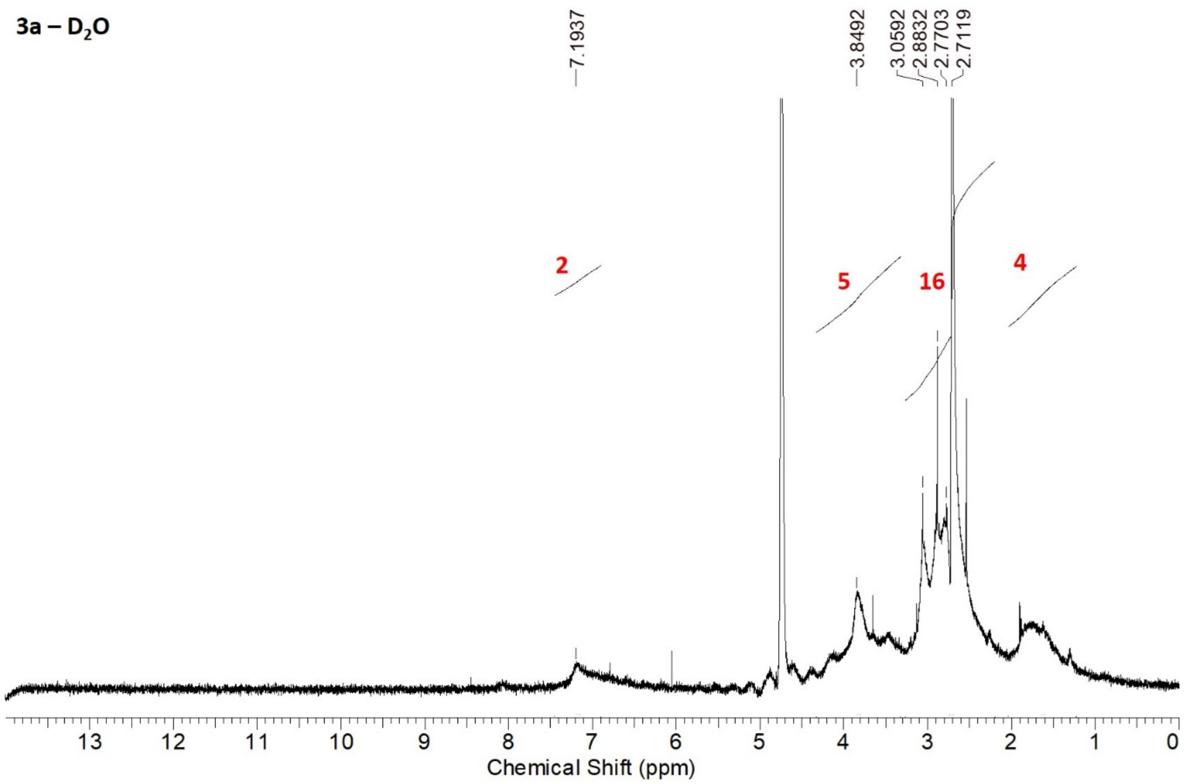

Figure S10. ^{1}H-NMR of **3a** in D_2O. the integrations (the values are the numbers in red) are agree with the values in MeOD and DMSO-d_6.

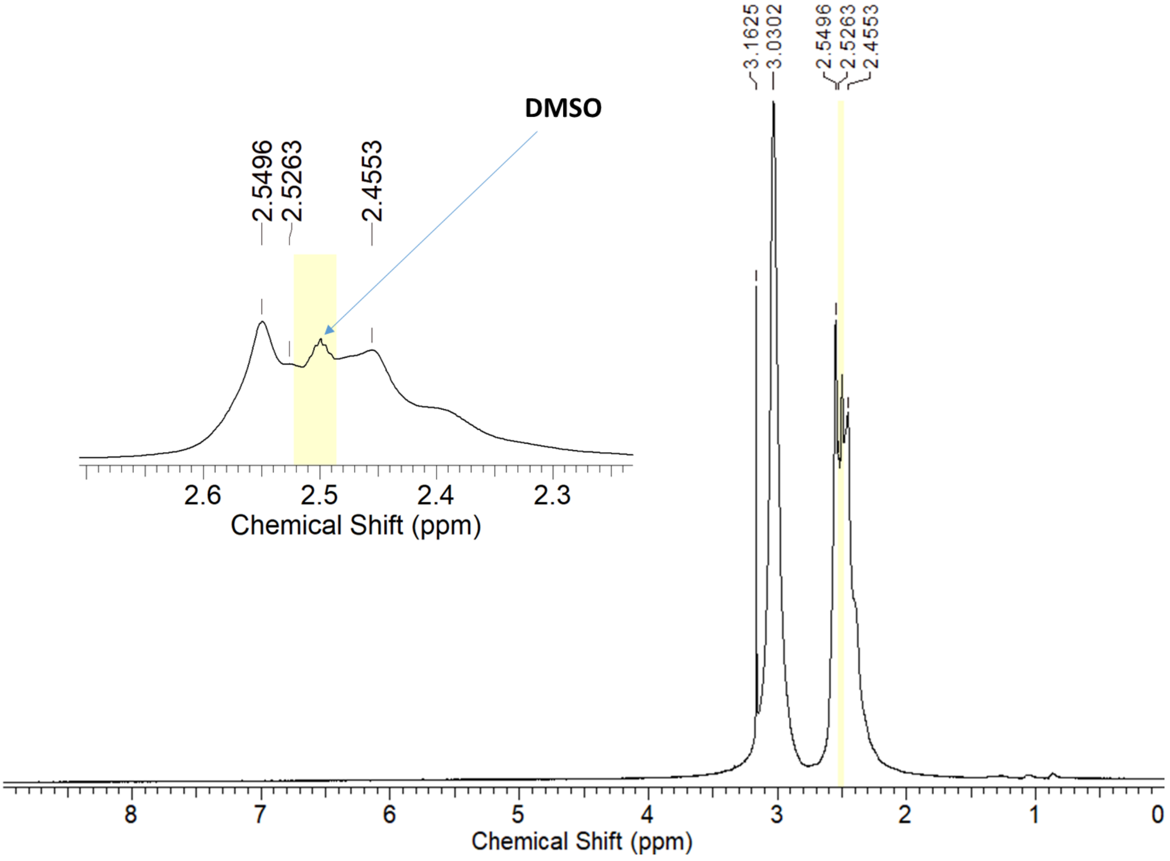

Figure S11. ^{1}H-NMR of PEI.

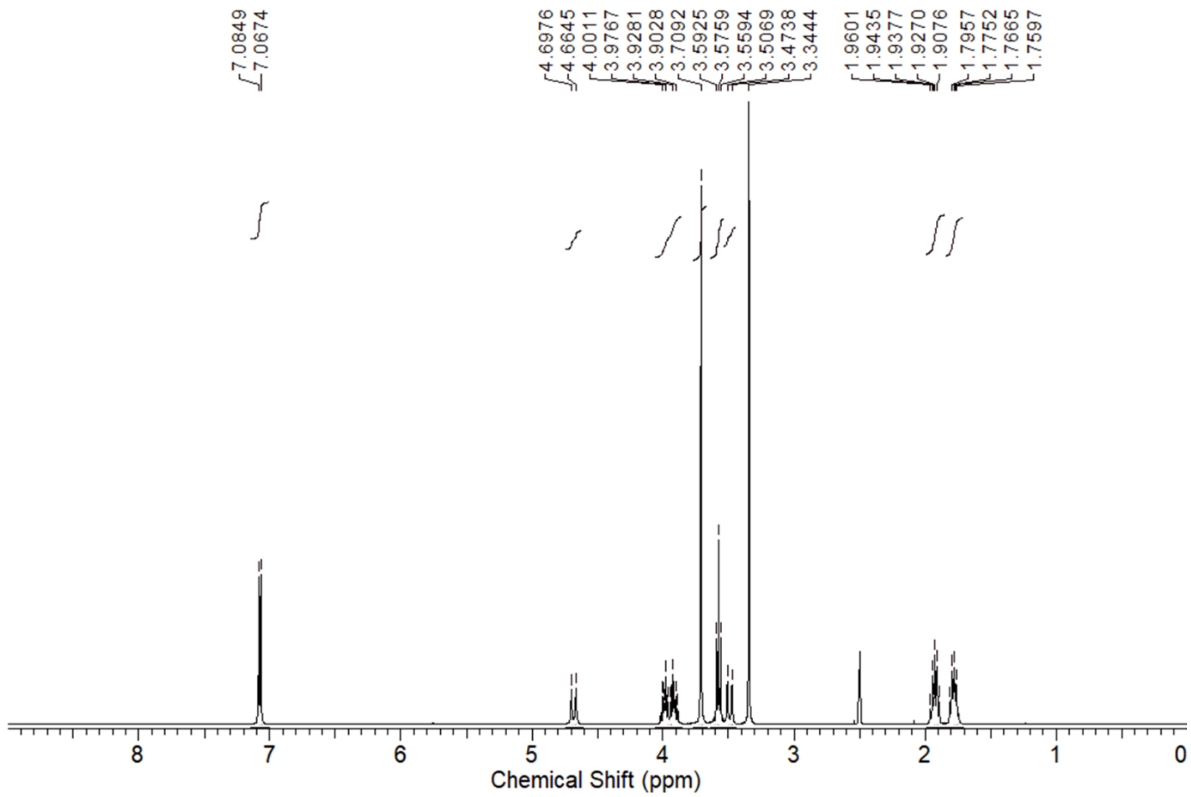

Figure S12. ^{1}H-CNMR of **2a** in DMSO-d_6.

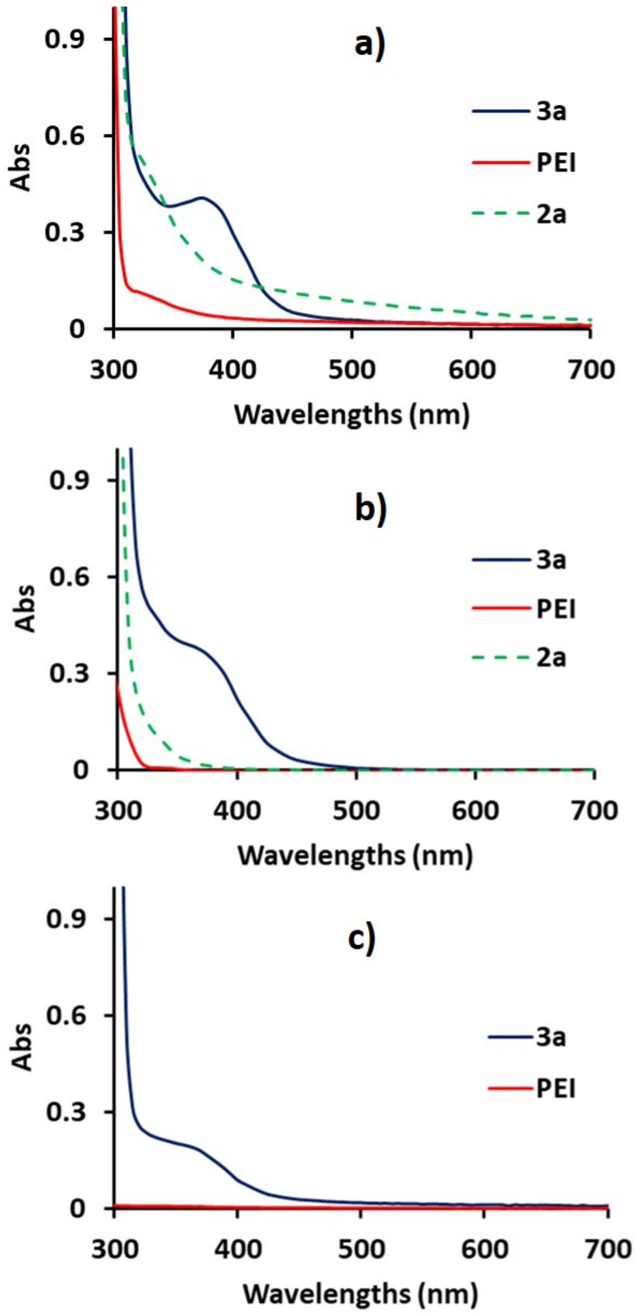

Figure S13. UV-vis spectra in methanol (a), DMSO (b) and water (c) of **3a** (3mg/mL), PEI (3mg/mL), **2a** (3mg/mL)

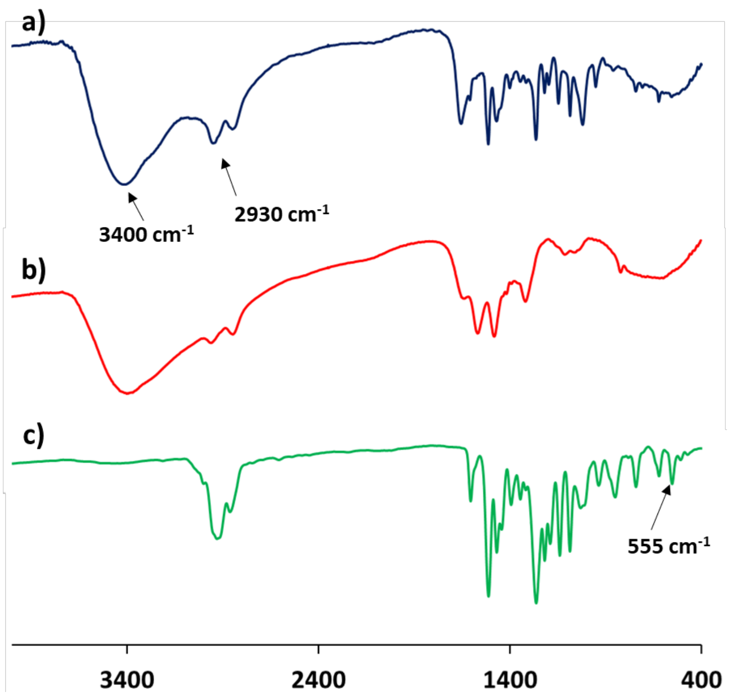

Figure S14. FT-IR spectra: a) **3a**; b) PEI; c) **2a**.

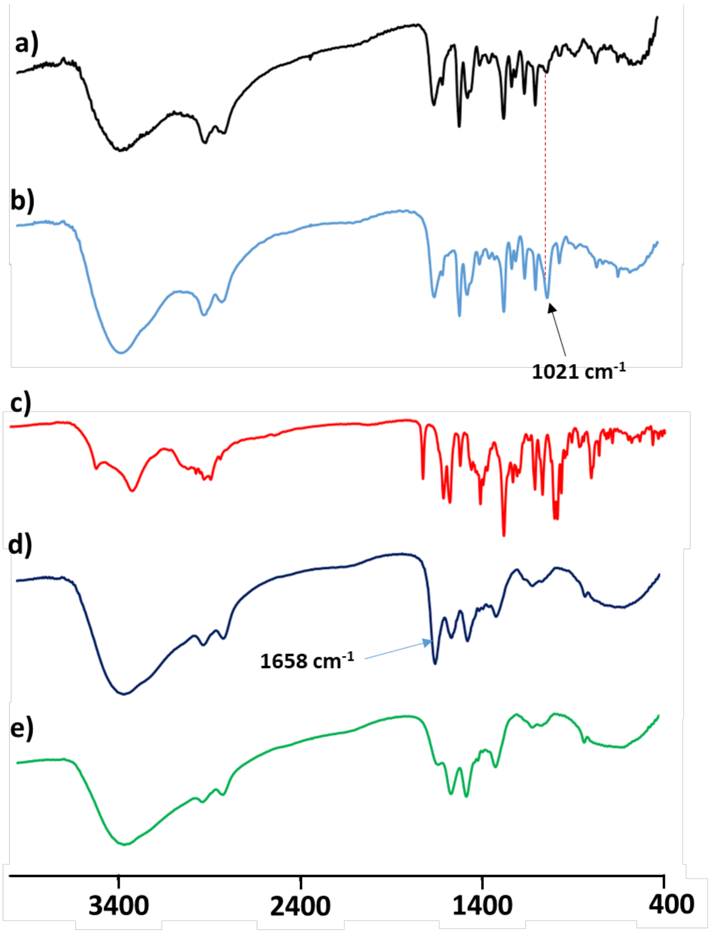

Figure S15. FT-IR spectra 400 – 4000 cm^{-1}: a) **3a** mixed with Doxorubicin; b) **3a**; c) Doxorubicin d) PEI mixed with Doxorubicin; e) PEI.

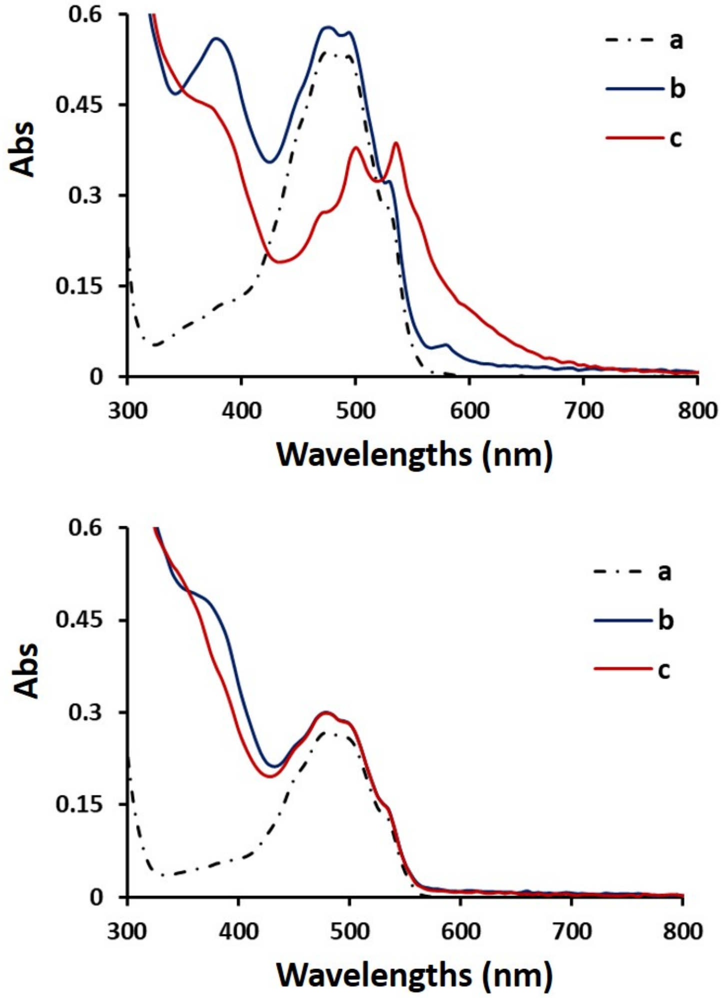

Figure S16. UV-vis spectra of Doxorubicin 2.9*10-5 M mixed with 3a in Methanol (A) and Dimethylsulphoxyde (B). Dash lines a are the solutions of pure Doxorubicin before the addition of 3a., Blue lines b are 1 mL of Doxorubicin solution 2.9*10-5 M after addition of 3 mg of **3a**. Red lines c are 1 mL of Doxorubicin solution 2.9*10-5M after attrition of 3 mg of 3a and after aging for 25 days.

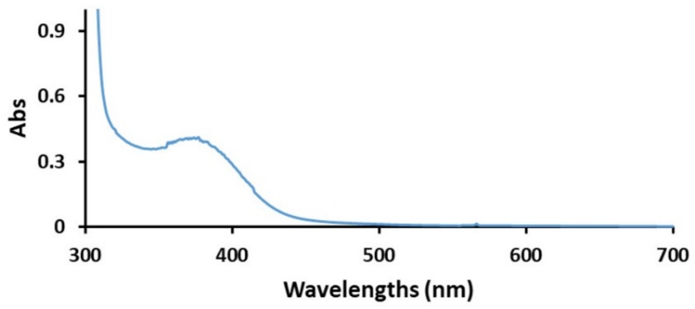

Figure S17. UV-vis spectra of **3a** in Methanol after aging for 60 days.

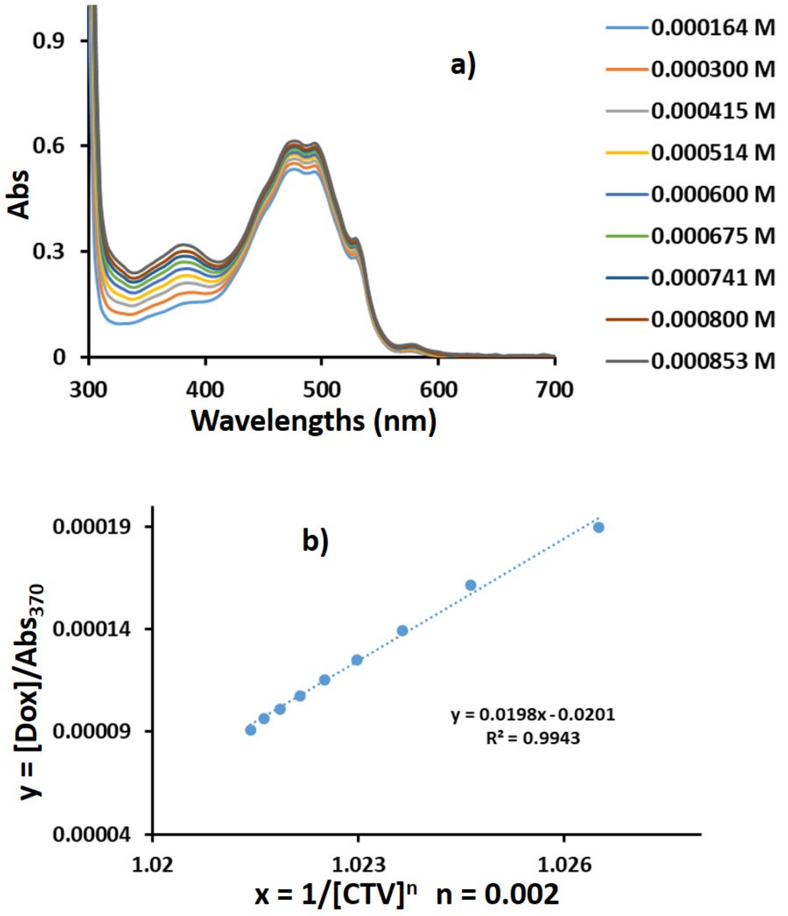

Figure S18. Approach for evaluation of the stoichiometry of interaction between CTV units of 3a and Doxorubicin, based on the UV-vis spectra. a) 1mL of Doxorubicin 2.9*10^{-5} M was titrated with a 1mL solution of Doxorubicin 2.9*10^{-5} M mixed with 3 mg of **3a** (each titration was performed with 0.1 mL). We assumed that in the synthesis of **3a** we had no loss of reagents because we reacted 200 mg of PEI (M_n is 10000, it means that we utilized 0.02 mmol), 200 mg of **2a** (0.25 mmol), and we obtained 400 mg of product, the molecular weight of **3a** is around 20000 g/mol (400 mg/ 0.02 mmol) and each mol of **3a** contain 12.5 mol of CTV units (0.25 / 0.02). 3mg/mol of **3a** is a 1.5*10^{-4} M solution and 0.0018 M if referred to CTV units for mL. Every mL of titrating solution contain 0.00018 mmol of CTV. The legend of the figure is the concentration of CTV for each mL of titrating solution that we added to the solution of pure Doxorubicin. b) If [CTV] and [Dox] are respectively the concentrations of CTV and Doxorubicin, Dox interacts with n*CTV for obtaining the complex Dox$(CTV)_n$, with Keq as equilibrium constant. If Abs is the absorbance variation during the titrations, we can write the following relationships:

$$\mathrm{Keq} = \frac{[\mathrm{Dox(CTV)_n}]}{[\mathrm{CTV}]^n\,[\mathrm{Dox}]}$$

$$\mathrm{Keq} \propto \frac{\mathrm{Abs}}{[CTV]^n[Dox]} \Rightarrow \frac{[\mathrm{Dox}]}{\mathrm{Abs}} \propto \frac{1}{\mathrm{K_{eq}}[\mathrm{CTV}]^n}$$

The function [Dox]/Abs vs 1/$[CTV]^n$ is a straight line and the value of n that fits better with the function represents the stoichiometry of the interaction between Doxorubicin and CTV. If we consider the Abs of the maximum at 370 nm, the best value of n is 0.002 and the constant is 5*10^2.

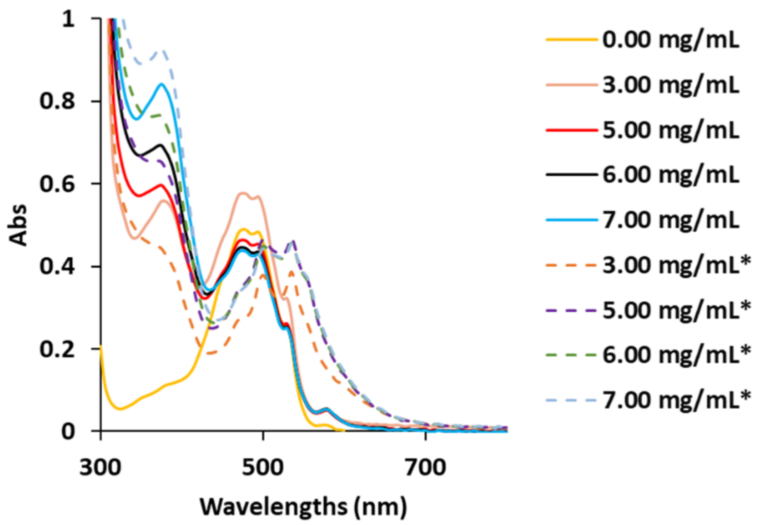

Figure S19. UV-vis spectra changes after aging for 25 days of 1mL of Doxorubicin 2.9*10^{-5} M titrated with **3a**. Dashed lines refer to the curves of the aged solution (in the legend they are marked with *.

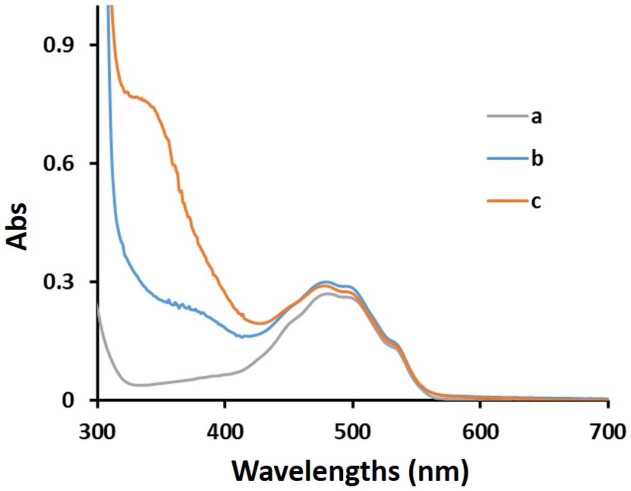

Figure S20. UV-vis spectra in DMSO: a) Doxorubicin 2.9*10^{-5} M; b) 1mL of Doxorubicin 2.9*10^{-5} M titrated with 5 mg of 3a; c) 1mL of Doxorubicin 2.9*10^{-5} M titrated with 5 mg of 3a after aging for 25 days.

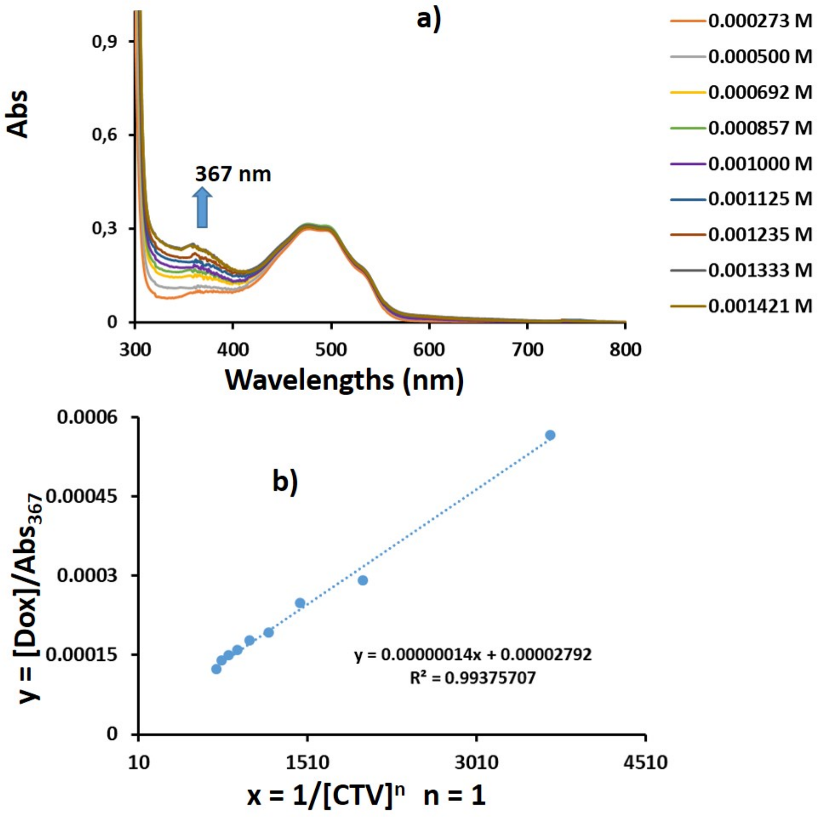

Figure S21. UV-vis titration of Doxorubicin in Water. a) 1mL of Doxorubicin 0.000029 M solution was titrated with 1 mL of a Doxorubicin 0.000029 M solution mixed with 5 mg of **3a**. Every titration was performed with 0.1 mL of titrating solution, the molecular weight of **3a** and the number of CTV units for each mol of polymer was evaluated as in figure S16. The maximum at 367 nm emerges during the titration and should be due to the formation of the complex $Dox(CTV)_n$. The legend reports the concentrations of CTV units that are incorporated in **3a** after every titration. b) The relationship between $[Dox]/Abs_{367}$ and $1/[CTV]^n$ was evaluated as in figure S16 and the value of n that fits better with a linear function is 1. The constant is $7*10^5$.

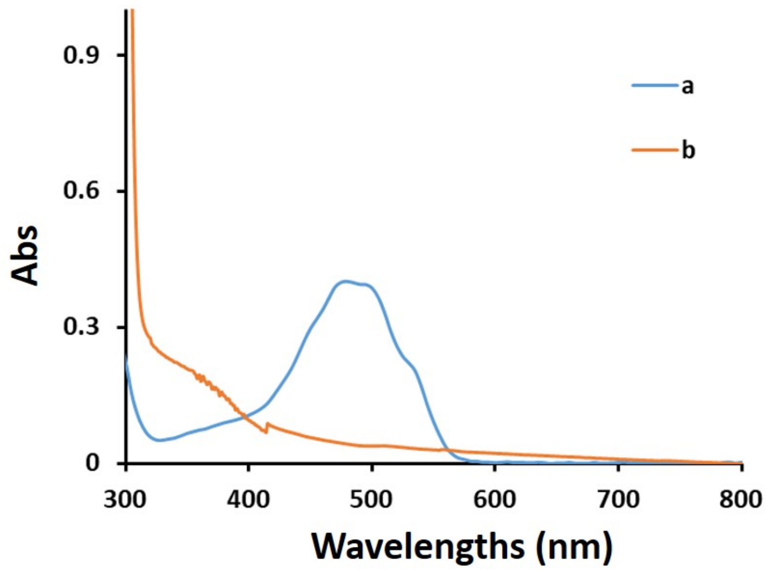

Figure S22. a) UV-vis of Doxorubicin 2.9*10[-5] M in Water aged 25 days. b) 3a in Water, 3mg/mL, aged 60 days.

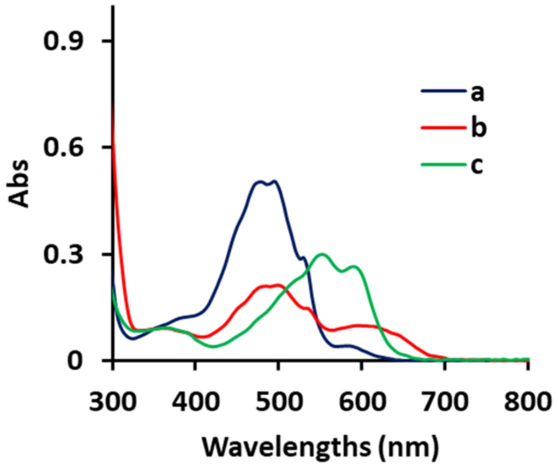

Figure S23. Titration of 1mL of Doxorubicin 2.9*10[-5] M with 3mg of PEI: a) MeOH solution; b) DMSO; c) H_2O.

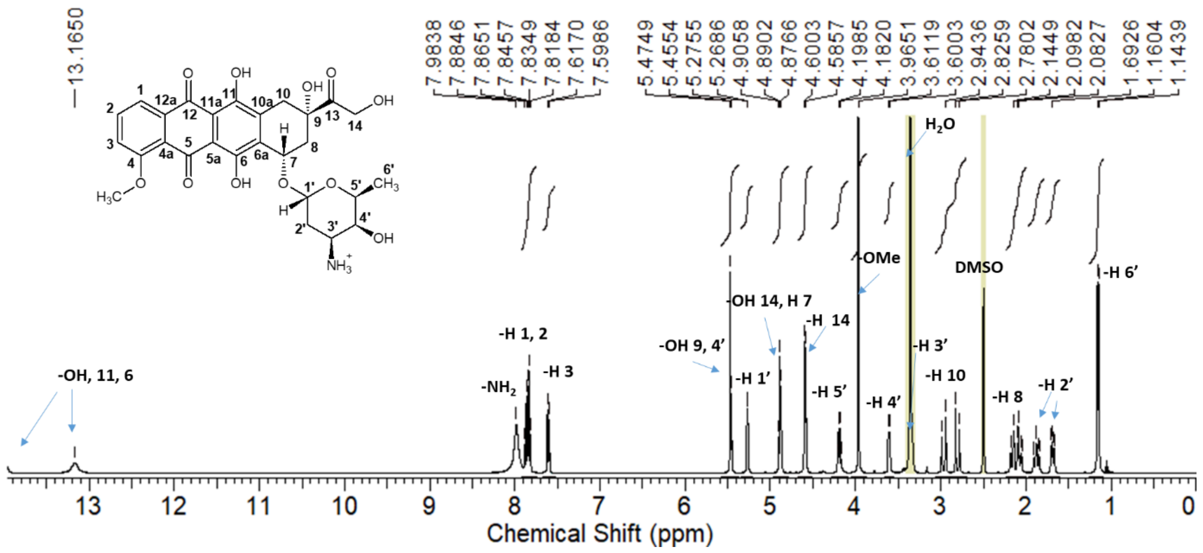

Figure S24. ^{1}H-CNMR of Doxorubicin in DMSO-*d*6.

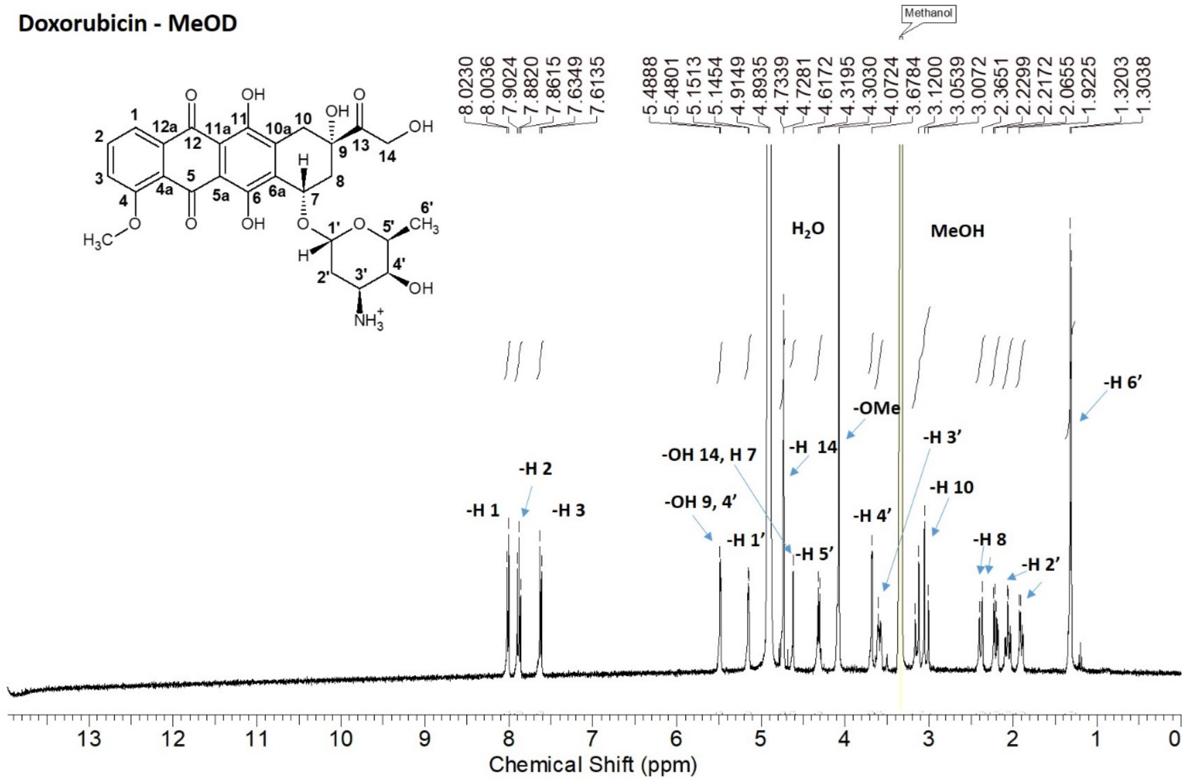

Figure S25. ^{1}H-NMR of Doxorubicin in MeOD.

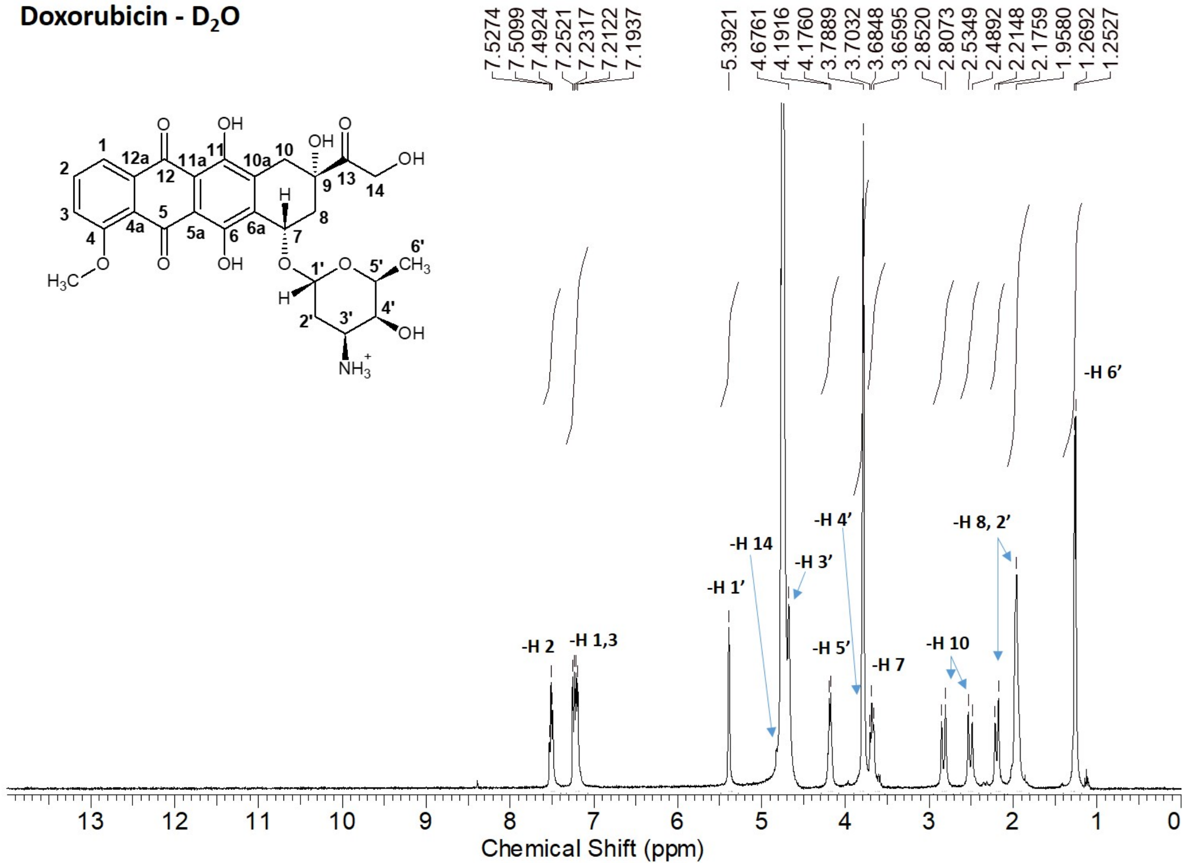

Figure S26. ^{1}H-NMR of Doxorubicin in D_2O.

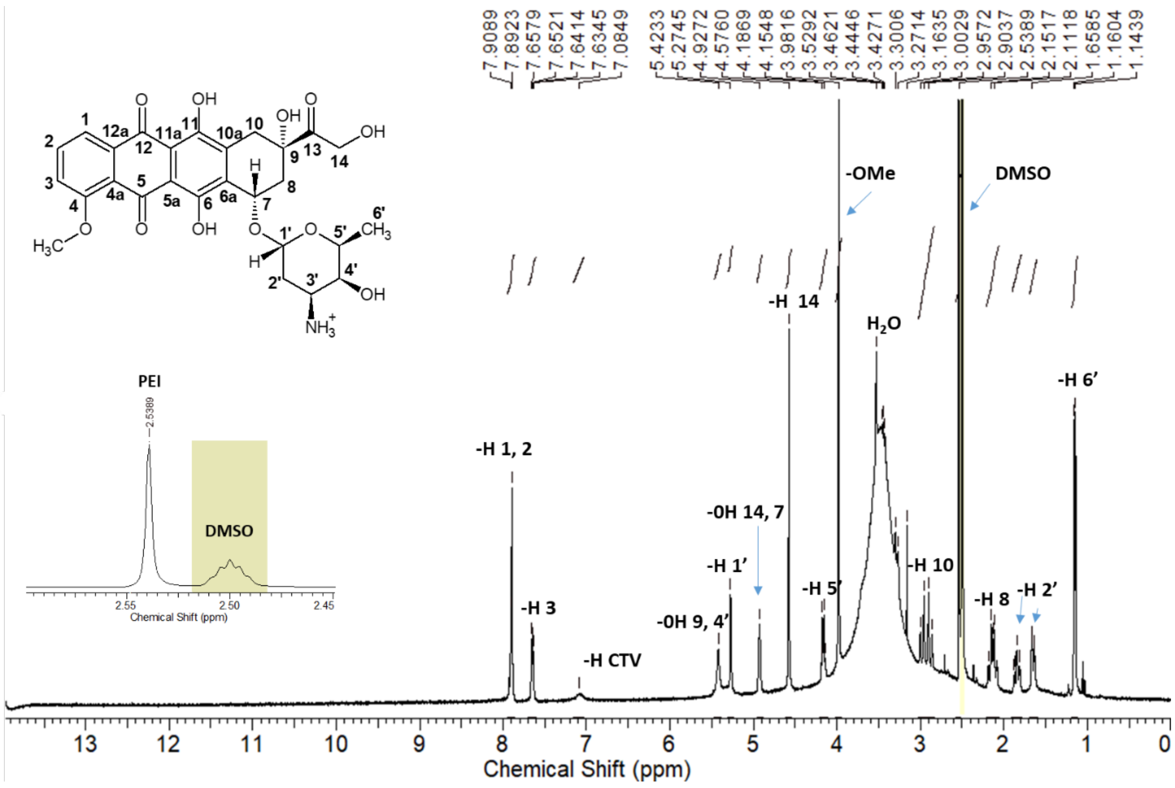

Figure S27. ^{1}H-NMR of Doxorubicin mixed with **3a** in DMSO-*d*6.

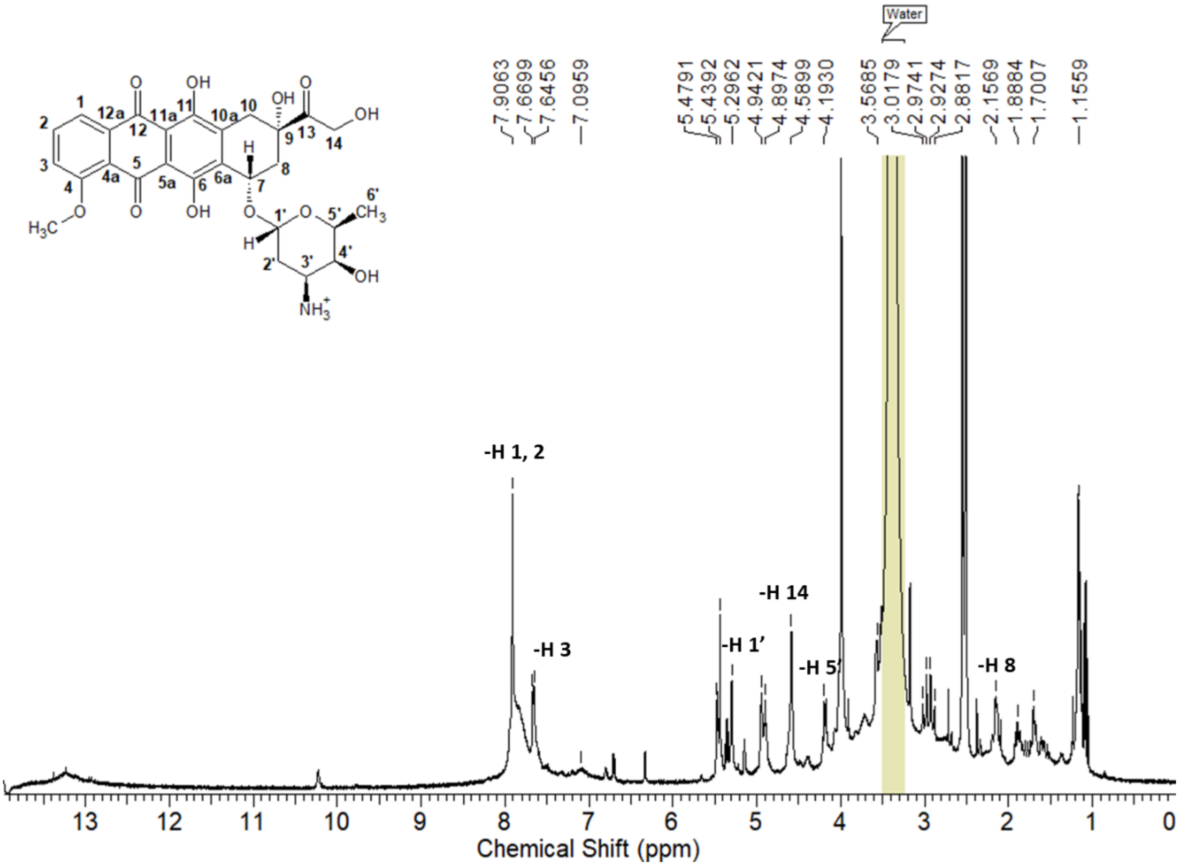

Figure S28. ^{1}H-NMR of Doxorubicin mixed with **3a** in DMSO-*d*6, after aging for 20 days.

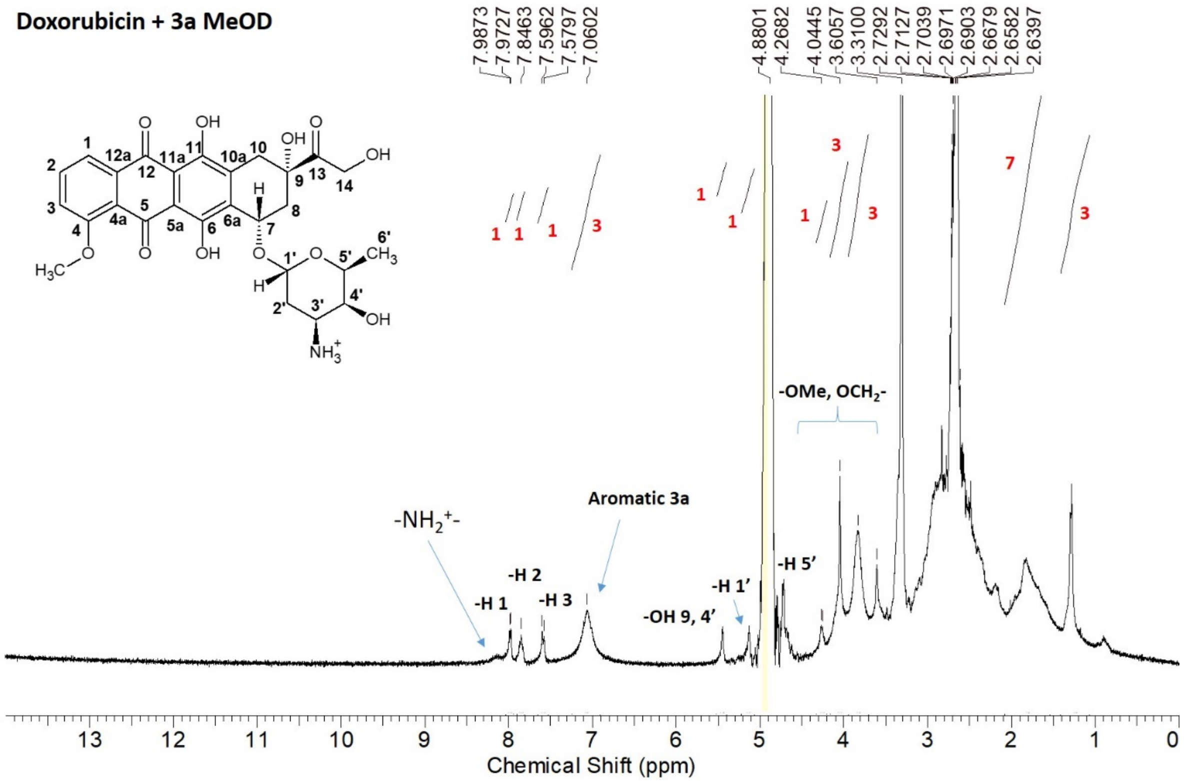

Figure S29. ^{1}H-NMR of Doxorubicin mixed with **3a** in MeOD.

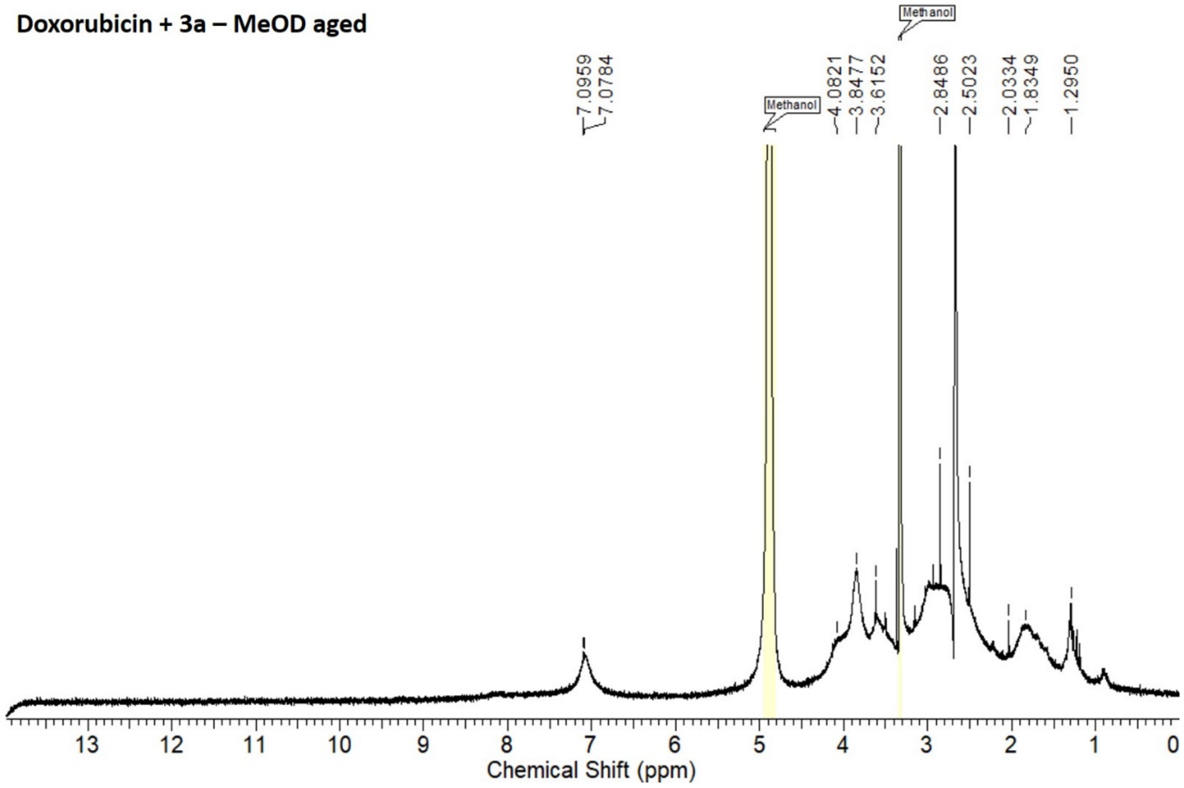

Figure S30. ^{1}H-NMR of Doxorubicin mixed with **3a** in MeOD after aging for 25 days.

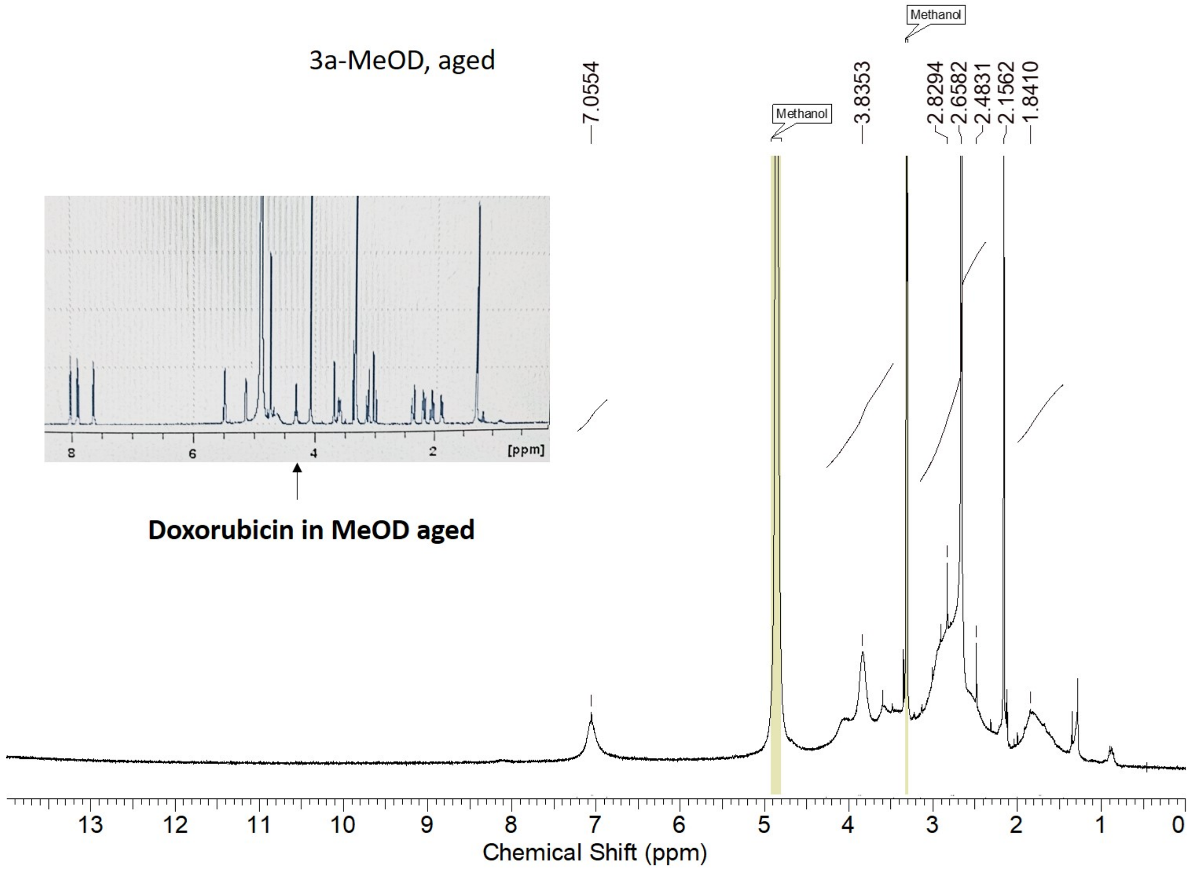

Figure S31. ^{1}H-NMR of **3a** in MeOD after aging for 25 days. In the inset is displayed the spectrum of Doxorubicin in MeOD after aging for 25 days.

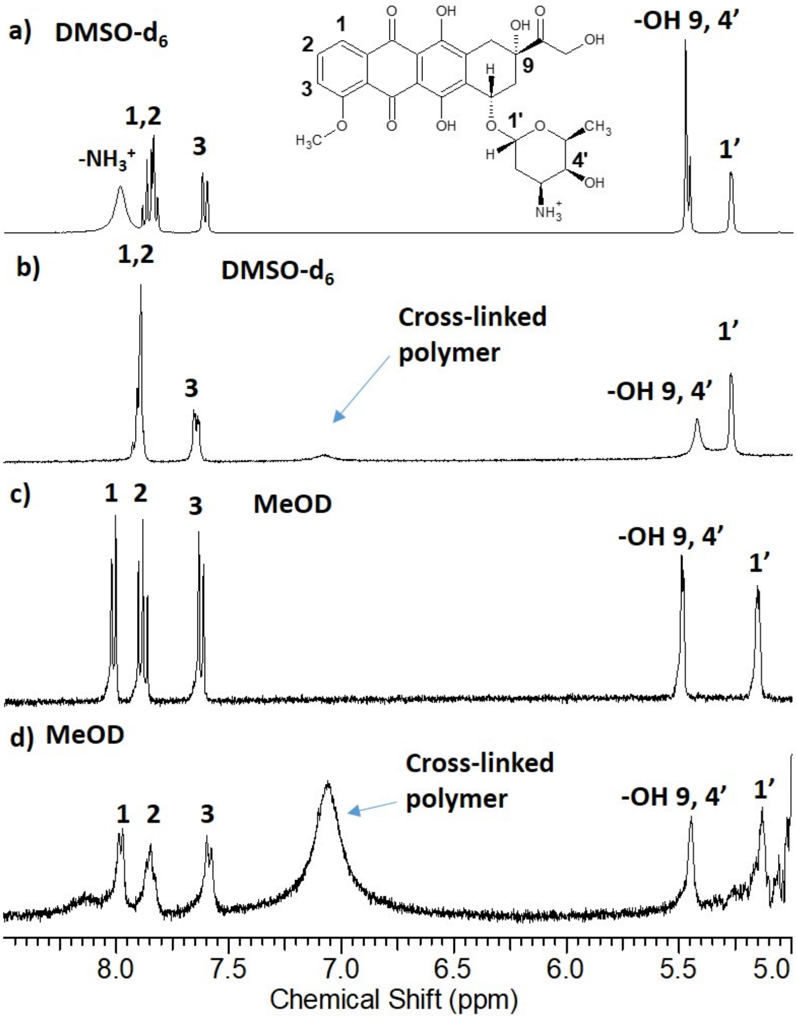

Figure S32. ^{1}H-NMR spectra, range 5.00-9.00 ppm: a) Doxorubicin in DMSO-*d6*; b) Doxorubicin mixed with 3a in DMSO-d6; c) Doxorubicin in MeOD; d) Doxorubicin mixed with **3a** in MeOD.

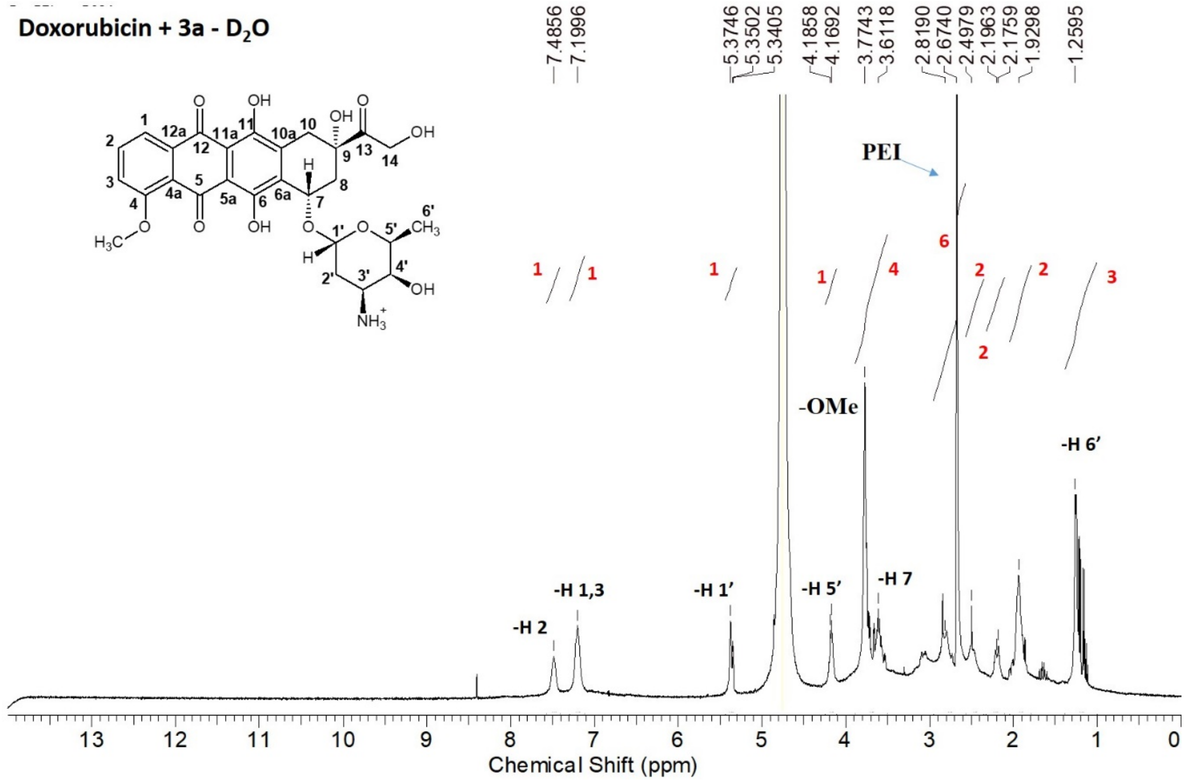

Figure S33. ^{1}H-NMR of Doxorubicin mixed with **3a** in D_2O.

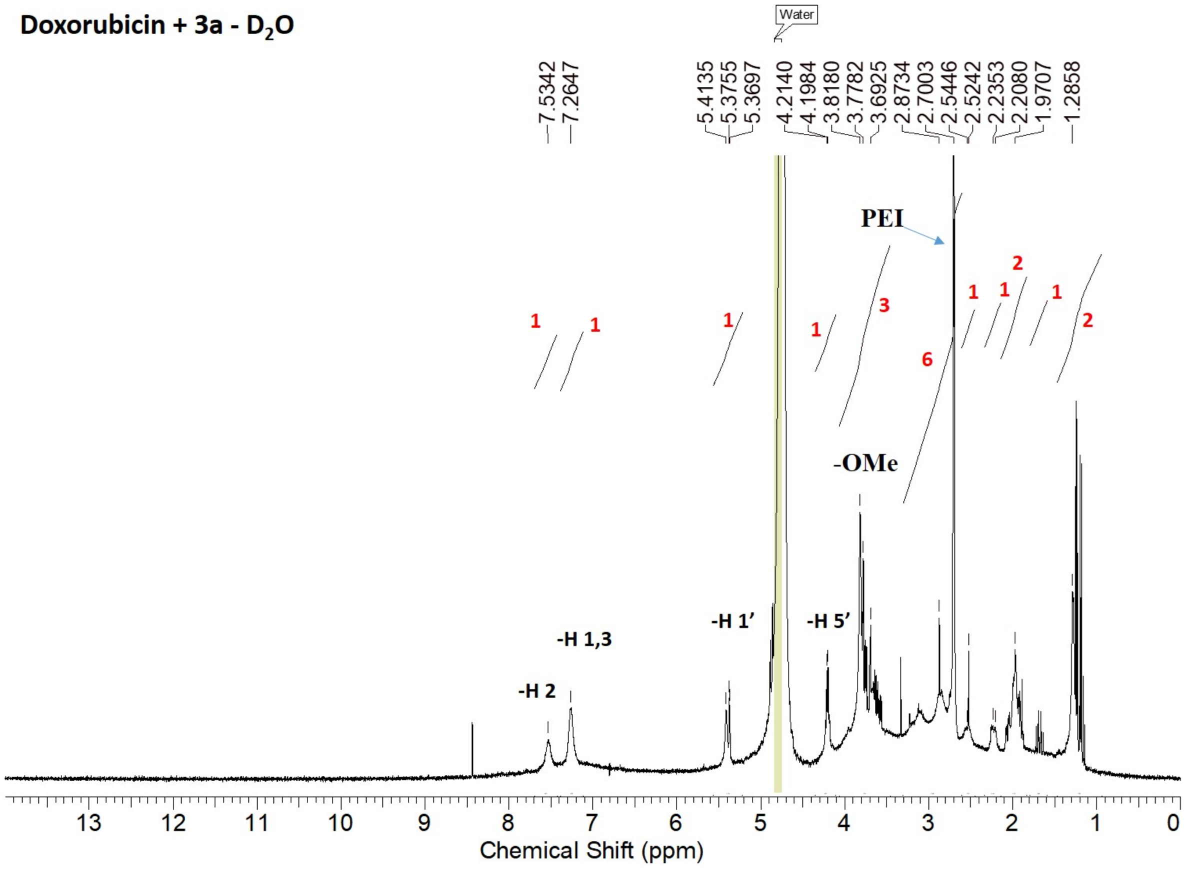

Figure S34. ^{1}H-NMR of Doxorubicin mixed with **3a** in D_2O aged 25 days.

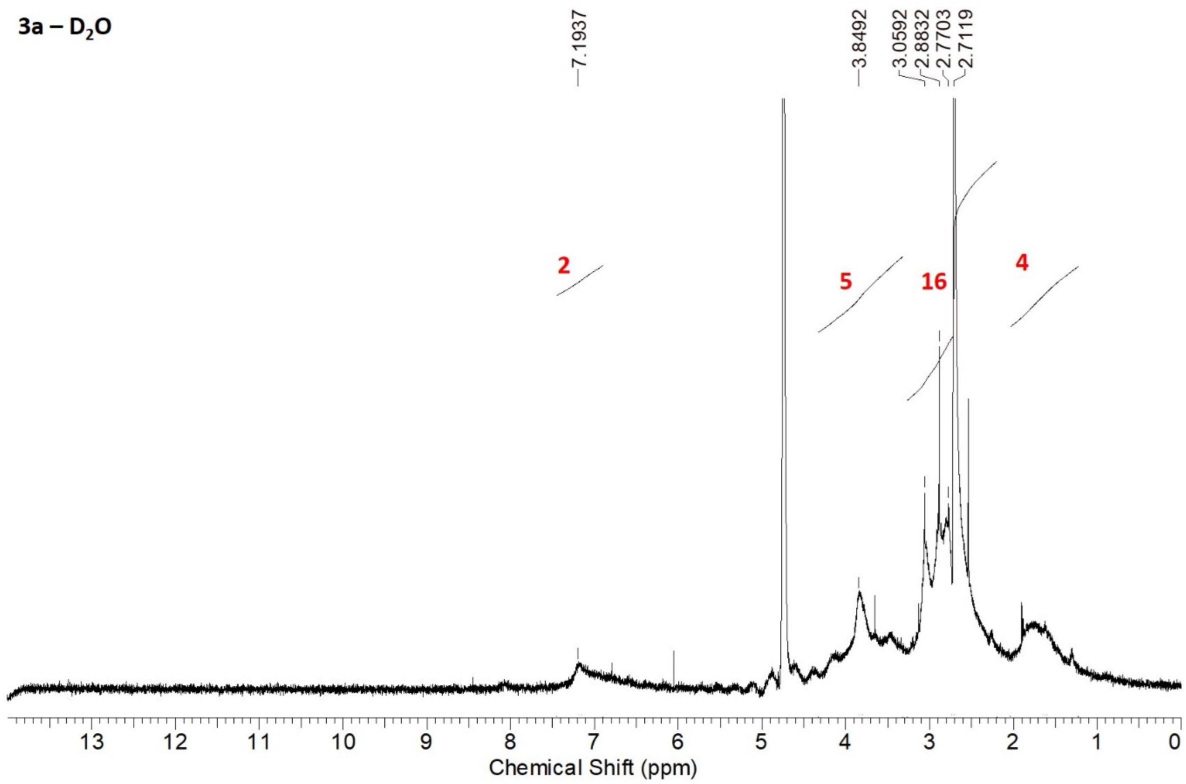

Figure S35. ^{1}H-NMR **3a** in D_2O aged 25 days.

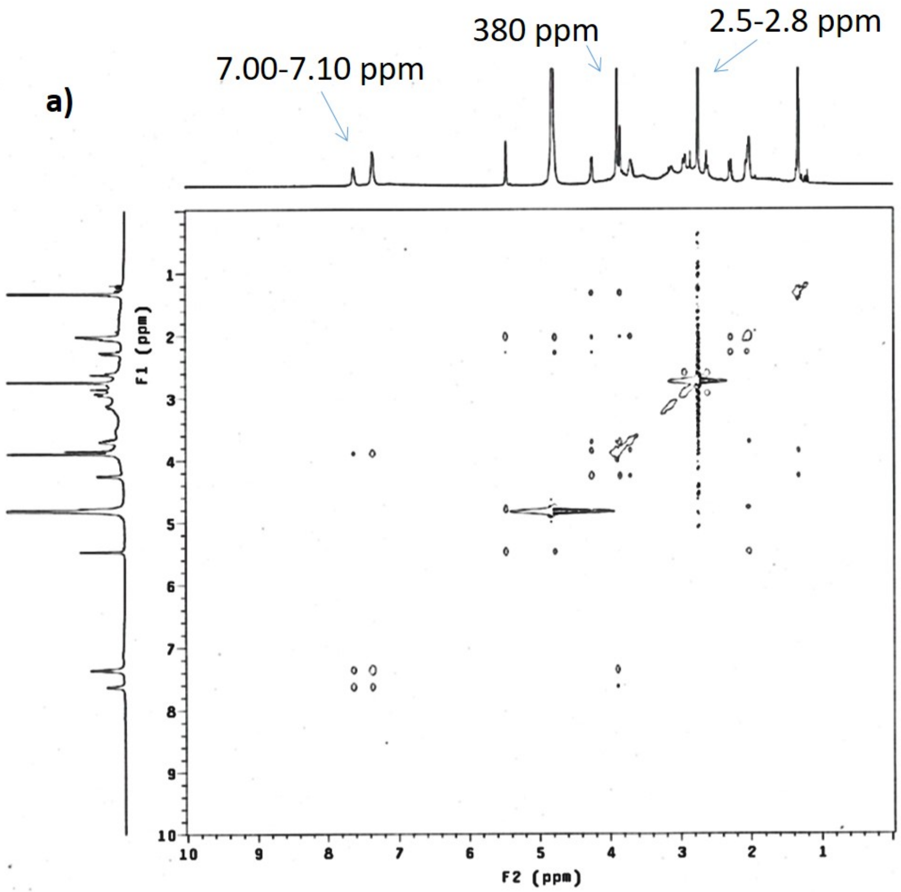

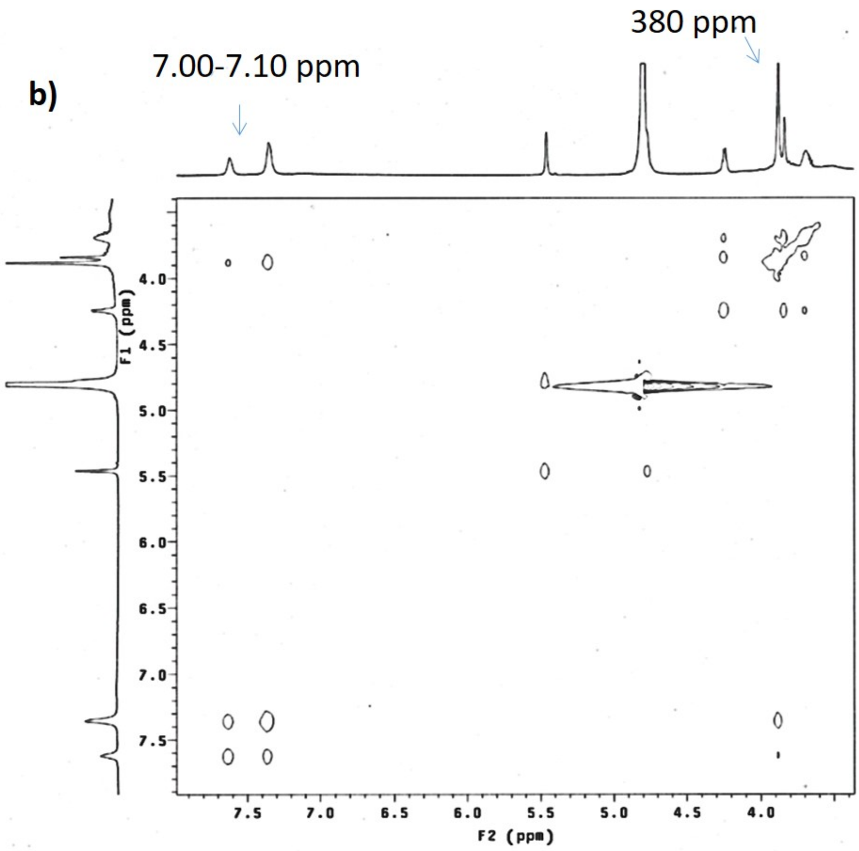

Figure S36. NOESY spectrum of Doxorubicin mixed with **3a** in D_2O. a) Spectrum range 0 - 10 ppm. b) Spectrum range 3 - 8 ppm.

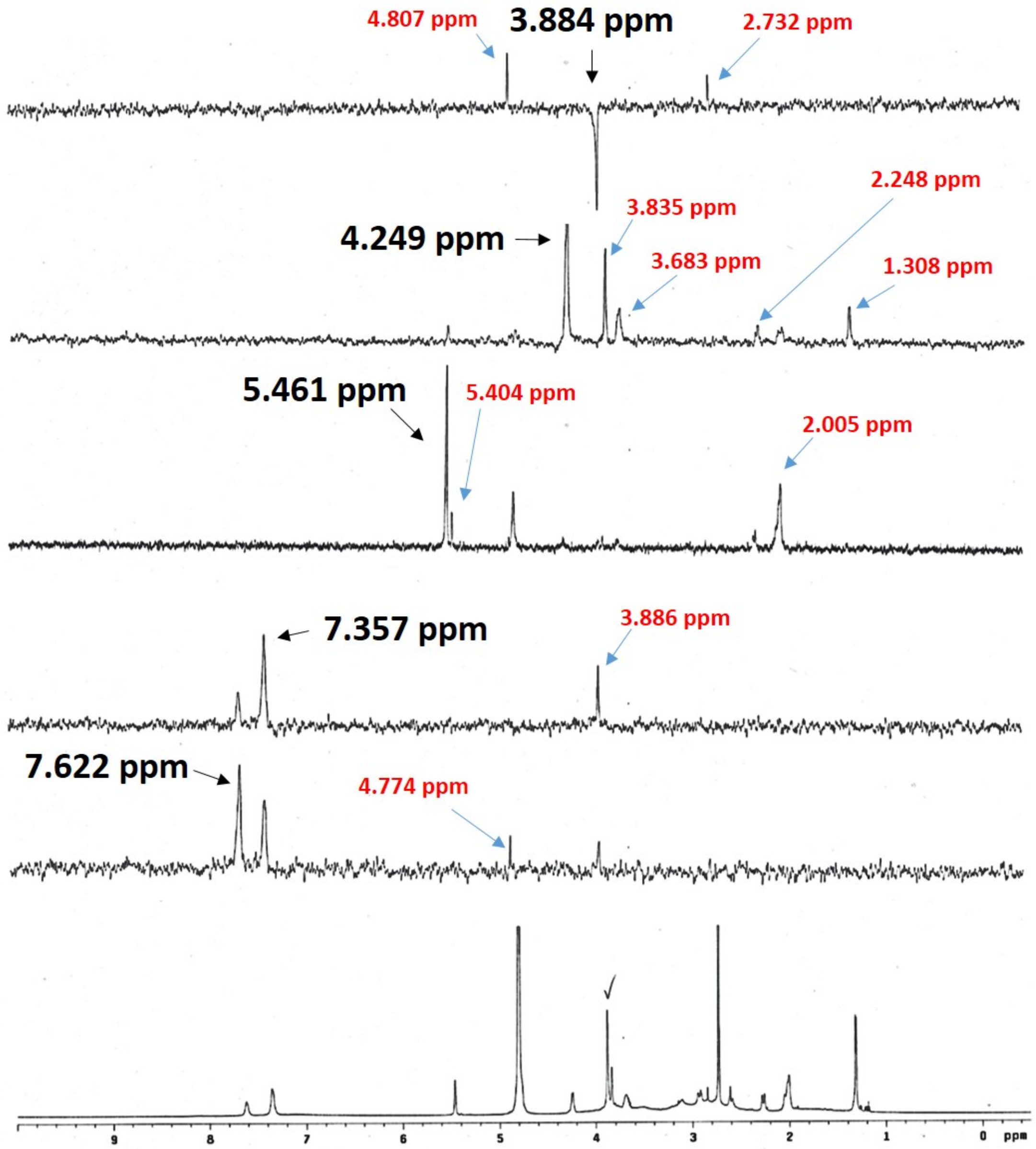

Figure S37. NOE spectra of Doxorubicin mixed with **3a** in D_2O. The irradiated signals are written in black and in red are written the signal that fill the NOE.

Analysis of NOE and NOESY spectra. The NOESY spectra allowed to understand that the two aromatic signals are in spatial relationship each other and with the signals at 3.88-3.84 ppm. The irradiation at the single signals allow to understand that the exact peak that is close to the aromatic signals is the one at 3.88 ppm, not at 3.84. It means that the signal at 3.84 ppm can be the –OMe signal of **3a**. In the NOESY it is possible to verify that the signal at 3.84 ppm is correlated to the signal at 1.25, 2.20, 3.65, 4.28 ppm. The irradiation at 3.88 ppm did not produced enhancement of the aromatic signals. In that region there are other -O-CH- groups like –H4′ of Doxorubicin. It is reasonable that the irradiation involved this proton that, as proven by NOE spectrum, lays in proximity of the PEI protons and to the signal at 4.81 ppm that is partially covered by the solvent but it is assigned to the H3′ and H14 of Doxorubicin (as found in literature). Basing on these considerations we could draw the map of figure 6.

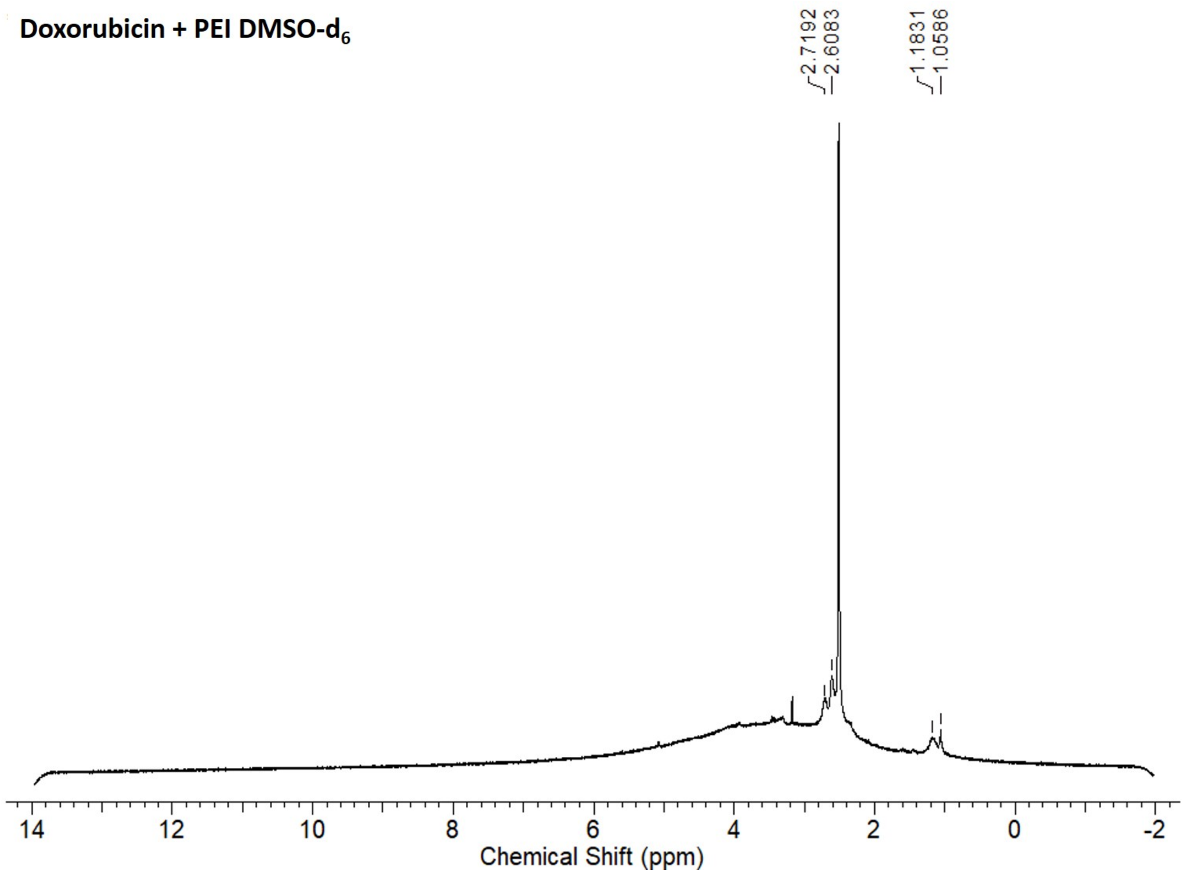

Figure S38. ^{1}H-NMR of Doxorubicin mixed with PEI in DMSO-d_6.

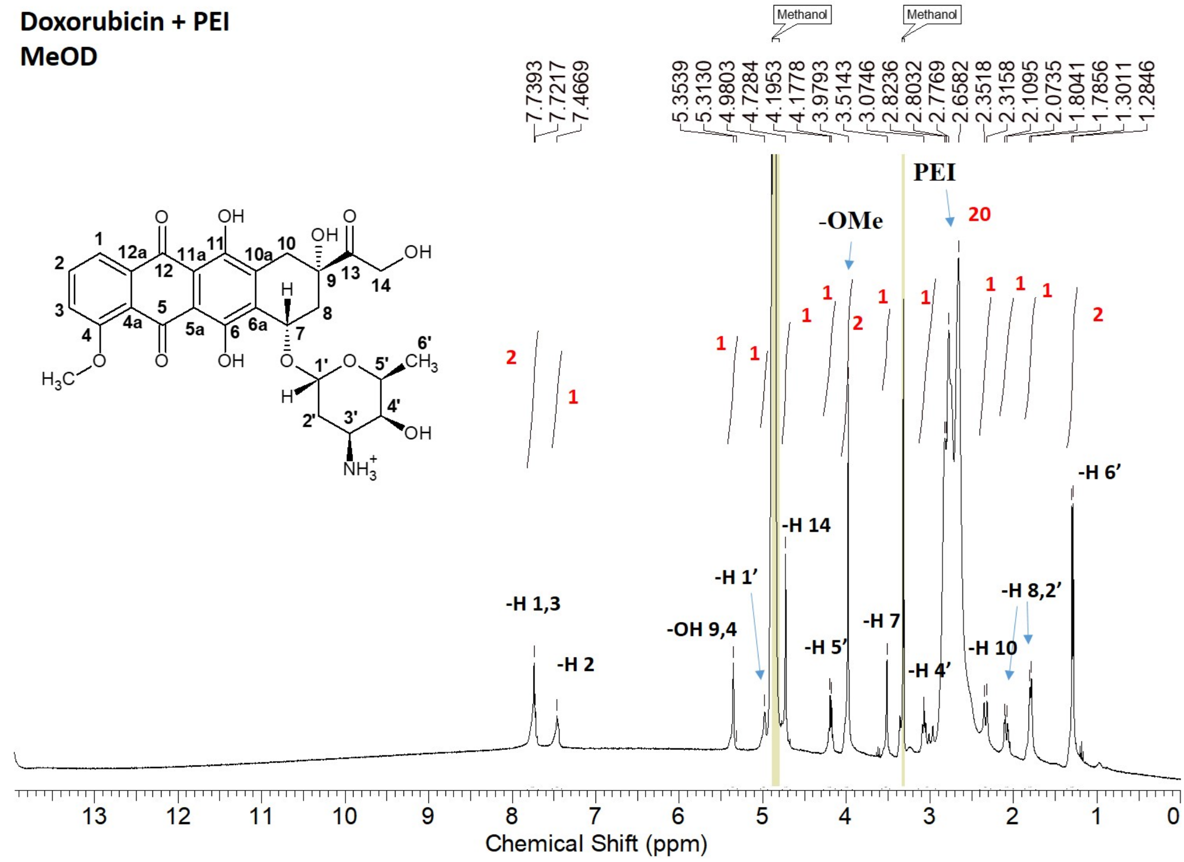

Figure S39. ^{1}H-NMR of Doxorubicin mixed with PEI in MeOD.

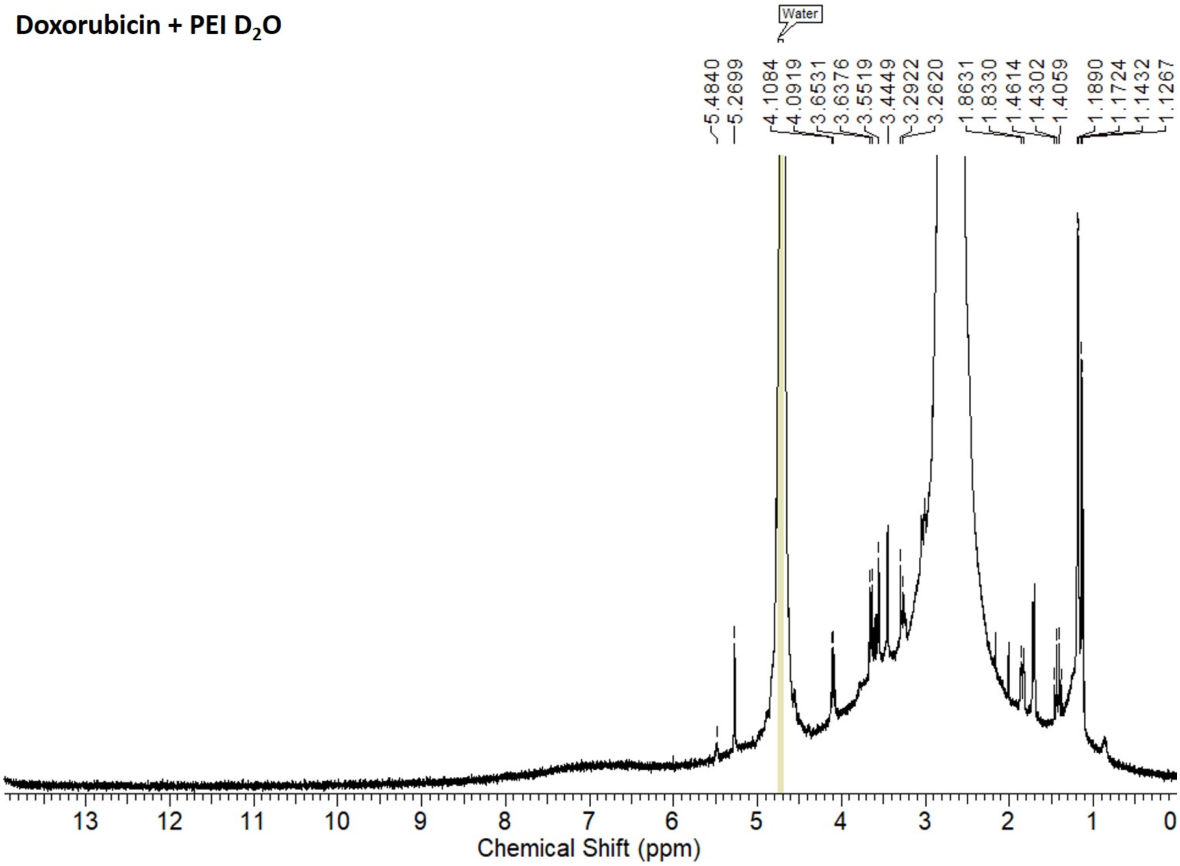

Figure S40. ^{1}H-NMR of Doxorubicin mixed with PEI in D_2O.

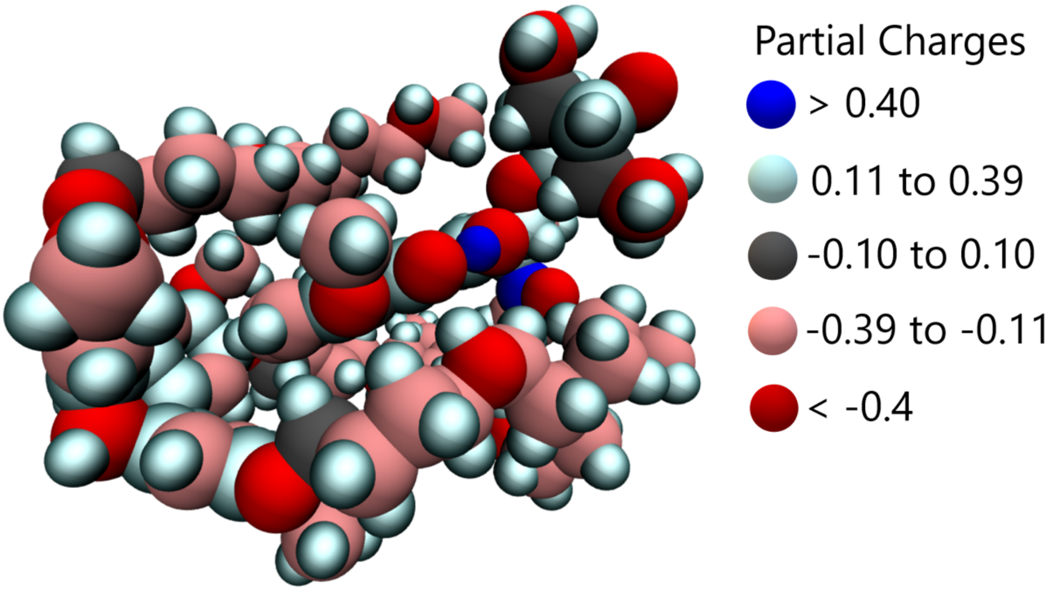

Figure S41. Electrostatic potential map of Doxorubicin-polymer complex in the configuration in Figure 8A (refer to main text). based on Mulliken population analysis. Polar interactions is comprised of partially positively charged aliphatic hydrogens of the ethyleneimine alkyl chains in contact with partially negatively charged oxygen atoms of Doxorubicin.

Table S1. Gibb's free energy of interaction (ΔGint) of the most stable Doxorubicin-polymer complex in vacuum, water and methanol.

Solvent	ΔG_{int} (kcal/mol)
Vacuum	-11.1
Water	-6.1
Methanol	-4.9

Table S2. Comparison of Grimme's dispersion correction Energy with BJ damping calculated using the Gaussian16 package and gCP-D3 Webservice.

Gaussian16 Package[1]	gCP-D3 Webservice[2]	Difference
Figure 8A	-15.530	-15.526
Figure 8B	-14.902	-14.949

[1]Calculated as difference between corrected and uncorrected thermal free energy (G(B3LYP-D3(BJ)) – G(B3LYP))

[2]Calculated by inputting the structure optimized at B3LYP to the gCP-D3 Webservice (http://wwwtc.thch.uni-bonn.de)

Table S3. X, Y and Z Coordinates (Å) of DFT optimized polymer-doxorubicin complexes shown in Figure 8 of main text. The coordinates are listed in order of the molecular formula $C_{87}H_{37}N_{13}O_{17}$

8a Configuration

1.5373	4.99613	-6.40456
-0.32258	-1.48078	-5.2493
3.91365	4.00639	-5.22239
5.66648	2.21077	-5.15332
3.86994	0.45811	-5.12676
3.28487	5.1697	-4.79314
3.2051	-0.66281	-4.63654
5.10006	3.52757	-4.63123
5.0354	0.96653	-4.53396
-1.65548	-1.24222	-4.51864
0.86173	-0.89812	-4.49066
3.16823	-3.61982	-3.81252
3.86514	5.91358	-3.73762
5.68002	4.26829	-3.59463
3.74431	-1.33819	-3.5277
5.55252	0.3111	-3.39784
-1.81688	-2.06422	-3.23531
5.0489	5.45456	-3.17414
4.89981	-0.84007	-2.93338
6.94797	3.85154	-2.85451
6.80032	0.7681	-2.64959
-1.72329	2.79566	-2.44021
3.48521	7.65982	-2.11412
1.29621	1.84154	-1.69489
6.66063	3.10299	-1.55883
-3.72544	-3.89322	-1.52872
6.54202	1.70631	-1.47223
-0.02508	1.56209	-1.32559
-3.25857	-2.44949	-1.29304
1.39115	6.71897	-1.0098
2.91824	6.85328	-0.94464
2.33164	1.78382	-0.76607
6.45688	3.86095	-0.39392
-3.70315	-5.99176	-0.24327
6.19373	1.12543	-0.2379
-2.18629	-6.22811	-0.10514
0.80302	5.73683	0.00573
-0.33239	1.20786	0.01237
-5.4188	-4.2911	0.17542
-1.70724	0.86831	0.45635
2.05052	1.44235	0.55433
6.11396	3.29292	0.82466
5.96385	1.8907	0.9059
0.74008	1.15128	0.94167
-8.41985	-0.77949	1.32858
-8.28287	1.75687	1.66847
-5.573	-4.08437	1.68718
-1.93214	0.52479	1.86516
-7.66815	0.38229	1.95987

5.39089	-0.0139	2.27394	6.75181	2.18652	-5.02924
-3.23946	0.25831	2.32051	2.65467	-3.44468	-4.76289
-1.86725	-6.38099	2.32477	0.7157	0.18004	-4.33463
0.51146	0.76672	2.35607	-1.74271	-0.17516	-4.27042
0.47097	5.24638	2.37139	4.20216	-3.93388	-4.00722
-7.47238	2.86494	2.38074	0.98474	-1.3698	-3.51105
4.71149	4.2701	2.48556	7.5994	3.26559	-3.50695
-6.59725	-6.11619	2.59228	-1.85275	-3.12825	-3.50204
-0.84907	0.47305	2.78922	7.51189	1.22443	-3.34175
-3.48548	-0.03276	3.69798	-2.36415	2.66305	-3.3137
4.92943	-0.24303	3.70686	2.65257	-4.40079	-3.24921
0.87066	5.6432	3.79529	1.49383	2.0977	-2.7308
-7.40853	2.54948	3.88231	7.51379	4.75764	-2.60789
4.46355	-1.68021	3.96787	-1.0631	3.65845	-2.59569
-1.09021	0.12006	4.12528	-0.93143	-1.94071	-2.58266
-4.92668	-0.21281	4.15628	5.50999	6.0238	-2.37491
-6.96641	1.11119	4.15806	-3.00253	-0.71892	-2.30768
-2.41629	-0.14368	4.57332	7.30135	-0.1257	-2.25967
-1.17648	5.40707	5.10425	-4.58142	-3.88837	-2.22623
4.03987	-1.88584	5.42467	3.00093	8.63967	-2.1771
-2.15544	4.32245	5.56351	5.29643	-1.38834	-2.08307
-3.74586	-2.80643	5.60412	-2.92708	-4.45307	-2.02446
-5.0656	-0.62006	5.64192	1.10265	6.39116	-2.01589
1.88195	3.44016	5.90479	4.56219	7.83565	-1.99527
-2.60295	-0.55319	6.01419	-2.34553	2.9502	-1.55448
0.95217	4.65064	6.02344	-4.07481	-6.51305	-1.14883
-2.69912	-3.76935	6.18154	3.35109	1.99671	-1.07212
-3.87345	-1.38582	6.21606	-1.6666	-5.81919	-0.98285
-4.40313	3.91999	6.40254	3.3765	5.85633	-0.93359
2.97137	-3.40946	7.00373	0.93083	7.70974	-0.86973
2.14794	-4.6967	7.10846	-4.02191	-1.91675	-0.71307
3.51728	2.09948	7.11548	-2.35207	-2.48541	-0.65788
0.38348	-5.68931	8.48319	6.58052	4.9402	-0.41514
2.63666	-5.39528	9.41115	-5.74816	-3.35973	-0.29955
-0.41203	-5.50582	9.78022	1.19834	4.73072	-0.18865
2.86788	-4.42436	10.57546	-6.11409	-5.07313	-0.18172
-2.47699	-6.131	10.90117	6.1198	0.04496	-0.17751
4.09836	-4.09036	12.65094	-0.29324	5.67618	-0.14986
2.21286	4.95281	-7.26954	-1.99711	-7.30999	-0.11707
0.64857	5.57185	-6.6704	3.21881	7.33773	-0.00796
-0.35599	-1.02022	-6.24455	-8.37129	-0.70729	0.23647
5.49274	2.17058	-6.23508	-3.91996	0.47979	0.58432
1.23908	3.97366	-6.13351	-8.27834	1.9378	0.58572
3.48001	3.43892	-6.03848	-4.21051	-6.42959	0.61904
3.4502	0.93859	-6.00653	-1.90332	-4.70561	1.17294
-0.16002	-2.55558	-5.40689	-7.91339	4.48633	1.21905
-2.49222	-1.47759	-5.18674	2.83536	1.3938	1.2997

0.87845	7.05966	1.5475	-5.15661	0.29259	6.24164
4.62166	-0.34243	1.56026	4.20755	2.05292	6.25195
-6.82996	-6.5602	1.6174	1.53879	5.55336	6.28585
-9.47325	-0.77364	1.62322	-1.71531	-1.08542	6.36577
-6.62578	0.38154	1.62593	1.30551	-4.60036	6.41653
-7.9732	-1.73068	1.63368	-1.77031	3.83453	6.46866
4.02703	4.73114	1.76118	-5.3696	4.40476	6.57888
-6.56511	-3.62039	1.8658	-2.69431	0.33083	6.65986
-6.45173	2.84946	1.97817	2.75207	-5.55438	6.74715
-4.8151	-3.35984	2.00042	0.28096	4.47051	6.86767
6.3152	-0.56822	2.05581	2.89839	1.19427	7.09296
0.77213	4.20555	2.20377	3.2255	4.10371	7.27934
-1.48066	-7.40523	2.24952	2.33243	-2.57268	7.31092
-0.63122	5.27066	2.26415	-4.04665	3.53402	7.36593
-9.01917	4.16526	2.36437	-0.24499	-5.33303	7.65868
-2.93834	-6.43224	2.58016	3.81845	-3.43113	7.71699
4.29748	3.31914	2.83529	-4.87946	-1.91363	7.83145
-9.71316	1.30234	2.89627	-2.94713	-3.17608	8.01528
-7.49633	-5.56324	2.92626	4.11406	2.07041	8.03339
-1.33933	-5.89662	3.15571	0.54675	-6.77057	8.31829
-6.41892	-6.93935	3.29223	3.59239	-5.5551	8.88782
3.60792	-1.91821	3.3237	-2.23352	-5.71579	8.92649
4.83501	4.94538	3.33598	2.35174	-6.37377	9.8233
-5.11098	-4.999	3.42685	-0.49164	-4.42194	9.99563
-5.40257	-0.96979	3.52709	3.14221	-3.43052	10.17124
5.26221	-2.39327	3.71221	0.12826	-5.95433	10.62483
1.94481	5.46626	3.90421	-3.45859	-6.58775	10.73355
4.09924	0.44441	3.90598	4.73094	-5.11274	11.01323
0.721	6.7327	3.93809	1.92579	-4.28316	11.11957
-1.57083	5.86036	4.18635	-2.63777	-5.11292	11.30827
-8.40649	2.68629	4.31869	-1.96679	-6.71511	11.67717
-6.73861	3.24939	4.38744	4.39398	-3.0579	12.38296
5.7451	0.02943	4.38924	3.18782	-4.02369	13.2584
0.73926	0.23427	4.55022	4.88619	-4.52002	13.279
-2.2066	3.53743	4.78497	-3.0546	-1.71058	-2.53989
2.51894	3.52936	5.00202	-4.04375	-4.56234	-0.26135
-3.8436	5.34158	5.06539	-1.55979	-5.66228	1.08809
-7.18642	0.83099	5.19582	1.14584	6.08772	1.38452
4.03663	-3.92127	5.34233	-8.01255	4.21448	2.19755
-2.7956	-4.71592	5.64591	-5.39914	-5.29063	2.49648
3.30852	-1.11173	5.69548	0.15516	4.86682	4.81106
-4.56449	3.05711	5.72966	3.39458	-3.18018	5.62394
1.26732	2.54402	5.75488	-3.45142	4.90395	5.89829
-5.98236	-1.20606	5.77061	2.65306	3.27334	7.13269
-1.14431	6.21099	5.86138	1.62749	-4.91763	8.45612
-1.69105	-3.3677	5.98623	-1.70361	-6.17379	9.66796
4.91363	-1.72896	6.08929	3.85283	-4.96302	11.50896

2.1288	5.67783	-5.31292
2.06114	-1.09996	-5.26758
3.20906	7.05925	-3.38461
3.14468	-2.45193	-2.98344
-0.95729	1.59014	-2.32886
-2.66917	0.88427	-0.34623
-4.28748	0.24898	1.4873
6.01947	4.1007	1.93163
-9.65629	1.80643	2.06664
5.61879	1.39034	2.12834
1.47485	0.69932	3.15
-7.69565	0.16426	3.39562
-5.56686	1.05754	3.92544
-4.47958	-3.1541	4.69623
-0.10926	0.02598	5.03531
-2.89976	-4.03851	7.56315
-4.00594	-1.52651	7.64861

Figure 8b Configuration

3.85559	3.25363	-8.09443
4.49166	2.05926	-7.68201
3.19308	3.33399	-9.32235
4.53418	0.98374	-8.59969
3.17729	2.2361	-10.178
3.86624	1.07755	-9.82587
4.68461	4.43127	-6.01535
5.17175	3.19108	-5.513
3.9737	4.49226	-7.2849
5.08307	1.97218	-6.31845
5.57813	5.56439	-4.03629
5.94046	4.34786	-3.47829
4.90888	5.60692	-5.28999
5.77267	3.14965	-4.23822
7.0852	6.73641	-2.40818
6.76001	5.62875	-1.41069
5.90879	6.87855	-3.37524
6.53482	4.25382	-2.07888
5.93618	3.22573	0.04183
4.90769	2.23232	0.58168
7.55846	1.48046	-0.31762
5.14944	0.81225	0.04232
6.62509	0.41174	0.2751
8.45464	6.49796	-3.09442
8.99197	7.52262	-4.09706
4.62647	-1.28165	-7.89633
9.03257	1.22555	-0.0519
6.73361	-1.80257	-3.06635
8.30725	6.44207	-8.2178
13.1535	1.04294	-0.23379
13.10672	1.46264	1.23011
10.96949	-1.67268	-3.92291
9.69439	-2.15557	-3.65693
11.24942	-0.86042	-5.02984
8.63414	-1.78831	-4.50353
10.205	-0.55409	-5.92591
8.91001	-1.01056	-5.62806
12.67409	-0.34242	-5.17379
10.43014	0.18563	-7.24747
10.69345	4.0179	-6.98555
9.4128	4.43147	-7.36706
11.09033	2.67837	-6.95245
8.49918	3.4426	-7.77031
10.14042	1.68354	-7.27272
8.87217	2.09963	-7.69082
12.55224	2.38023	-6.62672
13.33485	3.37934	-3.03384
13.33353	2.14285	-2.35513
13.10338	3.3964	-4.40135
13.13539	0.976	-3.09142
12.86218	2.2297	-5.14422
12.89724	0.99666	-4.47631
12.42406	0.41544	2.11856
12.34309	0.86151	3.58383
7.0785	-1.42929	5.81363
6.9039	-2.61491	4.85783
7.34479	1.02348	5.56156
9.23543	-0.47943	5.27044
10.04391	0.31466	4.2408
5.84367	1.29255	5.4088
5.90378	-3.29545	2.74291
8.57606	7.9388	-8.1074
7.79835	8.77308	-9.14082
6.27221	8.80938	-8.97379
2.16493	10.81771	-4.89421
1.79833	9.42041	-4.38574
2.32684	12.03276	-6.9993
4.08241	10.46112	-6.36166
4.43122	9.73584	-7.66472
0.8988	12.0156	-7.55305
-0.76171	13.31696	-8.76923
5.38281	-2.49051	-2.92206
4.37211	-2.11066	-4.00855
3.01402	-2.81563	-3.84443
1.44543	-1.10835	-4.69675

0.33953	-0.87954	-5.73188	2.34325	-2.60461	-5.76092
-1.68954	0.44145	-6.03612	0.82357	4.05588	-6.95602
0.50014	1.52021	-6.18973	-4.54185	-0.77077	-4.81481
0.35556	2.82442	-5.40042	6.61086	-0.71106	-3.10349
-2.63597	-0.0749	-4.94311	7.38382	-2.04837	-2.22076
-4.70488	1.16285	-5.40417	8.5388	6.08952	-9.23263
7.05292	3.52774	-9.63262	7.26443	6.19603	-8.00299
9.23612	-4.33188	-2.85943	12.16404	0.69972	-0.56656
12.54494	5.05584	-1.5643	13.86443	0.21727	-0.38437
0.96369	8.19064	-2.45567	14.12449	1.67422	1.58366
1.0489	5.15275	-5.26019	12.55111	2.4072	1.29106
3.87471	1.22164	4.01515	11.76525	-1.96426	-3.24278
2.72374	4.27293	-9.5941	8.07732	-0.76664	-6.28323
2.66327	2.29315	-11.1335	12.9623	-0.27884	-6.22548
3.92833	0.23357	-10.5065	13.35129	-1.07803	-4.7243
5.84002	5.92784	-0.89475	11.4511	0.00084	-7.59387
7.55766	5.52997	-0.66682	9.78005	-0.28602	-7.99313
5.0541	7.25958	-2.7989	11.40337	4.80074	-6.73439
6.12917	7.62565	-4.13837	8.12929	1.35676	-7.97215
7.49394	3.7327	-2.14029	13.15121	3.21209	-7.01474
5.89327	4.16939	0.60107	12.88212	1.49796	-7.18152
3.89844	2.5603	0.31733	13.11007	4.36595	-4.89176
4.97924	2.23609	1.67514	13.16831	0.01718	-2.58524
7.37706	1.50596	-1.40053	11.41035	0.23703	1.7387
4.98396	0.84064	-1.04072	12.94671	-0.54862	2.06796
6.82727	-0.53026	-0.25161	12.01829	1.92064	3.61888
4.57018	-0.4835	1.49845	13.34696	0.82691	4.02527
4.07044	-0.93388	0.01694	6.08143	-1.09621	6.10997
7.78872	7.89005	-0.97034	7.58897	-1.75988	6.73691
9.11296	7.00035	-5.0538	7.88031	-3.09282	4.648
10.00543	7.79066	-3.75653	6.29134	-3.37695	5.35913
7.93213	9.01616	-3.4676	7.87755	1.73853	4.92825
3.84321	-1.59983	-8.59803	7.62241	1.25676	6.61087
4.1944	-1.08815	-6.91062	9.58583	-0.21642	6.29245
5.38183	-2.06517	-7.81267	9.47569	-1.53716	5.12441
4.03572	6.65247	-6.59416	9.698	0.03156	3.24075
6.09258	1.30714	-4.4104	9.85743	1.3997	4.34548
6.54594	0.81146	2.24342	5.27898	0.8072	6.22697
9.2218	1.19676	1.02435	5.69439	2.37129	5.55161
9.63883	2.0179	-0.50214	5.14135	-3.94152	3.19584
9.33418	0.26622	-0.48714	5.4962	-2.89537	1.80882
6.80759	-1.51795	3.14782	6.77294	-3.93086	2.4909
11.71886	0.11453	5.36465	9.65128	8.10226	-8.252
5.43131	-0.08652	3.97849	8.34835	8.2822	-7.0904
2.08711	9.88261	-2.4269	8.1613	9.80649	-9.09583
6.15902	8.94931	-6.93975	8.02779	8.39917	-10.1494
1.25518	13.40364	-8.99548	5.8455	7.78976	-9.05447

5.85315	9.37555	-9.81635
1.26503	11.43746	-4.84178
2.9005	11.28523	-4.20847
2.64574	8.72228	-4.52624
0.9766	9.03302	-5.00142
2.46544	12.93515	-6.37732
3.02183	12.12968	-7.84238
4.32694	9.7947	-5.52721
4.72989	11.35034	-6.25733
3.8189	8.81709	-7.73174
4.1465	10.35528	-8.52613
0.77986	11.12285	-8.19638
0.18016	11.90046	-6.73071
-1.48078	13.29939	-7.94137
-0.90817	14.25427	-9.3167
-1.01466	12.47806	-9.44563
4.98384	-2.22516	-1.93131
5.53815	-3.57822	-2.91112
4.23628	-1.02133	-4.00546
4.79726	-2.36552	-4.987
2.60461	-2.60808	-2.84584
3.16585	-3.90311	-3.89418
2.21143	-0.31722	-4.78629
1.00655	-1.00249	-3.69711
0.74204	-1.03796	-6.7538
-0.41922	-1.65276	-5.58027
-1.96267	1.47298	-6.28183
-1.84403	-0.15133	-6.95564
1.55738	1.24034	-6.18354
0.23161	1.68781	-7.25196
0.72613	2.65317	-4.38248
-0.71487	3.0897	-5.29812
-2.34092	-1.0932	-4.66429
-2.50132	0.55472	-4.04367
-4.28383	1.81577	-6.1767
-5.76217	1.01411	-5.6473
-4.64277	1.70428	-4.44089
7.14581	2.46022	-9.86416
6.04381	3.8628	-9.87824
7.78279	4.08679	-10.2329
9.10697	-4.84199	-1.90176
8.32588	-4.43452	-3.46009
10.08055	-4.78029	-3.39934
11.63549	5.21632	-2.15645
12.3273	4.3727	-0.73611
12.89642	6.00995	-1.16206
0.08459	7.81375	-2.99217
0.68817	8.29903	-1.40102
1.75224	7.4164	-2.52927
1.48911	5.01843	-4.26533
0.01374	5.51577	-5.12005
1.62038	5.92656	-5.78028
3.47016	0.85018	3.06959
3.72165	2.30737	4.04512
3.29113	0.78018	4.84288
4.17955	-0.12456	0.62716
6.21862	-2.18988	3.64052
11.46834	-0.00077	4.38359
7.78439	-0.31727	5.16371
5.30905	0.92371	4.09667
1.34117	9.48893	-2.99921
2.65763	10.80185	-6.27417
5.86938	9.49713	-7.7495
0.59285	13.2717	-8.23198
1.9782	-2.46522	-4.81881
-0.28927	0.43592	-5.59976
1.14365	3.89825	-6.00128
-4.02308	-0.12794	-5.40747
5.60839	3.45616	-1.32173
7.26234	2.78278	0.23234
7.22112	8.0197	-1.74883
8.19017	8.66264	-4.33747
9.16016	5.55817	-2.77484
5.3087	-0.1235	-8.38055
3.51709	5.56704	-7.73154
5.47557	0.87607	-5.86365
4.50818	6.81589	-5.72985
6.24259	2.00935	-3.7156
6.89693	0.11774	1.64649
9.16886	5.77749	-7.27566
13.54516	2.18047	-1.00552
7.34931	-2.24434	-4.29282
7.23797	3.78058	-8.23751
13.60931	4.55806	-2.3811
9.50481	-2.96105	-2.55014

Figure 8c Configuration

-2.04369	-1.75585	5.69887
-2.73183	-0.51523	5.76511
-1.89745	-2.56872	6.82692
-3.28085	-0.12899	7.01404
-2.42826	-2.15909	8.04729
-3.11539	-0.95251	8.13482
-1.41717	-1.32134	3.29059

-2.08334	-0.06211	3.35302	-0.41417	-6.66957	6.92367
-1.42694	-2.22093	4.43286	-1.58188	-6.72495	7.91395
-2.78491	0.35988	4.56677	1.2696	-5.7538	8.4479
-0.72798	-0.82449	0.98483	1.38612	-5.30078	6.0363
-1.42522	0.37178	1.01472	2.03675	-3.91613	6.08085
-0.73403	-1.68521	2.12093	0.91818	-4.69935	9.50295
-2.08127	0.77588	2.21989	-3.60207	-7.96083	8.48737
0.48755	-0.10985	-1.11588	4.14827	5.94216	-1.9792
-0.73325	0.75945	-1.42738	2.61896	5.85476	-2.05985
0.06184	-1.27626	-0.2164	1.96981	5.20468	-0.8344
-1.51002	1.28376	-0.2009	2.15906	6.04274	5.23677
-3.26609	2.27998	-1.55097	3.2829	5.03289	5.48299
-4.78287	2.18179	-1.72844	-0.03596	6.14666	4.19029
-3.46281	4.25076	-0.16872	1.98182	6.21449	2.81695
-5.55343	2.90503	-0.61322	1.62371	5.38284	1.58139
-4.97129	4.3273	-0.43532	-0.86607	5.17365	5.03438
1.63738	0.69964	-0.48127	-3.03002	4.85824	6.11304
3.0588	0.12447	-0.5336	0.96259	-3.31919	-5.95833
-5.29415	1.12207	6.83785	-0.52968	-3.38835	-5.5951
-2.79043	5.60506	-0.002	-0.7802	-3.71469	-4.11882
1.74854	-2.2623	-5.18906	-2.44938	-4.36706	-2.45766
4.78456	6.51112	-3.24821	-1.99682	-5.77339	-2.01784
5.70888	-0.69977	2.11445	-2.95529	-5.8832	0.21772
4.89682	-0.80773	3.39842	-0.82261	-6.98671	-0.25478
5.4698	-1.76494	-2.81915	0.64465	-6.68798	-0.61653
4.33092	-2.22454	-3.47367	-2.82838	-5.23643	1.60585
6.39161	-0.89913	-3.41839	-2.85658	-7.0867	3.21333
4.10744	-1.84468	-4.80978	3.21674	3.47529	-7.4774
6.12665	-0.44111	-4.72365	3.81006	-4.29752	-2.36523
5.00463	-0.94746	-5.39418	5.37626	3.65329	1.86665
7.62791	-0.5114	-2.61314	5.11891	4.45501	6.9735
6.95304	0.64366	-5.40787	1.59687	-5.80501	1.46683
6.26373	4.09492	-3.8318	1.34437	-3.97798	11.79034
5.18553	4.54034	-4.58576	-1.34093	-3.49677	6.74333
6.89506	2.85659	-4.06146	-2.30189	-2.77941	8.93005
4.72792	3.72915	-5.65284	-3.5319	-0.60592	9.07554
6.42496	2.04471	-5.10115	-1.4121	0.12668	-2.01112
5.35451	2.51247	-5.89041	-0.43622	1.60786	-2.05485
8.04703	2.48934	-3.12758	-0.5411	-1.95734	-0.83422
6.87259	2.39956	0.51295	0.93669	-1.84306	0.10553
6.62316	1.02645	0.72538	-1.13078	2.27069	0.07792
7.35521	2.81471	-0.72282	-2.75002	2.00899	-2.48065
6.91874	0.13276	-0.30315	-5.08321	1.13169	-1.77335
7.59908	1.92644	-1.78343	-5.04281	2.63489	-2.69398
7.38702	0.55801	-1.55682	-3.28896	3.63354	0.71825
4.27898	-2.20544	3.54325	-5.40111	2.35754	0.32524
3.44958	-2.37106	4.81764	-5.47056	4.82446	0.40666

-7.14745	3.57691	-1.65729	4.08449	-2.1732	5.70471
-7.51212	3.2089	-0.11753	0.08262	-7.65714	6.91308
1.12958	-0.00991	-2.97682	-0.82554	-6.51035	5.91907
3.60948	0.59267	0.28681	-2.06223	-5.72791	7.96474
3.52702	0.47625	-1.4666	-1.21236	-6.94484	8.9239
2.99849	-1.71573	-1.24271	2.35045	-5.71132	8.24625
-5.87972	0.36034	7.36922	1.08339	-6.74914	8.87297
-5.40515	1.00092	5.75732	2.17514	-6.07375	5.92812
-5.64008	2.11577	7.1277	0.77483	-5.33995	5.13153
-0.15973	-3.32034	2.87348	1.24321	-3.1627	6.12943
-3.08316	2.06404	3.1463	2.64508	-3.79741	6.99887
-4.55013	4.92776	-2.22808	1.0812	-3.68988	9.07804
-2.97902	6.24782	-0.86678	-0.15366	-4.76626	9.73204
-1.70952	5.47825	0.11487	-3.20401	-8.30176	9.45049
-3.18092	6.11107	0.88833	-4.18764	-7.04078	8.67597
-2.88756	-7.59303	6.62057	-4.28656	-8.73198	8.11874
3.53717	-4.40232	4.79318	4.44032	6.57607	-1.13338
2.65677	-4.89436	10.53861	4.56128	4.94681	-1.77238
4.44618	6.32274	6.53908	2.20326	6.8655	-2.1996
1.98709	6.90303	0.28145	2.33299	5.28588	-2.95278
-2.68424	5.69428	4.29098	2.33637	4.17772	-0.71563
-2.78787	-3.40985	-4.22568	0.87821	5.12201	-1.00664
0.57281	-4.77984	0.04302	1.51143	6.03681	6.1177
-1.74222	-5.38594	3.31411	2.58012	7.06646	5.17206
1.38204	-1.25094	-5.43274	3.91808	4.93592	4.58024
1.67127	-2.40928	-4.11222	2.8323	4.04608	5.64661
5.86526	6.65969	-3.12233	-0.11967	7.16025	4.62271
4.34718	7.48557	-3.48916	-0.47957	6.19881	3.18887
5.09948	-1.02177	1.26093	3.0692	6.17958	2.93176
6.59931	-1.34454	2.1605	1.72479	7.27926	2.6372
5.53221	-0.56729	4.2605	1.97059	4.35391	1.72893
4.10093	-0.05228	3.3701	0.5242	5.32179	1.46179
5.62428	-2.08548	-1.79344	-0.81377	4.16751	4.57773
4.80506	-0.65288	-6.42089	-0.42663	5.081	6.03589
8.43608	-0.21207	-3.28356	-2.61746	4.94113	7.12593
7.98731	-1.41385	-2.10394	-4.05342	5.24924	6.14107
8.00846	0.55879	-5.13802	-3.07307	3.78536	5.85708
6.90659	0.48463	-6.49151	1.42945	-4.29259	-5.7592
6.63653	4.71313	-3.02339	1.07771	-3.13246	-7.03327
5.00598	1.88676	-6.70472	-1.02677	-2.44151	-5.85633
8.63055	3.39829	-2.93823	-1.00563	-4.16695	-6.20342
8.73343	1.79791	-3.62305	-0.49494	-2.85453	-3.48564
7.54993	3.87728	-0.84208	-0.11777	-4.53967	-3.8259
6.78877	-0.92979	-0.13582	-3.52881	-4.24816	-2.30896
3.63656	-2.41605	2.67908	-1.94914	-3.63151	-1.79997
5.07737	-2.96378	3.53289	-2.73714	-6.52403	-2.35264
2.65302	-1.61472	4.83621	-1.07181	-6.00731	-2.54967

-3.71076	-5.30032	-0.32027
-3.36931	-6.90608	0.30204
-1.14029	-7.92063	-0.76163
-0.89712	-7.15601	0.82137
0.7373	-6.525	-1.69865
1.24657	-7.57895	-0.39376
-2.30468	-4.28509	1.47418
-3.8533	-5.00949	1.96341
-3.81067	-6.74557	3.66071
-3.10081	-7.85634	2.47264
-2.2571	-7.56796	3.99401
2.40266	4.05614	-7.91564
4.00331	3.32862	-8.23027
2.83427	2.49282	-7.16678
3.03226	-4.66534	-1.68945
3.91224	-4.95817	-3.23473
4.76551	-4.25697	-1.83023
5.44444	4.33577	2.71734
4.8668	4.16357	1.03863
4.80815	2.76562	2.16212
4.68583	3.48377	7.2413
5.6905	4.81392	7.83606
5.82359	4.28449	6.13755
1.99594	-4.89184	1.9199
2.38381	-6.569	1.50447
0.7547	-6.15137	2.08897
1.4746	-2.91886	11.49738
1.97616	-4.17192	12.66346
0.30043	-4.10819	12.10042
-6.9845	2.88136	-0.9271
-2.50353	-7.79365	7.54298
2.81281	-3.68705	4.86306
0.52239	-5.57291	7.19021
1.65803	-4.93748	10.7378
4.04529	5.39539	6.67389
1.3611	5.71916	4.04905
2.28329	5.93247	0.39723
-2.22824	5.67354	5.20247
-2.1636	-4.13502	-3.87694
-1.72197	-5.86736	-0.57962
1.2498	-5.5426	0.06763
-2.08002	-6.01981	2.59302
-2.91425	1.38499	-0.51363
-2.83516	3.61218	-1.31
0.99021	-0.71229	-2.3202
3.16785	-1.27698	-0.3852
1.44449	1.80626	-0.00801
-3.91829	1.0596	7.22839
-0.91046	-3.36122	4.37071
-3.40547	1.44696	4.58996
-0.07344	-2.84664	2.0013
-2.66792	1.97746	2.23774
-5.25107	5.13176	-1.58528
4.52501	5.72232	-4.41503
6.11225	0.66367	1.9402
3.12203	-2.37119	-5.60536
3.68226	4.23067	-6.37453
6.72409	3.32192	1.52103
3.39631	-2.987	-2.78858

Figure 8d Configuration

1.15447	1.17551	-0.03523
1.09916	2.45501	0.57545
1.9529	0.16061	0.49772
1.89153	2.67785	1.72863
2.7263	0.40045	1.62994
2.69541	1.65182	2.23883
-0.33286	1.97345	-1.92311
-0.37183	3.266	-1.3295
0.40035	0.88665	-1.28007
0.28118	3.52444	-0.03965
-1.58218	2.84245	-3.85077
-1.65599	4.09939	-3.26277
-0.95462	1.76326	-3.16346
-1.04028	4.31459	-1.99105
-2.22807	3.82365	-6.08645
-3.00256	4.8736	-5.29747
-2.12044	2.56088	-5.23485
-2.28657	5.28029	-3.99892
-3.68309	7.20542	-3.55358
-4.47245	7.80191	-2.3878
-1.68681	8.43385	-2.96817
-3.55085	8.29436	-1.25985
-2.43534	9.18148	-1.86081
-0.84708	4.33349	-6.55975
-0.77236	5.64736	-7.33138
0.97998	4.17108	3.32998
-0.58574	9.23724	-3.64291
1.88987	-3.79629	-1.54783
3.70237	5.31385	-2.2126
5.73587	-2.46461	5.28499
5.13906	-2.73124	6.66025
5.84788	-3.52749	0.23377
4.75286	-4.1268	-0.38149

6.45376	-2.3668	-0.26985	-6.7929	1.54674	-6.06511
4.23712	-3.55196	-1.55479	2.57708	1.84095	-4.30651
5.96432	-1.81519	-1.47061	4.27197	-6.44118	-0.55686
4.86351	-2.42863	-2.086	6.18263	1.84105	5.81841
7.59317	-1.75482	0.53883	2.18029	11.65036	4.66267
6.56386	-0.58531	-2.14795	-3.18798	-2.94624	-10.8296
5.5432	2.80227	-0.64891	3.72157	-5.35688	15.58208
4.46275	3.1575	-1.4469	1.97572	-0.80039	-0.00147
6.24965	1.60074	-0.81151	3.36928	-0.37843	2.02853
4.05541	2.27705	-2.47181	3.30851	1.8686	3.10853
5.85814	0.73405	-1.84536	-3.97541	4.43875	-5.0416
4.77137	1.09471	-2.66095	-3.18678	5.75683	-5.91622
7.40593	1.31673	0.14131	-3.12841	2.12648	-5.17381
6.05027	0.82272	3.67129	-1.47489	1.82222	-5.71888
6.15538	-0.57067	3.86669	-1.47658	5.98257	-4.24167
6.48251	1.37327	2.47365	-4.32061	7.0854	-4.4393
6.69213	-1.34643	2.83999	-5.17629	7.06159	-1.99826
7.00012	0.59731	1.42364	-5.0659	8.64273	-2.77021
7.10106	-0.78983	1.61413	-1.26697	7.52192	-2.536
5.10297	-4.22654	6.99857	-3.06543	7.41599	-0.81895
4.50192	-4.50796	8.37782	-1.72884	9.45749	-1.07133
3.11576	-9.58298	11.58866	-4.53472	9.9101	-0.47745
2.08383	-9.87872	12.68202	-3.80241	9.00081	0.64444
4.28264	-7.73003	12.6867	-2.59704	2.79377	-7.72063
4.06436	-7.77483	10.24487	-1.04749	6.46659	-6.64354
3.9623	-6.26768	9.98715	-1.53266	5.63543	-8.12406
3.55749	-6.69216	13.55138	1.06395	5.14555	-7.48246
0.78429	-11.6551	13.72546	0.98863	3.40983	4.1215
3.66128	6.69784	-1.57176	-0.01247	4.23098	2.87406
2.46813	6.90073	-0.62786	1.24027	5.14153	3.75728
2.43104	8.26799	0.06299	-0.49903	-0.07458	-3.17701
0.92492	12.72766	1.91416	-0.61988	5.48071	-0.57763
0.27579	12.15966	3.19785	-3.31644	10.23423	-3.24233
-0.92207	12.83808	0.23397	-0.95562	10.20461	-3.99596
0.63875	10.93374	0.20928	-0.18808	8.68318	-4.5
2.0857	10.69565	-0.25551	0.23219	9.40573	-2.93301
-2.19304	12.14713	0.80986	1.30713	-11.5195	11.76676
-3.90965	13.73861	0.04334	4.00674	-6.43313	7.96058
0.75143	-4.19426	-2.47336	5.25476	-5.93981	14.38239
-0.60958	-3.82505	-1.86644	0.43885	12.54198	5.19311
-1.79846	-4.03774	-2.8096	3.01829	9.40417	-1.5185
-2.9154	-3.09007	-4.78271	-3.31369	12.00868	-0.88714
-2.62913	-2.32198	-6.08823	-0.9582	-3.61386	-4.61621
-4.45329	-0.6854	-6.08897	-3.33093	-1.68541	-9.22429
-4.69972	-2.76657	-7.37867	-5.13102	2.47233	-6.81278
-4.13635	-3.42782	-8.63931	1.836	-2.71865	-1.3467
-4.80082	0.47662	-7.03039	1.84084	-4.33289	-0.59138

4.56757	5.2281	-2.88275	-0.93072	13.90791	0.47803
2.79737	5.12424	-2.80032	-0.98752	12.76697	-0.86229
5.15348	-2.97162	4.50192	0.36877	10.18525	0.97536
6.76932	-2.83756	5.23058	0.00164	10.72941	-0.65596
5.72952	-2.18965	7.41072	2.79373	10.83604	0.58182
4.12674	-2.30864	6.68984	2.32116	11.45884	-1.00519
6.22638	-3.99323	1.13963	-2.36695	12.46623	1.8461
4.45894	-2.0255	-3.01029	-2.01839	11.06424	0.84425
8.31519	-1.26452	-0.11779	-3.22243	14.47579	-0.40925
8.14005	-2.56803	1.02998	-4.85515	13.78317	-0.50864
7.6274	-0.50555	-1.90966	-4.11239	14.06579	1.07127
6.51778	-0.74926	-3.23095	0.80411	-5.2705	-2.6884
5.83366	3.50869	0.12362	0.8984	-3.66411	-3.42206
4.48045	0.42209	-3.46021	-0.77614	-4.4117	-0.95188
7.85257	2.27744	0.42319	-0.59407	-2.7675	-1.57656
8.19784	0.76483	-0.37139	-2.71385	-3.7261	-2.291
6.38742	2.4496	2.36143	-1.92224	-5.115	-3.03612
6.78818	-2.41771	2.97605	-3.63132	-2.52428	-4.1723
4.51779	-4.76042	6.23431	-3.38842	-4.06606	-5.00312
6.11456	-4.65132	6.96786	-1.96513	-1.48773	-5.83409
3.48288	-4.075	8.43239	-2.06808	-2.97298	-6.77257
5.09525	-3.99165	9.14515	-5.35669	-1.03409	-5.56036
3.98657	-10.2483	11.73549	-3.77779	-0.29815	-5.31987
2.67342	-9.85197	10.6204	-5.61543	-2.2392	-7.66417
1.22053	-9.19833	12.55061	-4.98973	-3.54663	-6.65243
2.50884	-9.65711	13.66966	-3.23621	-4.02519	-8.38666
5.24056	-7.30842	12.34269	-4.8783	-4.1442	-9.01908
4.54457	-8.58653	13.32171	-3.86702	0.79474	-7.509
5.12212	-8.08053	10.13873	-5.46302	0.12466	-7.84275
3.50653	-8.29398	9.45458	-7.39108	1.38304	-6.9808
4.53515	-5.71731	10.74488	-7.13152	2.47575	-5.59482
2.90701	-5.95772	10.10474	-7.01865	0.72843	-5.37263
3.27024	-5.82574	12.92432	1.7604	2.36979	-4.79954
2.61845	-7.12626	13.91754	3.39905	1.69615	-5.01979
1.27048	-11.5315	14.70051	2.22164	0.86326	-3.95665
-0.14446	-11.0535	13.73853	3.78119	-7.21447	0.03906
0.50765	-12.7098	13.62323	3.76875	-6.34038	-1.5232
3.6315	7.42802	-2.39366	5.32211	-6.71913	-0.71752
4.60142	6.86302	-1.02697	5.5946	2.54427	6.41289
1.53343	6.76544	-1.18755	6.30294	0.90378	6.37012
2.48969	6.12387	0.14394	7.16934	2.27714	5.61282
3.35025	8.40416	0.66762	2.93295	11.7358	3.87144
1.59543	8.27224	0.77191	2.65809	11.9557	5.59967
0.87618	13.8223	1.96878	1.90618	10.58099	4.74467
1.99192	12.47504	1.92522	-2.24051	-3.47312	-10.6022
-0.72802	12.5827	3.31064	-3.83775	-3.65665	-11.3559
0.15264	11.0636	3.10742	-2.96502	-2.12725	-11.522

3.39531	-4.4294	15.07381
4.40329	-5.08108	16.39365
2.83301	-5.81333	16.03444
-4.34673	8.94142	-0.21493
1.72284	-11.2932	12.66943
4.5301	-5.93628	8.6821
3.48624	-8.16786	11.52954
4.36982	-6.32724	14.70776
1.05359	12.5349	4.3833
0.38014	12.30679	0.62994
2.22592	9.37292	-0.88143
-3.43085	12.36103	0.06255
-1.67818	-3.21629	-4.01472
-3.78128	-1.77556	-6.80572
-3.8803	-2.42364	-9.66126
-5.35488	1.62107	-6.304
-3.20299	5.93779	-3.1213
-2.646	8.0658	-3.9992
-2.97736	3.58048	-7.29382
0.50611	5.84602	-7.87192
0.17763	3.68956	-6.39701
1.9826	3.89238	2.35053
0.42988	-0.25871	-1.78242
0.14882	4.63529	0.51953
-0.9697	0.5671	-3.77649
-1.07053	5.55013	-1.47294
-2.97038	10.42083	-2.35304
3.80975	4.33045	-1.16569
5.71359	-1.05455	5.06611
3.14296	-4.08626	-2.2013
2.97734	2.66415	-3.21684
5.45441	1.64258	4.60447
4.18084	-5.23367	0.20707

Figure 8e Configuration

9.47183	4.50856	-2.31721
9.27112	5.91188	-2.27116
10.40639	3.88278	-1.48853
10.07355	6.66709	-1.3796
11.17504	4.6401	-0.60943
11.01186	6.02151	-0.56503
7.81933	4.29582	-4.22097
7.59164	5.70074	-4.16496
8.7284	3.65574	-3.27859
8.27383	6.54053	-3.17435
6.30298	4.13656	-6.14236
6.01856	5.48983	-6.04729
7.17965	3.52441	-5.20299
6.68026	6.2861	-5.0649
5.46406	4.14342	-8.49981
4.51051	5.27537	-8.12194
5.74263	3.2916	-7.26054
4.99634	6.16299	-6.95202
2.87099	7.30673	-6.66841
1.72968	7.35833	-5.65344
3.73743	9.43986	-5.92481
2.06791	8.25631	-4.45533
2.53618	9.6319	-4.98914
6.77489	4.60667	-9.17548
6.73154	5.74322	-10.189
9.07519	8.61412	-0.45059
4.2745	10.72732	-6.53091
0.41104	-1.20902	-1.79651
4.90279	7.10867	-0.20336
5.69211	-2.67641	4.66499
5.39553	-3.19843	6.06452
4.48316	-2.16993	-0.50124
3.21992	-2.29564	-1.07005
5.35363	-1.11792	-0.82141
2.79171	-1.33156	-1.99919
4.93827	-0.16803	-1.7754
3.66383	-0.30314	-2.34508
6.70874	-1.07665	-0.12233
5.79746	1.00925	-2.22501
5.9215	3.93471	0.26642
4.88369	4.73438	-0.1874
6.26168	2.71452	-0.34305
4.1386	4.32248	-1.31351
5.52723	2.30802	-1.46962
4.48348	3.12653	-1.94016
7.41553	1.91505	0.25416
6.60835	0.77572	3.80467
6.29444	-0.58962	3.63838
6.96671	1.52476	2.69323
6.34692	-1.135	2.35667
7.01851	0.98677	1.39694
6.69698	-0.36972	1.22985
5.08048	-4.69912	6.07299
4.77907	-5.23276	7.47547
3.00622	-10.6534	9.81208
2.23426	-11.0443	11.07663
4.82831	-9.40773	10.87448
4.01515	-8.75	8.65686

4.22938	-7.23641	8.76348	5.90301	5.55984	-10.8886
4.61784	-8.53008	12.11388	8.55519	5.236	-10.4294
0.82961	-12.7725	12.06315	9.20531	8.25104	0.57745
4.07135	8.25154	0.36356	8.06088	8.40362	-0.80094
2.55437	8.029	0.21685	9.25363	9.69061	-0.4819
1.72	9.31267	0.28587	7.98788	1.9429	-4.58015
-2.10779	12.98825	-0.79436	6.98158	7.98248	-4.31755
-3.41753	12.18153	-0.94378	1.43405	9.96319	-6.55959
-0.8477	12.83328	-2.95334	3.48938	11.26838	-7.06703
-0.33763	11.26478	-1.11393	5.08906	10.50667	-7.22729
1.18566	11.40732	-0.92766	4.66122	11.38093	-5.74118
-1.71121	11.99194	-3.93808	0.87385	-12.207	10.11213
-1.29602	13.28567	-5.98983	3.74071	-6.85356	6.82676
0.1719	0.27517	-1.52259	6.59894	-8.34673	12.53637
-1.04926	0.55325	-0.62729	-5.36883	12.46827	-0.4325
-2.40849	0.12237	-1.19565	2.87283	10.2916	-1.07261
-4.05941	0.4997	-2.96026	-0.33814	11.57652	-5.3907
-4.32919	1.32422	-4.23409	-2.04068	0.63896	-3.13171
-6.79659	1.29376	-4.12194	-5.6402	1.82267	-7.25859
-5.61336	-0.17388	-5.70505	-8.14562	3.75033	-2.35091
-4.89633	-0.02676	-7.05117	0.51693	-1.77183	-0.8622
-6.9346	2.74519	-3.63937	-0.41577	-1.64762	-2.36371
-9.33421	3.20039	-3.89972	5.97845	7.30574	-0.07457
2.33536	4.78717	-2.82578	4.7016	6.99868	-1.27485
2.21394	-4.34246	-1.69478	4.82992	-2.83166	3.99943
5.38019	1.54629	5.68048	6.55308	-3.20379	4.22857
-4.38157	12.18616	1.31592	6.26169	-2.98982	6.7053
-4.82145	1.33392	-9.06827	4.55225	-2.63124	6.47947
5.60163	-7.84428	14.23408	4.78769	-2.93474	0.20817
10.52335	2.80795	-1.5632	3.32201	0.40858	-3.09131
11.90793	4.15819	0.03128	7.46007	-0.62932	-0.7771
11.61733	6.63985	0.09043	7.03475	-2.11082	0.03964
3.57936	4.7862	-7.81554	6.85765	0.75463	-2.16756
4.27456	5.88354	-9.00014	5.59921	1.18651	-3.28807
4.80117	2.81127	-6.96278	6.47624	4.28486	1.13276
6.45435	2.49494	-7.49217	3.93344	2.80918	-2.81932
5.42844	7.09456	-7.33564	8.14953	2.63019	0.6441
2.53285	6.87155	-7.6166	7.93715	1.3613	-0.53036
1.49008	6.34683	-5.31702	7.22579	2.56615	2.8637
0.83661	7.74929	-6.15854	6.12375	-2.18666	2.21497
4.53216	8.92614	-5.3753	4.2174	-4.89625	5.4187
2.91905	7.81299	-3.92212	5.92128	-5.26962	5.65797
2.82126	10.27234	-4.14596	3.94113	-4.65733	7.91792
0.17295	8.79487	-3.96645	5.64748	-5.05703	8.12524
1.20495	8.83309	-2.69425	3.69315	-11.4786	9.54821
5.30726	2.58316	-9.67942	2.28718	-10.5659	8.9869
6.4896	6.67621	-9.64926	1.56258	-10.2099	11.35627

2.93008	-11.1797	11.9149	-3.81648	0.17294	-6.89082
5.7464	-9.08762	10.35646	-4.95067	-0.98737	-7.58294
5.017	-10.4368	11.2076	-6.15236	2.96806	-2.90501
4.90539	-9.20007	8.17891	-6.77177	3.41866	-4.50233
3.17063	-8.92765	7.97826	-9.15169	4.01944	-4.62083
5.08608	-7.02341	9.4161	-10.2333	3.44238	-3.3234
3.34421	-6.78579	9.25045	-9.55287	2.2962	-4.47791
4.4089	-7.48962	11.79758	1.58464	5.57463	-2.92088
3.72036	-8.8718	12.64505	2.93644	4.74278	-3.74367
1.55464	-13.0078	12.85139	1.84299	3.81967	-2.6615
0.11229	-12.0398	12.47941	1.55245	-5.09368	-1.25644
0.28272	-13.692	11.82918	1.74948	-3.91656	-2.59014
4.39421	9.16055	-0.16602	3.16793	-4.81297	-1.96735
4.32686	8.40699	1.41996	5.58412	2.05723	6.62474
2.34221	7.53154	-0.73611	4.72226	2.17331	5.06367
2.2248	7.33815	0.99979	4.88996	0.58847	5.88134
1.97588	9.89145	1.19513	-3.47782	12.55775	1.81088
0.66129	9.04332	0.37406	-5.23939	12.55341	1.88919
-2.31212	14.02525	-1.08782	-4.36649	11.08128	1.38312
-1.8386	13.02052	0.27012	-3.76391	1.62158	-8.90591
-3.78357	12.27515	-1.97143	-4.82402	0.47282	-9.74848
-3.21241	11.10647	-0.77418	-5.32484	2.16098	-9.58051
-1.07194	13.90024	-3.07756	5.4007	-6.77122	14.0521
0.20162	12.70642	-3.2574	6.50836	-7.92449	14.84308
-0.79929	10.95258	-0.17083	4.76708	-8.23884	14.82602
-0.51939	10.4456	-1.82869	0.94349	8.29073	-3.52537
1.38146	11.99278	-0.01117	1.5407	-12.3131	10.87561
1.58256	11.9905	-1.76553	4.52846	-6.67172	7.44948
-2.75161	12.3435	-3.91793	3.68988	-9.36556	9.94323
-1.72973	10.94929	-3.59558	5.7538	-8.64272	13.02345
-0.64583	14.06276	-5.54832	-4.45628	12.69905	-0.04843
-0.99907	13.16299	-7.03768	-0.94158	12.5241	-1.53013
-2.32274	13.67434	-5.98259	1.87943	10.11326	-0.93657
0.07656	0.8034	-2.48097	-1.27001	11.98645	-5.32939
1.06418	0.68854	-1.03638	-2.74444	0.8421	-2.42276
-0.9065	0.04998	0.33909	-5.59478	1.07515	-4.93104
-1.09174	1.6292	-0.42063	-5.5462	0.99941	-7.85203
-3.18362	0.35853	-0.45488	-8.22517	2.95735	-2.98174
-2.4395	-0.97809	-1.32442	3.88866	6.50023	-6.09408
-4.80497	0.74336	-2.19276	3.32792	8.59865	-7.03565
-4.17239	-0.58171	-3.16951	4.78632	3.38792	-9.51546
-4.26083	2.38541	-3.97159	7.94684	5.85711	-10.8751
-3.51715	1.14109	-4.9494	7.82728	4.01487	-8.99762
-7.65535	1.05697	-4.75891	10.04947	8.03122	-1.32064
-6.8674	0.62736	-3.2441	8.91264	2.41946	-3.28601
-6.65894	-0.42101	-5.91424	8.01696	7.76153	-3.10096
-5.1856	-1.02768	-5.15136	7.34912	2.19753	-5.30369

6.42889	7.60168	-5.0621
1.47106	10.32317	-5.65722
4.56628	5.89456	0.48339
5.98093	-1.28447	4.77303
1.56955	-1.4231	-2.63076
3.13234	5.15469	-1.70582
6.6481	1.36326	5.04801
2.412	-3.34673	-0.68572
